# Complete genome sequence of *Enterococcus faecium* strain TX16 and comparative genomic analysis of *Enterococcus faecium* genomes

**DOI:** 10.1186/1471-2180-12-135

**Published:** 2012-07-07

**Authors:** Xiang Qin, Jessica R Galloway-Peña, Jouko Sillanpaa, Jung Hyeob Roh, Sreedhar R Nallapareddy, Shahreen Chowdhury, Agathe Bourgogne, Tina Choudhury, Donna M Muzny, Christian J Buhay, Yan Ding, Shannon Dugan-Rocha, Wen Liu, Christie Kovar, Erica Sodergren, Sarah Highlander, Joseph F Petrosino, Kim C Worley, Richard A Gibbs, George M Weinstock, Barbara E Murray

**Affiliations:** 1 Human Genome Sequencing Center, Baylor College of Medicine, One Baylor Plaza MSC-226, Houston, TX, USA; 2 Department of Molecular Virology and Microbiology, Baylor College of Medicine, One Baylor Plaza MSC-226, Houston, TX, USA; 3 Department of Medicine, Division of Infectious Disease, Houston, TX, USA; 4 Center for the Study of Emerging and Reemerging Pathogens, Houston, TX, USA; 5 Department of Microbiology and Molecular Genetics, University of Texas Medical School, 6431 Fannin Street, Houston, TX, 77030, USA; 6 The Genome Institute, Washington University, 4444 Forest Park Avenue, Campus Box 8501, St. Louis, MO, 63108, USA

## Abstract

**Background:**

Enterococci are among the leading causes of hospital-acquired infections in the United States and Europe, with *Enterococcus faecalis* and *Enterococcus faecium* being the two most common species isolated from enterococcal infections. In the last decade, the proportion of enterococcal infections caused by *E. faecium* has steadily increased compared to other *Enterococcus* species. Although the underlying mechanism for the gradual replacement of *E. faecalis* by *E. faecium* in the hospital environment is not yet understood, many studies using genotyping and phylogenetic analysis have shown the emergence of a globally dispersed polyclonal subcluster of *E. faecium* strains in clinical environments. Systematic study of the molecular epidemiology and pathogenesis of *E. faecium* has been hindered by the lack of closed, complete *E. faecium* genomes that can be used as references.

**Results:**

In this study, we report the complete genome sequence of the *E. faecium* strain TX16, also known as DO, which belongs to multilocus sequence type (ST) 18, and was the first *E. faecium* strain ever sequenced. Whole genome comparison of the TX16 genome with 21 *E. faecium* draft genomes confirmed that most clinical, outbreak, and hospital-associated (HA) strains (including STs 16, 17, 18, and 78), in addition to strains of non-hospital origin, group in the same clade (referred to as the HA clade) and are evolutionally considerably more closely related to each other by phylogenetic and gene content similarity analyses than to isolates in the community-associated (CA) clade with approximately a 3–4% average nucleotide sequence difference between the two clades at the core genome level. Our study also revealed that many genomic loci in the TX16 genome are unique to the HA clade. 380 ORFs in TX16 are HA-clade specific and antibiotic resistance genes are enriched in HA-clade strains. Mobile elements such as IS*16* and transposons were also found almost exclusively in HA strains, as previously reported.

**Conclusions:**

Our findings along with other studies show that HA clonal lineages harbor specific genetic elements as well as sequence differences in the core genome which may confer selection advantages over the more heterogeneous CA *E. faecium* isolates. Which of these differences are important for the success of specific *E. faecium* lineages in the hospital environment remain(s) to be determined.

## Background

Enterococci are normal constituents of the gastro-intestinal flora of humans and other animals [1-3]. Although they only occasionally cause infections in healthy individuals, they are the third most commonly isolated gram positive organisms from hospital-associated (HA) infections in the United States and are increasingly reported in other countries [4,5]. Enterococcal infections are often difficult to treat due to the number of antibiotics to which these organisms are resistant. Some antibiotic resistances are intrinsic, such as resistances to cephalosporins, while other antibiotic resistances are acquired through mutations or horizontal gene transfer, most notably the *van* systems that encode vancomycin resistance [6-12]. Several recent studies also confirmed that enterococci can transfer their resistance to even more virulent organisms, such as *Staphylococcus aureus*[13].

*Enterococcus faecalis* is the most common enterococcal species recovered from infections. However, in the last decade, infections with *Enterococcus faecium* have been on the rise in the United States, Europe, and South America [2-5,14]. In the US, isolates of *E. faecium* now account for ca. 35% of nosocomial enterococcal isolates identified to the species level [4]. It is still not clear what has caused the ecological replacement of *E. faecalis* with *E. faecium* in the nosocomial setting, but it is speculated that the intense use of antibiotics in hospitals and the multiple antibiotic resistances of *E. faecium* have been major contributing factors [11,15]. A few genes have been suggested as being virulence determinants in *E. faecium* due to their enrichment in clinical isolates, such as the *fms* or *hyl* genes [16-22]. However, only three genes have been experimentally implicated to have an impact on virulence in animal models, namely *esp*, which has a role in biofilm, urinary tract infection, and endocarditis [23,24]; *acm*, encoding a collagen binding adhesin contributing to endocarditis [25,26]; and the *ebp*_*fm*_ operon which encodes pili that are important in biofilm and urinary tract infection [27]. In addition, conjugative transfer of a plasmid with a *hyl*-like gene not only conferred increased resistance to vancomycin but also increased virulence in transconjugants in the mouse peritonitis model [28], and a different *hyl*-plasmid conferred colonization in the murine gut [29]. While the gene(s) responsible for this increase in virulence and colonization have yet to be determined, the deletion of the *hyl* gene did not cause attenuation in the peritonitis model [19].

Molecular epidemiological studies of outbreaks of *E. faecium* using MLST initially indicated that there was a specific lineage or genogroup of strains, designated clonal complex 17, that was predominant in the hospital environment [2,5,15,30]. Other studies using pyrosequencing and whole-genome microarray subsequently indicated that, while there appeared to be a globally dispersed clade containing the vast majority of epidemic and clinical isolates which harbor a large content of accessory genes specific to this clade [31,32], isolates associated with healthcare settings were not strictly clonally related to each other. In particular, while CC17 genogroup isolates are part of the HA subpopulation, not all HA isolates are considered part of the ST17 lineage [33]. Recent studies in our laboratory and others have shown large differences (~3–4%) in the sequence of the core genome, as well as differences in the 16-S rRNA, between two different clades which were named the hospital-associated clade (HA) and community-associated (CA) clade strains, (also known as clade A and B [34])[32,33]. The HA clade contains most clinical and HA-associated strains but also included strains from non-hospital origin [35,36].

Molecular studies and comprehensive comparative genomic studies of *E. faecium* have long been hindered by the lack of a complete genome sequence. The TX16 (DO) genome was initially sequenced at the Department of Energy’s Joint Genome Institute (JGI) in Walnut Creek, Ca. in 1999 in an effort to demonstrate capabilities of the sequencing technology at that time by sequencing the genome in only 1 day. However, the genome was far from closed and the past decade has been spent on annotation, final assembly, and analyses of this genome. Recently, while this manuscript was in review, a closed *E. faecium* genome was published by Lam et al. using the ST17 isolate Aus0004, which was isolated from the bloodstream of a patient in Melbourne, Australia [37].

In this study, we report the closed genome of the US *E. faecium* endocarditis isolate TX16 (DO), and a comparative analysis of this strain’s genome with 21 other available *E. faecium* draft genomes [32,38], as well as the recently published Aus0004 [37]. Due to the fact the TX16 genome has been used in multiple pathogenesis studies and is a part of the clonal group representing the majority of clinical strains globally [2,5,30,36], the complete genome sequence of *E. faecium* TX16 will facilitate future research by providing a critical starting point for genome-wide functional studies to determine the molecular basis of pathogenesis and to further understand the evolution and molecular epidemiology of *E. faecium* infective strains.

## Results

### *E. faecium* TX16 general genome features

**Figure 1:**
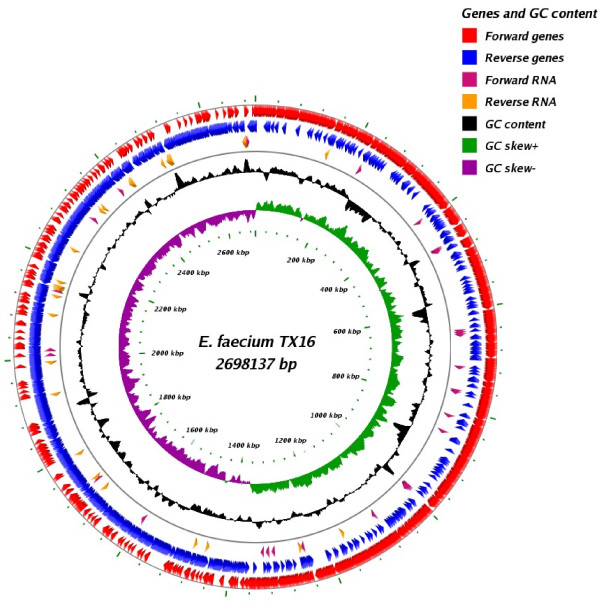
**Circular map of the*E. faecium*TX16 genome.** Tracks from inside to outside are as follows: GC skew (G-C)/(G + C), GC content, forward and reverse RNA, reverse genes, and forward genes.

The *E. faecium* TX16 genome consists of one chromosome and three plasmids. The chromosome (Figure 1) contains 2,698,137 bp with 2,703 protein-coding ORFs, 62 tRNAs, 6 copies of ribosomal rRNA and 32 other non-coding RNAs (Table 1). The chromosome has a GC content of 38.15%, and it shows a clear GC skew at the origin of replication (Figure 1). The sizes of the three plasmids (pDO1, pDO2, and pDO3) are 36,262, 66,247 and 251,926 bp, encoding 43, 85, and 283 ORFs, respectively (Table 1).

**Table 1:** **General features of*E. faecium*TX16 genome**

**Features**	**Chromosome**	**Plasmid pDO1**	**Plasmid pDO2**	**Plasmid pDO3**
**Size (bp)**	2698137	36262	66247	251926
**G + C %**	38.15	36.51	34.38	35.97
**ORFs**	2703	43	85	283
**rRNA operons**	6	0	0	0
**tRNAs**	62	0	2	0
**ncRNAs**	32	1	0	0

To investigate the conservation of the gene order of *E. faecium* compared to its close relative *E. faecalis,* a BLASTP alignment of all the predicted proteins from the TX16 and V583 genomes was performed followed by ORF synteny analysis using DAGchainer [39]. The result showed that *E. faecium* TX16 gene order is very different from that of *E. faecalis* strain V583 (and therefore OG1RF, which has a very similar synteny to V583 [40,41]) and all ORF synteny blocks were relatively short (Additional file 1: Figure S1).

Interestingly, when comparing TX16 to the closed genome Aus0004, which was published while this paper was in review, Mauve genome alignment analysis resulted in 5 locally collinear blocks for both TX16 and Aus0004 ranging from 33,563–836,291 bp for TX16 and 32,326–905,025 bp for Aus0004 (Additional file 2: Figure S2). The two isolates had very similar synteny, although two regions found in TX16 were inverted in Aus0004. Two site-specific tyrosine family recombinases (EFAU004_01466 and EFAU004_02416) were found flanking these two inversions (Additional file 2: Figure S2).

**Figure 2:**
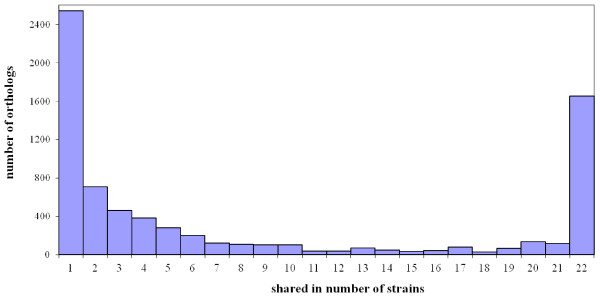
**Distribution of orthologs in 22*E. faecium*strains.** The orthologs were determined by orthoMCL as described in the Material and Methods. ORFs of the 3 plasmids in *E. faecium* TX16 were not included in the ortholog analysis.

**Figure 3:**
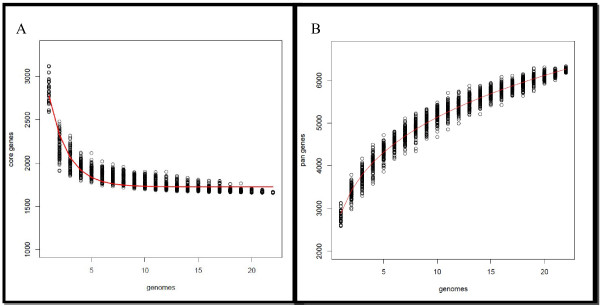
***E. faecium*core and pan genomes.A**. *E. faecium* core genes. The number of shared genes is plotted as the function of number of strains (n) added sequentially. An open circle represents the number of shared genes for each permutation at a give number of strains (n). 1,608 single copy genes are shared by all 22 genomes. The red line represents the least-squares fit to the exponential decay function *F*_*c*_ = *κ*_c_ exp[−*n*/*τ*_*c*_] + *Ω* (*κ*_c_ = 1871 ± 25, *τ*_*c*_ = 1.751 ± 0.027, *Ω* = 1726 ± 2). **B**. *E. faecium* pan-genes. The number of total genes is plotted as the function of strains (n). The open circle represents the number of total genes for each permutation at a give number of strains (n). The red line represents the least-squares fit to the power law function *n* = *κ N*^γ^ (*κ* = 2876 ± 7, *γ* = 0.2517 ± 0.009).

The genome size of the *E. faecium* strains vary substantially from 2.50 Mb (E1039) to 3.14 Mb (1,230,933), while the number of ORFs varies from 2,587 (E1039) to 3,118 (TX0133A). Ortholog analysis of TX16 compared to TX1330 and all the available but unfinished *E. faecium* genomes using BLASTP of predicted protein sequences and orthoMCL resulted in 3,169 distributed genes shared among some strains (Figure 2), 2,543 unique genes (Figure 2), and 1,652 core gene families, of which 1,608 genes are present in a single copy in all strains and 44 gene families are present in multiple copies. The number of core genes (including those in single and multiple copies) converged to 1,726 at the 22^nd^ genome, while the number of pan genes reached 6,262 genes at the 22^nd^ genome (Figure 3A and B). The extrapolated number of core genes is very close to the number of core genes (1,772 genes) Leavis et al. reported in their microarray-based study which used 97 isolates, yet the estimated number of pan genes is higher in the present analysis [31]. Furthermore, this study differs slightly from the analysis of van Schaik et al. which estimates the *E. faecium* core genome to be 2172 ± 20 CDS [32]. Our data do, however, concur with the conclusion that a sizeable fraction of the *E. faecium* genome is accessory and that the pan genome is considered to open.

### Phylogenetic, multi-locus sequence typing (MLST) and gene content similarity analysis

**Figure 4:**
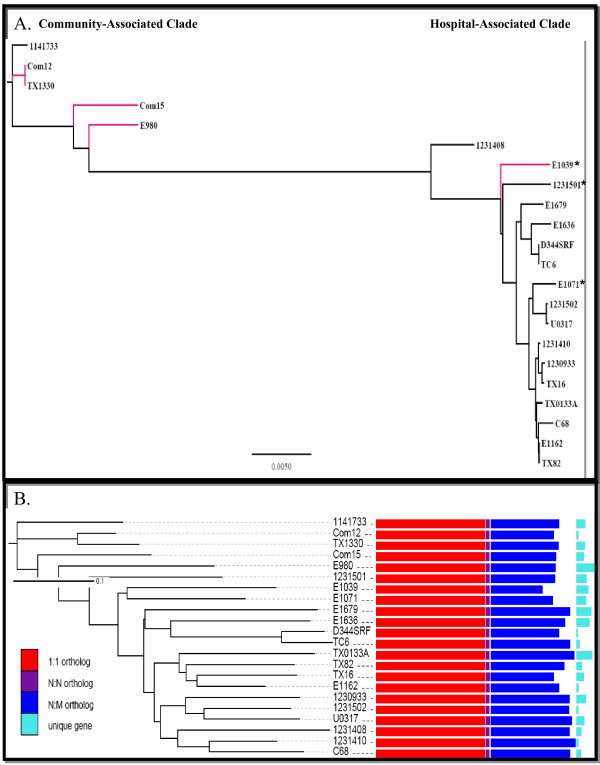
***Enterococcus faecium*phylogenetics.** 4**A**. A maximum-likelihood phylogenetic tree using 628 core genes. Distance bar indicates the sequence divergence. Strains isolated from the community are labeled with branches in red. An asterisk (*) indicates a strain within the HA clade lacking IS*16*. 4**B**. A hierarchical clustering using Jaccard distance of gene content by unweighted pair group method with arithmetic mean (UPGMA) (see Materials and Methods). The core, distributed and unique gene counts are also presented in the right panel. 1:1 ortholog, orthologs present with one copy in all strains; N:N ortholog, orthologs present with multiple copies in all strains; N:M ortholog, orthologs present in some strains.

Analysis of the 22 *E. faecium* genomes (Table 2) showed that the isolates separate into two clades, one branch consisting mostly of CA isolates, with most HA isolates found in the other, as was noted in our previous study [33] (Figure 4A and B). When analyzing the phylogenetic distances among these 22 isolates using 628 single-copy ortholog genes of the same length (Figure 4A), similar clade patterns were observed for the *E. faecium* strains as seen in the 100 core gene analysis by Galloway-Pena et.al [33]. All isolates predicted to be part of the CC17 genogroup [2,5,30] cluster more closely together and branched more distantly than other HA-clade isolates (Figure 4A). The dendogram construction from the gene content dissimilarity represented by Jaccard distance (Figure 4B) also showed most hospital-isolated strains cluster together except hospital- isolated strain 1,141,733 which was shown genetically to belong to the CA clade. In addition, although E1039 is a community- isolated fecal strain, it is genetically closer to the HA strains. The phylogenetic and gene content dissimilarity analysis results all support the existence of two very distinct clades of *E. faecium*, which has been previously described using pyrosequencing, microarray, and the concatenation of a 100 core genes, estimated to have diverged anywhere from 300,000 to 3 million years ago [31-33].

**Table 2:** **The 22 sequenced*Enterococcus faecium*genomes**

**Strain**	**ST**	**CC17**	**Country**	**Year**	**Source**	**Reference**
1,231,408^a^	582	Yes	NA^b^	NA	Blood Culture of Hospitalized Patient	[38]
1,231,501	52	No	NA	NA	Blood Culture of Hospitalized Patient	[38]
Com15	583	No	USA (MA)	2006	Healthy Volunteer Feces	[38]
1,141,733	327	No	NA	NA	Blood Culture of Hospitalized Patient	[38]
1,230,933	18	Yes	NA	NA	Wound Swab of Hospitalized Patient	[38]
1,231,410	17	Yes	NA	NA	Skin and Soft Tissue Infection	[38]
1,231,502	203	Yes	NA	NA	Blood Culture of Hospitalized Patient	[38]
Com12	107	No	USA (MA)	2006	Healthy Volunteer Feces	[38]
E1039	42	No	Netherlands	1998	Healthy Volunteer Feces	[32]
E1162	17	Yes	France	1997	Blood Culture of Hospitalized Patient	[32]
E1071	32	No	Netherlands	2000	Hospitalized Patient Feces	[32]
E1679	114	No	Brazil	1998	Swab of Vascular Catheter	[32]
E1636	106	No	Netherlands	1961	Blood Culture of Hospitalized Patient	[32]
E980	94	No	Netherlands	1998	Healthy Volunteer Feces	[32]
U0317	78	Yes	Netherlands	2005	UTI of Hospitalized Patient	[32]
D344SRF^c^	21	No	France	1985	Clinical (Site not specified)	[42]
TC6	21	No	USA (OH)	NA	Transconjugant of C68 and D344SRF	[29]
C68	16	Yes	USA (OH)	1998	Endocarditis Patient (Feces)	[9]
TX0133	17	Yes	USA (TX)	2006	Endocarditis Patient (Blood)	This study
TX82	17	Yes	USA (TX)	1999	Endocarditis Patient (Blood)	[25]
TX16	18	Yes	USA (TX)	1992	Endocarditis Patient (Blood)	[43]
TX1330	107	No	USA (TX)	1994	Healthy Volunteer Feces	[17]

^a^Hybrid genome with ~1/3 of the core genes from the CA clade and 2/3 from the HA clade.

^b^Indicates this information was not available.

^c^A rifampin- and fusidic acid-resistant derivative of clinical strain *E. faecium* D344S in which the spontaneous loss of *pbp5* and its surrounding region resulted in an ampicillin-susceptible phenotype.

**Figure 5:**
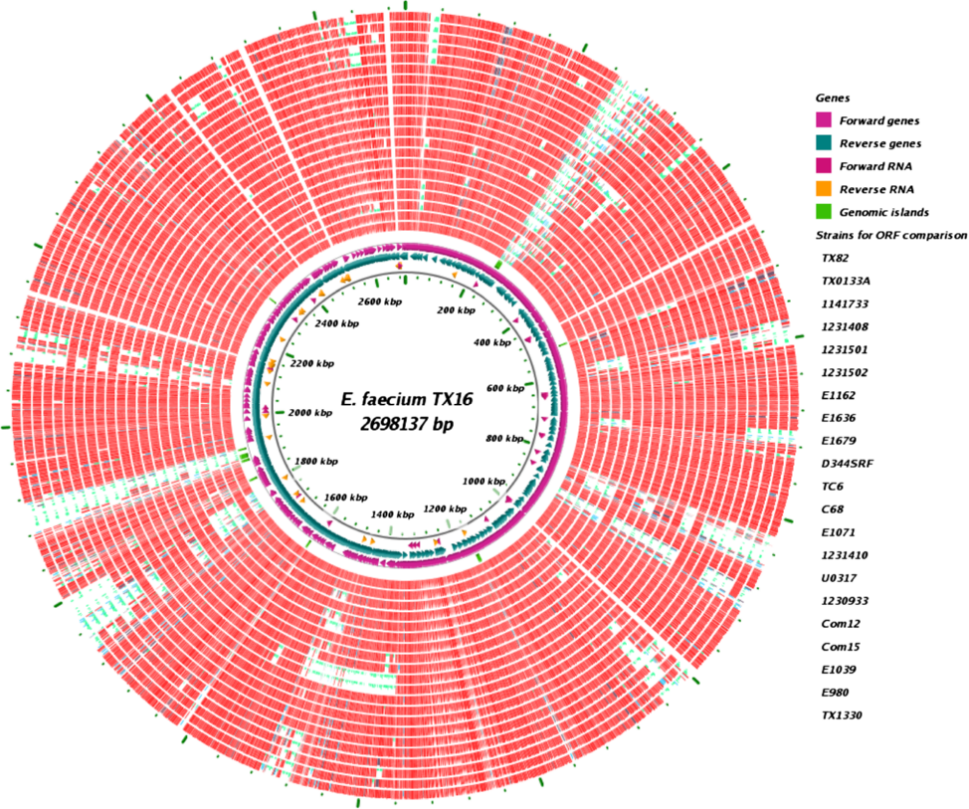
**ORF comparisons of the 22*E. faecium*genomes.** A circular map of BLASTP identity of predicted proteins from TX16 against the predicted proteins from other 21 *E. faecium* strains. Tracks from inside to outside: forward and reverse RNAs, reverse genes, foward genes, and genomic islands. In outer strain circles from inside to outside are the BLASTP precent identity of TX16 against ORFs from TX82, TX0133A, 1,141,733, 1,231,408, 1,231,501, 1,231,502, E1162, E1636, E1679, D344SRF, TC6, C68, E1071, 1,231,410, U0317, 1,230,933, Com12, Com15, E1039, E980, and TX1330. Red is 90–100% identity, purple is 60–89% identity, green is 0–59% identity.

Comparison of *E. faecium* TX16’s predicted proteins to predicted proteins from the other 21 *E. faecium* genomes using BLASTP revealed a mosaic-like structure, as previously described [16,33], and many highly variable regions. Some of the TX16 variable regions are HA clade specific (Figure 5). Notably, regions from 27 to 38 kb, from 581 to 606 kb, from 702 to 717 kb, from 997 to 1,042 kb, from 1,737 to 1,802 kb and from 2,629 to 2,642 kb on the TX16 genome are missing or have low identity in the CA strains. Interestingly, region 1737 to 1802 kb encodes 4 surface proteins (HMPREF0351_11775, HMPREF0351_11776, and HMPREF0351_11777 which are the 3-gene pilus cluster, *fms11-fms19-fms16* and HMPREF0351_11828 which is *fms18*, also known as EcbA, a collagen and fibrinogen binding MSCRAMM). Another notable region with low ORF identity hits or missing in strain D344SRF and TC6 is a ~145-kb region from 1,364 to 1,509 kb on the TX16 genome. Containing the pilus subunit protein EbpCfm (*fms9*) and other 2 pilus subunit proteins (EbpAfm and EbpBfm)(Figure 5).

Assessment of genomic rearrangements among *E. faecium* strains was more difficult because other genomes are not complete. We further investigated the genes that are unique to the HA-clade based on clade assignment of the strains in the phylogenetic analysis, and identified 378 ORFs (14% of TX16 ORFs) that are unique to the HA clade (shared at least between 2 HA clade isolates) (Additional file 3: Table S1). Of the 378 ORFs, 282 ORFs are conserved in at least half of the HA clade strains including 61 ORFs which are shared among all HA-clade isolates. Most of the HA clade unique genes are transposon-related genes, transporters, and prophage genes. Interestingly, a Cna B-type gene, the enterocin A operon, and two *fms* genes (see MSCRAMMs below) are among the HA-clade specific genes. Strain 1,231,408 was excluded from the HA unique gene analysis because it was previously shown to be a hybrid strain that contained both HA (~2/3) and CA (~1/3) alleles based on our 100 core gene analysis [33].

### Mobile genetic elements

*E. faecium* isolates from patients typically have many mobile genetic elements which often contain antibiotic resistance genes that are easily transferable between strains. Bacteriophage-mediated transduction can transfer antibiotic resistance between enterococci [44,45] and many bacteriophages have also been identified in *E. faecium*[44]. To identify phage genes on the TX16 genome, Prophinder and Prophage Finder were used to search for prophage loci [46,47]. Both programs identified that two chromosomal regions (821–858 kb and 2,073–2,088 kb) with a total size of about 62 kb contain phage-related genes. Sixty-one and twenty one phage-related genes were identified in these regions, respectively (Additional file 4: Table S2). All CA strains have low identity ORF hits or missing ORFs in the predicted prophage locus from 821 to 857 kb, while most HA strains have similar ORFs in this locus. All CA strains and most HA strains lack similar ORFs in the other predicted prophage locus from 2,073 to 2,087 kb (Figure 5 and Additional file 3: Table S1). In addition to these two main regions, small numbers of phage-related genes were also identified throughout the chromosome, but these were not further analyzed.

IS elements and transposases are major mobile genetic elements in *E. faecium* and about 180 IS element and transposase-related genes were identified in the TX16 genome (Additional file 5: Table S3). About half of these IS elements and transposases are present on the three plasmids. Considering the sizes of the chromosome and three plasmids (chromosome, 2,698,137 bp; plasmid 1, 36,262 bp; plasmid 2; 66,247 bp; plasmid 3, 251,926 bp), plasmid DNAs appear to be more susceptible to IS element/transposase insertions. Some IS elements/transposases exist as multiple copies in specific locations on the chromosome or plasmids. Four copies of ISEnfa3 sequence (HMPREF0351_10172, HMPREF0351_10364, HMPREF0351_11866, and HMPREF0351_11868) were identified in the chromosome but not in the 3 TX16 plasmids whereas the sequences of IS1216 (HMPREF0351_12707, _12726, _12729, _12749, _12763, _12794, _12807, _12813, _12818), IS1297 (HMPREF0351_12910, _12920, _12891, _12875), and ISEfa4 (HMPREF0351_13111) were identified in the three plasmids but not in the chromosome. IS elements and transposases were found more frequently in HA strains than in CA strains. Previously, IS*16* was suggested as a molecular screening marker to predict *E. faecium* pathogenicity because of its presence in clinical *E. faecium* isolates [31,48]. We performed a BLAST search of the 22 *E. faecium* genomes to identify the IS/transposase elements showing the same presence or absence patterns of IS16 (HMPREF0351_11812, _11855, _12352, and _12809). Many IS/transposase elements were found to have the same pattern of presence/absence in different strains as IS16; including ISEnfa3 (IS3/IS911 transposase: HMPREF0351_10172, _10364, _11866, and _11868), IS116/IS110/IS902 family transposases (HMPREF0351_11035, _11528, _12768, and _13088), IS66 transposases (HMPREF0351_10928, _11787, _11933, _12004, _12887, and_12948), and transposases (HMPREF0351_10878, _10880, _10927, _11934, and _12005). Therefore, all these IS elements and transposases (in addition to IS*16*) have potential as molecular markers to identify clinical *E. faecium*. However, these IS elements and transposases are not found in all HA-clade strains as 1,231,501; E1039; and E1071 do not have these IS elements and transposases, although they are present in all of the isolates considered to be part of the CC17 genogroup (Figure 4A).

### Genomic islands

A pathogenicity island containing the *esp* gene has previously been reported in *E. faecium*[32,49]. The *esp* gene is not present in the TX16 genome but a search for other possible genomic islands (GIs) in TX16 using GI prediction programs including IslandPath-DIMOB [50], SIGI-HMM [51], and IslandPick [52,53], identified a total of 9 regions totaling 62,290 bp predicted as GIs. The GIs are shown in Figure 5, and the genes encoded by GIs are listed in Additional file 4: Table S2 and Additional file 6: Table S4. GIs 6, 7 and 8 might be a single GI, since they are located very close together. GIs 6 and 7 are separated by only 2 ORFs and 7 ORFs are present between GIs 7 and 8. The 9 predicted GIs have hypothetical proteins and transposon-related proteins in common. Among these putative GIs, islands 2, 3, 4, and 5 were frequently present in *E. faecium* of HA origin (data not shown). Island 2 contains 9 genes (6 genes encoding hypothetical proteins, and a predicted transposase and two transcriptional regulators). Island 3 contains 12 genes including 4 hypothetical proteins, 3 predicted ABC transport genes, a transposase, a Mg-dependent DNase, a LysM family protein, a cell wall protein, and a predicted fosfomycin resistance protein. Island 4 and 5 are composed of 7 and 9 genes, respectively. Island 4 contains 5 hypothetical proteins, a putative membrane protein, and a putative transposase. Four hypothetical proteins and 5 transposase related proteins were present in Island 5. The presence of a transposase in each island supports that these islands were acquired through horizontal gene transfer. While a potential role in pathogenesis has been suggested, there are many hypothetical proteins in each island and no genetic or experimental evidence to indicate such a role. However, island 3 which contains a predicted fosfomycin resistance protein might be important in promoting *E. faecium* colonization because of the selective advantage conferred when this antibiotic is used. The remaining GIs 1, 6, 7, 8, and 9 exist only in the TX16 genome or in a limited number of *E. faecium* strains.

We also searched for previously reported GIs [17,49] and pathogenicity islands [32] in the 22 *E. faecium* genomes. As reported [32], a pathogenicity island including the *esp* gene was observed in E1162; E1679; and U0317. In addition to these three strains, an island with a partial *esp* gene was also found in 1,231,502; C68; 1,231,410; TX0133A; and 1,230,933 strains when we performed a BLAST search. The *esp* gene could possibly be intact in these strains but interrupted in the draft assemblies, possibly as a consequence of the next-generation sequencing technology problems. A GI previously found to be specific to CC17 [49] was also observed in the HA clade strains TX0133A; TX82; C68; 1,231,410; 1,230,933; E1162; TX16; 1,231,502; U0317; and E1679. Intrestingly, 1,231,408, which is the mosaic strain [33], lacked this GI. The presence of a putative three-gene pilus-encoding cluster, *fms11-fms19-fms16*, previously proposed as a small GI [17], is described within the subsequent section on MSCRAMM-like proteins.

### Genetic loci in *E. faecium* TX16 predicted to be involved in biosynthesis of surface polysaccharides

Our analysis of the *E. faecium* TX16 genome did not identify close homologs of the *cpsC-K* cluster of *E. faecalis*. Homologs of the two genes, *cpsA* and *cpsB*, were found and well conserved in TX16, but were recently reported to not be sufficient for capsule production in *E. faecalis*[54]. Similarly, homologs of *cpsA-cpsB* but not of *cpsC-K* were found in the 21 other *E. faecium* draft genomes.

**Figure 6:**
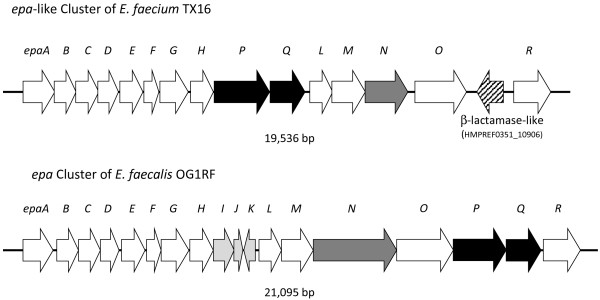
**Comparison of the homologous*epa-*like loci of*E. faecium*TX16 and*E. faecalis*OG1RF.** Orthologs of *epaP* and *epaQ*, located at different positions in the *E. faecium* and *E. faecalis* genomes, are indicated by black arrows. Genes *epaI, epaJ* and *epaK*, present only in *E. faecalis*, are indicated by light grey arrows. The *epaN* homolog of *E. faecium*, which is shorter than *epaN* of *E. faecalis*, is shown by a dark grey arrow. The TX16 ORF (HMPREF0351_10906) with relatively low similarity to the β-lactamase superfamily is shown by a hatched arrow. The *epaA* to *epaR* region of *E. faecium* TX16 corresponds to locus tags HMPREF0351_10891 to HMPREF0351_10907.

In contrast, a locus homologous to the *epa* locus, which was shown to produce a rhamnose, glucose, galactose, *N*-acetylgalactosamine and *N*-acetylglucosamine-containing antigenic cell wall polysaccharide in *E. faecalis* OG1RF[55,56], was found in the TX16 genome (Figure 6). However, identities of the encoded Epa-like proteins vary widely between orthologs of TX16 and OG1RF (ranging from 31% (EpaQ) to 92% (EpaE)). In addition, gene composition and order of the *epa*-like locus are partially different in these two organisms; the homologs of the three genes in the middle of the *E. faecalis epa* cluster, *epaI, epaJ* and *epaK,* are not present in TX16, while two other *epa*-like genes, *epaP* and *epaQ* are located at this site. All 15 *epa*-like genes of TX16 were found to be present, highly conserved and similarly organized in all 21 available *E. faecium* draft genomes (aa identities of the encoded proteins range from 88% to 100%), indicating that they are part of the core genome of this species. However, the absence of three *epa* genes in *E. faecium*, one encoding a glycosyl hydrolase (*epaI*), suggests the Epa polysaccharides of the two species have different sugar compositions.

Genes encoding proteins predicted to be an initiating transferase of polysaccharide biosynthesis (undecaprenylphosphate sugar phosphotransferase), glycosyl transferases, acetyl transferases, sugar phosphate transferases and repeat unit polymerases are typically clustered together in loci that mediate polysaccharide synthesis in gram-positive bacteria. Our search for these features in the TX16 genome identified two additional regions that might be involved in polysaccharide production.

The first of these regions found in TX16 (Locus 4) is a downstream extension of the *epa*-like region (HMPREF0351_10908 - HMPREF0351_10923), immediately preceded by an undecaprenyl-phosphate galactose-phosphotransferase (encoded by *epaR*) (Additional file 7: Figure S3). Unlike the *epa* region, however, the extension (HMPREF0351_10908 - HMPREF0351_10923; Locus 4) is present in only 5 of the other *E. faecium* draft genomes; all except one of these strains (E980) belong to the HA clade . This Locus was also observed in these strains by Palmer et al. [34]. TX16 and these 5 draft genomes also have an additional ORF (HMPREF0351_10906 in TX16), encoding a putative member of the large beta-lactamase-like superfamily (Pfam PF00144, e = 9.4 × 10^−17^) between *epaO* and *epaR* on the upstream side of this region (Figure 6) and a transposase (HMPREF0351_10924) in 5 of the 6 genomes on its downstream side.

Analysis of the remaining 16 draft genomes for a corresponding region revealed a predicted polysaccharide-encoding gene cluster downstream of the *epa* region in all of them, (Locus 1, 2, and 3 also described by Palmer et al. [34]), although these regions have only low similarities to those of TX16 and the 5 genomes above and extensive sequence variation among each other (Additional file 7: Figure S3). Locus 3 (HMPREFD9522_ 02513–02504) was found in only HA clade strains, while Locus 1 (EFWG_01379-01370) and Locus 2 (HMPREF0352_0048-0457), although found in some HA-clade strains, were only found in non-CC17 isolates as well as in four of the five CA-clade isolates, indicating some specificity of polysaccharide biosynthesis genes for certain lineages or niches. Of note, none of Locus 2 strains have IS*16*, only two of the Locus 1 strains have IS*16*, while all that had Locus 3 or 4 have IS*16*.

The second region found in TX16 that appears likely to be involved in polysaccharide biosynthesis (HMPREF0351_11938 - HMPREF0351_11970) is largely unique to this genome, with only the first four ORFs present in 20 of the genomes and the whole region completely absent in one of the genomes (E1039). However, each of the 20 other genomes has additional genes predicted to be involved in polysaccharide biosynthesis at this location.

### Distribution of genes encoding MSCRAMM-like proteins, putative virulence genes, antibiotic resistance determinants, and CRISPRs

Previous studies of *E. faecium* TX16 identified 15 genes encoding LPXTG family cell-wall anchored proteins with MSCRAMM-like features, such as immunoglobulin-like folding; 11 of these were found in four gene clusters, each predicted/demonstrated to encode a different pilus, and four were found as individual MSCRAMM-encoding genes [18,21,22]. Our search for these genes in 21 unique *E. faecium* draft genomes in this study found all of the MSCRAMM-encoding genes to be widely distributed except *fms18 (ecbA)* and *fms15* which were only in HA-clade isolates (although some are present as variants or pseudogenes within the HA-clade) (Additional file 8: Table S5). Moreover, our analysis revealed that *ebpA-ebpB-ebpC*_*fm*_*fms14-fms17-fms13fms20, scm*, and *fms18* (the latter present in only HA isolates) all have sequence variants in some of the 21 strains, with identities of the encoded variant proteins ranging from 39% (*fms20* homolog) to 94% (*ebpC*) versus their counterparts in TX16 (Additional file 8: Table S5). In general, most of the MSCRAMMS followed the CA/HA clade groupings with a variant representing each clade. Variant 1 of the *fms11-fms19-fms16* locus was strictly found in the HA-clade, and variant 2 in the CA-clade except for 1,231,501 which only had one of the three proteins (*fms16*) as a CA-variant, suggesting recombination by this isolate. Variant 1 of *fms14-fms17-fms13* was found in all but one HA clade isolate (1,231,408, a hybrid of HA and CA clades, has variant 2) and variant 2 in all 5 CA-clade strains. Variant 1 of *scm* was found to be exclusively carried by all 16 HA clade strains and variant 2 by 4 of the 5 CA clade strains. Although the differences between these MSCRAMMs in CA- vs. HA-clade strains are generally greater (ranging from 2 to 27% with an average of 10%) than the differences (3–4%) previously reported for the clade-specific differences in a set of core genes that excluded predicted surface proteins, they are comparable to the differences seen in several other surface proteins that have been studied [33,57].

Interestingly, the majority of HA clade strains (12/16, including TX16) were found to have variant 1 of the *ebp* pilus operon, while variant 2 was exclusively found in the 5 CA-clade strains in addition to variant 1 in three of the five isolates. In contrast, variation within *fms20* was restricted to the HA clade; all CA clade isolates carried *fms20* variant 1, but the percent identity between these two variants is much smaller (39%), possibly indicating the need for a new gene name. Also of note was the *acm* gene, which is present as a pseudogene in all of the CA-clade isolates except 1,141,733 which is the only CA-clade isolate that is from a hospitalized patient; *acm* pseudogenes were also found in non-CC17 HA-clade isolates.

Of note, our search for MSCRAMMs and potential pilus proteins also found one to three new individually encoded CnaB domain-containing MSCRAMMs in five of the *E. faecium* draft genomes and a new pilus encoding gene cluster in strain E1071; the latter consists of three genes one of which is a relatively distant homolog of *bee1* (35% aa identity) and two are identical or highly homologous to *bee2* or *bee3* (100% and 98%, respectively) of a plasmid-encoded *bee* pilus gene cluster found in a small percentage of *E. faecalis* isolates [58].

To identify possible virulence genes in the *E. faecium* genomes, the enterococcal virulence factors listed in the Virulence Factors Database (VFDB) [59] were aligned to the ORF protein sequences using BLASTP and filtered with 50% identity and 50% match length. The homologs of *efaA*, EF0954 (a homolog of BopD which is a transcriptional regulator involved in biofilm production of *E. faecalis*[42,60] ), *cpsA* and *cpsB* genes are present in all *E. faecium* strains (see surface polysaccharides above for *cpsA* and *cpsB*), and *esp*_*Efm*_ and *hyl*_*Efm*_ are exclusively present in some HA clade strains while the homolog of EF0818 (a putative hyaluronidase and annotated as a Family 8 polysaccharide lyase, also similar to the LPXTG protein EF3023) is exclusively present in the CA-clade strains (except strain 1,141,733). Homologs of other *E. faecalis* virulence factors listed in the VFDB were not found in TX16 genome.

We also searched the 22 *E. faecium* isolates for the presence and absence of 13 resistance genes. Our data correspond to previously published data for some of the isolates [32,61]. We observed that there is a clear distinction between the isolates of the genetically defined CA clade and those of the HA clade with none of the CA clade isolates having any of the antibiotic resistance determinants analyzed (Table 3). On the other hand, all of the HA-clade isolates have multiple resistance determinants, including the *pbp5*-R allele that confers ampicillin resistance previously reported by Galloway-Pena et al. [57], except for strains 1,231,501 and E1039. 1,231,501, which is in the HA-clade but lacks all antibiotic resistances including *pbp5*-R, may have lost the allele via recombination and acquired *pbp5*-S or may even represent a more ancestral isolate. Indeed, 1,231,501 was shown to be a hybrid of HA and CA genomes by Palmer, et al., with the replacement (hybrid) region including *pbp5-S*, which could explain the origin of *pbp5-S* in this strain [34]. E1039, which has the *pbp5*-R allele but none of the other resistance genes, is genetically defined as a HA-clade isolate, but came from a healthy volunteer, perhaps explaining its lack of other antibiotic resistances. Interestingly, neither of these strains has IS*16*. D344SRF is the only other HA-clade isolate that lacks the *pbp5*-R allele; however, this strain is known to have spontaneously lost *pbp5* and the surrounding region and contains many other resistances [62]. Of note, E1636 only has two of the 13 resistances analyzed (*tetM* and *pbp5-*R); however, this could possibly be explained by its early isolation in 1961. This again suggests that these isolates are more distantly related to the other strains within the HA-clade.

**Table 3:** **Antibiotic resistance gene profiles of the 21*E. faecium*strains**

**Gene**	***cat***	***ermA***	***ermB***	***aad6***	***aad9***	***aadE***	***aacA- aphD***	***tetL***	***tetM***	***vanA***	***gyrA***^***b***^	***parC***^***c***^	***pbp5*-R**^***d***^
**Resistance**	**CHL**	**ERY**	**ERY**	**SPC/ STR**	**SPC/ STR**	**SPC/ STR**	**GEN**	**TET**	**TET**	**VAN**	**CIP**	**CIP**	**AMP**
Strains													
1,141,733													
Com12													
Com15													
E980													
TX1330													
1,230,933			X	X		X	X		X	X	X	X	X
1,231,408			X	X		X	X				X	X	X
1,231,410			X	X		X				X	X		X
1,231,501													
1,231,502			X	X		X	X			X	X	X	X
C68			X	X		X	X		X		X	X	X
D344SRF^a^			X	X		X		X	X				
TX16	X		X	X		X		X	X				X
E1039													X
E1071	X		X	X	X	X		X		X			X
E1162								X	X				X
E1636									X				X
E1679		X	X	X	X		X			X	X	X	X
TX82			X	X		X			X	X	X	X	X
TX0133A	X		X	X		X	X		X		X	X	X
U0317			X	X		X	X				X	X	X

^*a*^A rifampin- and fusidic acid-resistant derivative of clinical strain *E. faecium* D344S in which the spontaneous loss of *pbp5* and its surrounding region resulted in an ampicillin-susceptible phenotype.

^*b*^Amino acid change (E to K/G) in residue 87 or (S to R/Y/I) in residue 83 of GyrA.

^*c*^Amino acid change (E to K) in residue 86 or (S to R/I) in residue 82 of ParC.

^d^Consensus sequence of the *pbp5*-R allele encoding the low affinity Pbp5-R.

^e^TC6 was not included in this analysis as it is a transconjugant of C68 and D344SRF, so therefore is not a unique genome.

Two groups have previously analyzed CRISPR-associated genes within *E. faecalis* and *E. faecium* genomes [32,61]. Partial CRISPR-like loci were previously described in E1071, E1679, and U0317; however, these loci were within a gene and were considered non-functional [32]. In addition, Palmer et al. identified CRISPR-cas predicted proteins in the Broad Institute strains Com12; 1,141,733; and 1,231,408 [61]. Similarly, we only found a CRISPR-cas locus in strain TX1330 (Additional file 9: Table S6) out of the 6 strains not previously studied (TX1330; TX16; TX0082; TX0133A; D344SRF; and C68). In summary, out of the 22 available genomes, only one of the HA-clade isolates contained CRISP-loci, namely the hybrid strain 1,231,408. The three other strains containing CRISPR-loci of the CA-clade (Com12; 1,141,733; and TX1330) all lacked antibiotic resistance determinants. Therefore, our data coincide with the previous observation that members of the recently emerged high-risk enterococcal lineages lack CRISPR-loci and the inverse relationship between the presence of a CRISPR-cas locus and acquired antibiotic resistance [61].

### Metabolic pathway

Metabolic pathways of *E. faecium* might have contributed to the recently increased incidence of *E. faecium* colonization and infection. To help understand *E. faecium* metabolism, the KEGG pathway (with EC number) and KAAS (with amino acid sequences) databases were used. Both databases predicted more than 100 pathways using TX16 genomic information. *E. faecium* exhibits major genomic differences in the genes involved in energy metabolism compared to that of other facultative anaerobic bacteria. However, like other species in the Lactobacillaceae order, genes for typical aerobic energy (ATP) generation through the TCA cycle and electron transport chain do not exist, i.e., genes encoding complex I (NADH dehydrogenase), II (succinate dehydrogenase,), III (cytochrome *bc*_1_ complex), and IV (cytochrome *c* oxidase).

When we compared the metabolic pathways of TX16 to those of *E. faecalis* V583 using the KEGG database*,* all 82 metabolic pathways of *E. faecalis* were also predicted in TX16. Indeed, more diverse metabolic activities were observed in TX16 (Additional file 10: Table S7 and Additional file 11: Table S8). Additional files 10: Table S7 and Additional files 11: Table S8 show lists of enzymes that only exist in *E. faecium* TX16 or *E. faecalis* V583 when KEGG enzymes from both strains were compared. Many of these enzymes were also described by van Schaik et al. who compared 7 European strains (also included in this study) to *E. faecalis* V583. They found 70 COGs present in their *E. faecium* genomes lacking in V583, whereas we found 176 predicted enzymes present in TX16 lacking in *E. faecalis* V583 according to KEGG analysis. Additionally, they found 140 COGs specific for *E. faecalis* V583, compared to the European strains, whereas we found only 112 enzymes specific to V583 when compared to TX16 according to KEGG analysis [32].

### Plasmids

Alignment of ORFs from the three plasmids of TX16 to the ORFs from the other 21 *E. faecium* genomes by BLASTP showed that all strains shared some ORFs that are similar to the ORFs of the three *E. faecium* TX16 plasmids (pDO1, pDO2 and pDO3), but none of them have more than 90% of the ORFs from any of the plasmids. It is likely that some strains may have similar but not identical plasmids as TX16, but identification of plasmids in other strains is difficult since those genomes are draft sequences. Alignment of ORFs of the three TX16 plasmids to 22 complete *E. faecium* plasmid sequences available in NCBI using TBLASTN with 90% identity and 50% match length cutoffs showed that pDO1 is most similar to plasmid pM7M2, a 19.5 kb plasmid which shared 27 ORFs of the 43 ORFs (62.8%) from pDO1, and that pDO2 is somewhat similar to plasmids pRUM and pS177 with 44.7% and 41.2% match to pDO2 ORFs respectively. TX16 plasmid pDO3 does not seem to be similar to any completely sequenced *E. faecium* plasmids but has similarity to the partially sequenced *E. faecium* large plasmid pLG1, Both pDO3 and pLG1plasmids harbor the hyaluronidase gene (*hyl*_*Efm*_), The *hyl*_*Efm*_ gene was also found in HA strains 1,230,933, 1,231,410, 1,231,502, C68, TC6 and U0317.

## Discussion

TX16 was the first *E. faecium* strain sequenced and has been used in various studies since [26,28,63,64]. The TX16 genome is characterized by numerous hyper variant loci and a large number of IS elements and transposons. Ortholog analysis as well as core and pan-genome analysis of TX16 and the other 21 sequenced strains revealed that *E. faecium* genomes are highly heterogeneous in gene content and possess a large number of dispensable genes. Similar to the findings by van Schaik et al. [32], pan and core genome analysis predict the pan genome to be open. Phylogenetic analysis using single-copy orthologs of the same length and gene content dissimilarity analysis in addition to recent studies [33,57] looking at core genes, SNPs and 16S rRNA, all indicate a large divergence between CA-clade isolates and HA-clade isolates. Furthermore, our previous analysis [33,57] and analyses within this study show that CC17 genogroup isolates cluster more closely together and further away from the CA-clade isolates than the other non-CC17 HA-clade isolates, indicating the CC17 genogroup is a more recently evolved genogroup.

Genomic island analysis by codon usage bias and composition variation showed that TX16 has 9 GIs, although TX16 also possesses a large number of hyper variant loci, suggesting that most of the genomic variable loci in TX16 were acquired through lateral gene transfer, possibly through mobile elements such as transposons. In general, strains in the HA clade harbored more transposons than the CA strains and certain IS elements such as IS*16.* These findings are consistent with a previous study using whole genome microarray [31].

Although IS16 presence has been proposed as an indicator of hospital-associated strains such as those apart of the CC17 genogroup [48], IS16 was not found in all HA-clade strains. Of note, however, all HA-clade strains contained the *pbp5*-R allele (except for 1,231,501 and D344SRF which is a spontaneous deletion mutant of *pbp5*) which may indicate that this is a reliable marker for hospital-associated isolates. Indeed, the *pbp5-R* allele is also found in animal and community isolates that are considered within the HA-clade, but not considered clinically associated [35,36]. The exception, 1,231,501 is interesting in that it is the HA-clade isolate from the blood of a hospitalized patient with no resistance genes, possibly supporting the concept that the genomic content of a strain, not just antibiotic resistance, adds to the survival in the hospital environment. In the 100 gene analysis by Galloway-Pena et al., it was found that 5 of the 92 genes of this strain studied grouped with the community clade, indicating it is a hybrid strain [33] as also reported in a recent study [34].

Capsular and other cell envelope polysaccharides of several gram-positive bacteria are known to have important roles in virulence and protective immunity [65-67]. Although the majority of studies on enterococcal surface polysaccharides have focused on *E. faecalis*, similar molecules have also been identified in *E. faecium* and suggested as targets for opsonic antibodies and as potential vaccine candidates [43,68], and also implicated in resistance of TX16 to phagocytosis in normal human serum [63]. Two such gene clusters, *cps* and *epa*, have been identified in *E. faecalis*[55,56,69,70]. Although a 7-9-gene *cps* region (*cpsC* to *cpsK*) was recently determined necessary for the production of an *E. faecalis* capsular polysaccharide [54] and shown to contribute to pathogenesis and evasion of the host innate immune response [67,69], TX16 only contains two homologs of the genes in this locus (*cpsA-cpsB)*[54]. In contrast, 15 of the 18 *E. faecalis epa* polysaccharide genes have homologs in TX16 and the other 21 *E. faecium* genomes, although their sequences vary between the two species. Therefore, it is likely that *E. faecalis* and *E. faecium* produce compositionally related, but not identical, Epa surface polysaccharides.

The hyper variable nature of the two polysaccharide loci found in TX16 raises the possibility that they are involved in biosynthesis of antigenically diverse surface polysaccharides which could help protect *E. faecium* against host immune responses. Similar to other gram-positive bacteria, various MSCRAMM-like cell wall anchored proteins have been previously identified in *E. faecium*; these include the collagen adhesin Acm and biofilm-associated Ebp pili, shown to be important for endocarditis and UTI in animal models [26,71], respectively, as well as two other collagen-binding MSCRAMMs, Scm and Fms18 (EcbA) [21,72]. Our comparison of 15 previously described MSCRAMM and pilus encoding genes of TX16 [17,18,21] with those of 21 *E. faecium* draft genomes found them to be common among these strains and the majority of them (12/15) to be enriched among HA clade strains or have a sequence variant mostly/exclusively carried by CA clade strains. Thus, these findings agree with previous hybridization results [14,16,17,22] and with the presence of two distinct subpopulations of *E. faecium*. Furthermore, one of these genes, *acm*, was previously found to be expressed more often by clinical versus non-clinical isolates, whereas a pseudogene was often found in isolates from the community [26,64]. Taken together, these data indicate a clear difference in the MSCRAMM and pilus gene profiles of the HA and CA clades, suggesting that these genes may have favored the emergence of HA-clade *E. faecium* in nosocomial infections.

When we combined our finding with previously published results, four of the 21 *E. faecium* genomes contain the CRISPR-cas locus. Three of these strains are within the CA clade and lack all antibiotic resistances analyzed in this study. One of the strains, 1,231,408, is a unique strain in which its genome is a hybrid of CA and HA genes. However, it does have 8 antibiotic resistance associated genes, showing there is not always an inverse relation between the number of antibiotic resistance determinants and the presence of CRISPR loci. More strains containing CRISPR-loci will need to be studied in order to determine if 1,231,408 is just an exception to the rule, or if the highly recombinant nature of *E. faecium* makes it different from *E. faecalis* with respect to the presence of CRISPR-loci in relation to antibiotic resistance determinants.

Overall, there seem to be some patterns that point to specific evolutionary events throughout *E. faecium’*s history as a species. First and foremost, there is a large ancestral split between the CA- and HA-clade strains which are separated by at least a 3–4% difference in their core genome [33]. The CA-clade isolates, except one, do not have either polysaccharide synthesis Locus 3 or 4 downstream of the *epa* region, antibiotic resistance genes, certain genomic islands, or IS elements. After the HA-clade diverged from CA-clade there was further evolution within the HA clade and some HA-clade strains studied here may represent phylogenetic transitional lineages (Figure 4B and C). Like the CA-clade strains, these transitional lineages are characterized by a lack of IS*16* (E1039; 1,231,501; and E1071) and have neither Locus 3 nor 4 (E1039; 1,231,501; E1071; E1636; E1679) in the *epa* extension. Although the data are limited, one scenario that could explain these observations is if Locus 1 replaced Locus 2 in a HA-clade ancestral strain, after the split from the CA clade, which later acquired IS*16* and then, subsequently, Locus 3 or 4 replaced Locus 1 in the *epa* extension region. Even if this is not the case, it seems clear that only strains further along in the phylogenetic trees, indicating a division within the HA-clade (Figure 4A and B), acquired IS*16* and the polysaccharide biosynthesis Loci 3 and 4. The exception is E980, a strain previously shown to have 8 of 92 genes from the HA-clade, which could have gained Locus 4 via recombination. Also of note, three of the four strains that have Locus 1 downstream of the *epa* locus lack the *ebp* genes, possibly suggesting there may have been some kind of gain and loss through homologous recombination.

**Figure 7:**
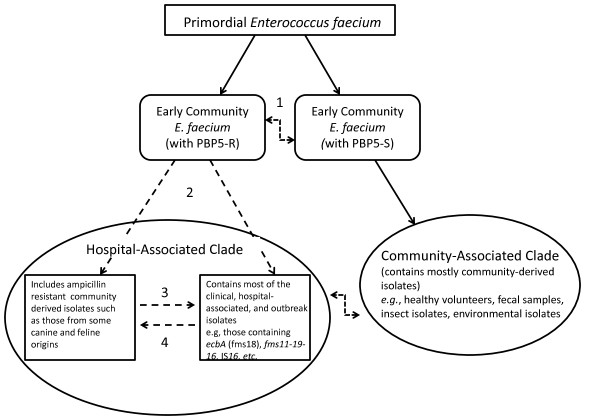
**The projected evolution of the two clades of*E. faecium*.** A figure addressing the possible scenarios which may have occurred in the evolution of *Enterococcus faecium* resulting in the HA-clade and CA-clade. Specifically, a primordial type of *Enterococcus faecium* split into early community isolates which had homologous core genomes with significant sequence differences (e.g., the *pbp5*-S or *pbp5*-R allele). These early community groups further segmented into a hospital-associated clade and the community clade. Scenario one depicts that these lineages could recombine with each other (represented by the bent dashed arrow) resulting in hybrid strains, scenario two depicts community and hospital ARE isolates splitting from the same ancestor, scenario three depicts ARE clones evolving from the animal reservoir, and scenario four depicts animal ARE isolates representing descendants of hospital ARE transferred from humans to their pets.

Figure 7 shows the projected scenarios for the evolution of the two clades of *E. faecium* as can be envisioned using our data as well as other previous publications [31,33,34,57]. The hypothesis is that there was a primordial type of *E. faecium* which split many millinea ago and evolved into two early community groups which had homologous genes e.g. the *pbp5*-S or *pbp5*-R alleles, the latter representing community sources of ARE (ampicillin resistant *E. faecium*). These lineages could recombine with each other resulting in hybrid strains (i.e. 1,231,408 and 1,231,501) (scenario 1). The divergence between the two community groups eventually reached a core genomic difference of approximately 3–4%, creating a HA clade, which includes both ampicillin- resistant, community-based isolates, such as those from some canine and feline origins, as well as most of the clinical-, hospital- and outbreak- associated isolates and a CA clade, which consists mostly of community derived isolates. Most likely, community and hospital ARE isolates split from the same ancestor, as represented by scenario two. However, it is also possible that ARE clones evolved from the animal reservoir (scenario 3), or that animal ARE isolates represent evolutionary descendants of hospital ARE transferred from humans to their pets (scenario 4).

## Conclusions

In conclusion, the completion of the TX16 genome has provided insight into the intricate genomic features of *E. faecium*, and will surely serve as an important reference for those studying *E. faecium* genomics in the future. By studying TX16, an endocarditis isolate belonging to CC17, and comparing the TX16 genome to the other 21 draft genomes, we have been able to confirm the high genomic plasticity of this organism. The HA-clade isolates contain a number of unique IS elements, transposons, phages, plasmids, genomic islands, and inherent and acquired antibiotic resistance determinants, most likely contributing to the emergence of this organism in the hospital environment that has occurred in the last 30 years.

## Methods

### Bacterial strains and DNA sequencing

The *E. faecium* strain TX16 (DO) was isolated from the blood of a patient with endocarditis [63] and *E. faecium* TX1330 was isolated from the stool of a healthy volunteer [18,73]. Routine bacterial growth was on BHI agar or broth, and genomic DNA was isolated from overnight culture using the method previously described [74].

Both *E. faecium* TX16 and TX1330 were sequenced, assembled and annotated as part of the reference genome project in the Human Microbiome Project (HMP). *E. faecium* TX16 was initially sequenced by traditional Sanger sequencing technology to 15.6x read sequence coverage, and subsequently by 454 GS20 technology to 11x read sequence coverage of fragment reads, 7.5x sequence coverage of 2 kb insert paired end reads, and by 454 FLX platform to 73x sequence coverage of 8 kb insert paired-end reads. Both Sanger and 454 reads were assembled using 454 Newbler assembler. The gaps between contigs in scaffolds were closed using the unassembled mate paired reads or by PCR sequencing of the DNA products amplified from the primers flanking the gaps. The assembly and gap closure of TX16 was difficult due to large number of repetitive sequences in the genome. The addition of the large insert 8 kb library with deep clone coverage was able to facilitate the assembly and scaffolding to generate high quality contigs and scaffolds in the *de novo* assembly. *E. faecium* strain TX1330 was sequenced by 454 GS20 technology to 6x sequence coverage for fragment reads and by 454 FLX to 69.8x sequence coverage for paired end reads, respectively. TX1330 was also assembled using 454 Newbler assembler.

Plasmids were identified by circularization of DNA sequences by paired end reads, and were also experimentally verified by PFGE analysis of SmaI and ApaI digested genomic DNA followed by hybridization with PCR-generated probes complementary to 5′ and 3′ ends of plasmid contigs. PFGE hybridization profiles were then compared to identify neighboring plasmid contigs.

The gene prediction for both *E. faecium* TX16 and TX1330 was accomplished by Glimmer 3 [75] and GeneMark [76]. tRNAScan [77] was used for tRNA prediction, RNAmmer [78] for rRNA prediction, and RFAM/infernal for other non-coding RNA genes [79]. Manual annotation was facilitated by Genboree genome browser (http://www.genboree.org). Conserved protein domains were searched using Pfam [80], COG [81], and InterProScan [82]. Other tools such as PsortB [83,84], ExPASy ENZYME [85], and the Transport Classification Database [86] were also used to facilitate the annotation. For manual annotation, each entry was annotated by two annotators independently and the differences were reconciliated at the end of the annotation.

Genomic sequences and annotations for 20 other draft *E. faecium* strains, including 1,141,733; 1,230,933; 1,231,408; 1,231,410; 1,231,501; 1,231,502; C68; Com12; Com15; D344SRF; E1039; E1071; E1162; E1636; E1679; E980; TC6; TX82; TX0133A; U0317, were obtained from NCBI. A complete list of the strains and their clinical sources is provided in Table 2.

### Genome characterization

DNA and protein sequence alignments were performed using BLASTN and BLASTP [87], respectively, unless otherwise stated. Prophage loci were identified using both Prophinder program [47] and Prophage Finder [46]. Prophinder uses BLASTP to search phage proteins in the ACLAME database while Prophage Finder uses BLASTX to search input DNA sequence to an NCBI database of phage genomes. Possible prophage loci were also reviewed manually. IslandViewer [52] server was used to analyze possible genomic islands on the chromosome. IslandViewer integrated sequence composition based genomic island prediction programs including IslandPath-DIMOB [50] and SIGI-HMM [51] as well as comparative genome based program IslandPick [53] for genomic island prediction. Genes and DNA sequence in the identified genomic regions were used to perform the BLAST search against the other 21 *E. faecium* genomes to investigate the presence or absence of clade specific genomic islands. Repeat sequences were identified by RepeatScout [88]. Circular genome maps were generated using the CGView program [89].

BLASTN and BLASTX as well as ISfinder server [90] were used to identify IS sequences and transposons in the TX16 chromosome and plasmids. Genomic regions with homology to IS and transposon sequences from both BLAST analyses were verified with the gene annotation of TX16. Both BLAST searches identified many small regions as a part of IS elements and transposons. Regions with shorter than 60% match length to reference sequences were excluded from further analysis. Identified genes/regions by analyses above were also used to perform the BLAST search against the other 21 *E. faecium* genomes to investigate whether there are clade specific presences or absences.

Chromosomal DNA sequences of TX16 and Aus0004 were aligned using Mauve 2.3.1 and performed a comparative genomic analysis [91,92]. Junction sites of 5 locally collinear blocks (LCB) of Mauve alignment were further investigated with genome annotation to identify possible reasons of two inversions and DNA insertions.

Six genomes that had yet to be studied for CRISPR-loci were analyzed for CRISPR loci (TX1330, TX16, TX82, TX0133A, D344SRF, and C68). We searched for CRISPR loci in the six genomes by performing BLAST using the sequences from the ORFs previously described for CRISPR-loci in *E. faecium* EFVG_01551 to EFVG_01555 [61], as well as using CRISPRfinder (http://crispr.u-psud.fr/Server/CRISPRfinder.php) and the CRT program [93] to detect prophage CRISPR palindromic repeats in TX16.

Conserved gene orders between *E. faecium* TX16, *E. faecalis* V583 [41] and *E. faecalis* OG1RF genomes [40] were identified using BLASTP with E value of 1e-3 and DAGchainer with default parameters [39].

The extrapolation of core-genome and pan-genome was performed as described previously [94,95]. ORF protein sequences were aligned using BLASTP, and a gene pair was considered present in two strains if the alignment covered at least 50% length of the shorter gene with at least 70% sequence identity. Due to the large number of possible combinations of 22 strains, only 100 permutations were performed for each *n*th genome.

Metabolic pathways of the TX16 genome were analyzed with enzyme commission (EC) numbers as well as with the predicted amino acid sequences of all TX16 ORFs. 528 unique EC numbers of TX16 genome are analyzed at the KEGG server (http://www.genome.jp/kegg/pathway.html) to predict the metabolic pathway. Also, KEGG automatic annotation server (http://www.genome.ad.jp/kaas-bin/kaas_main) was used for functional annotation of the TX16 ORFs. Metabolic pathways and enzymes identified from TX16 were compared to that of *E. faecalis* V583 (KEGG genome T00123) in KEGG pathway database.

### Ortholog, phylogenetic and multi-locus sequence typing (MLST) analysis

Protein ortholog groups of *E. faecium* genomes were identified using OrthoMCL program [96] using BLASTP E value of 1e-5 and default MCL inflation parameter of 1.5 with 80% sequence identity and 60% match length cutoffs. The match length percentage was set relatively low because all the genomes except TX16 are draft sequences. The dissimilarity in gene content among the *E. faecium* genomes was calculated using Jaccard distance (1- Jaccard coefficient) as described previously [97], and the Jaccard distance matrix was used for hierarchical clustering using the unweighted pair group method with arithmetic mean (UPGMA). Single-copy orthologs with the same length in all strains were chosen for phylogenetic analysis after removing genes that may have undergone recombination detected by PHI program [98]. Multiple sequence alignments were performed by MAFFT program [99] and the topology of the phylogenetic tree was inferred by maximum-likelihood algorithm using PhyML [100] with bootstrap value of 100. 16S rRNA phylogenetic analysis was performed in another manuscript [33]. iTOL program [101] was used for phylogenetic tree visualization.

The *in silico* multi-locus sequence types were determined either by extracting the allele types of *adkatpAddlgdhgydpstS*, and *purK* from the genomic sequence, or using the allele numbers previously obtained through experimentation [57]. The allele numbers and sequence types were used to construct an UPGMA dendogram using S.T.A.R.T.2 software (http://pubmlst.org/).

### Identification of putative virulence-associated genes and antibiotic resistance determinants

Putative virulence genes were identified by BLASTP of *E. faecium* ORF protein sequences to the enterococcal virulence factors in the Virulence Factors Database (VFDB) [59], and hits were manually inspected.

To identify antibiotic resistance genes, BLASTN was performed using the nucleotide sequences of 13 antibiotic resistance genes including *cat* (chloramphenicol O-acetyltransferase) using the EfmE1071_2206 sequence which is an ortholog to the *cat* gene found on the *E. faecium* plasmid pRUM [102]*ermA* (rRNA adenine N-6-methyltransferase) using the EfmE1679_0214 sequence and located on Tn554 [103]; *ermB* (rRNA adenine N-6-methyltransferase) using the EfmE1071_2296 sequence, an ortholog to the *ermB* gene found on the *E. faecalis* plasmids pRE25 and pSL1[104]; *aad6* (aminoglycoside 6-adenylyltransferase) using the EfmE1071_1021 sequence an ortholog to the genes found on the *E. faecalis* plasmid pEF418 (Genbank:AF408195); *aad9* (streptomycin 3″-adenylyltransferase) using EfmE1679_0213 sequence and located on Tn*554*[103]; *aadE* (aminoglycoside 6-adenylyltransferase) using EfmU0317_2169 sequence an ortholog to the gene found on the *E. faecalis* plasmid pRE25 [104]; *aacA-aphD* (bifunctional aminoglycoside modifying enzyme) using the EfmU0317_2161 sequence; *tetL* using the EfmE1071_1017 sequence [105]; *tetM* using the EfmE1162_0404 sequence [105]; *vanA* using the EfmE1071_0104 to EfmE1071_0110 sequence which is identical to the *vanA* gene cluster found on Tn*1546*[106]; *gyrA* using EfmE1679_2520 to determine amino acid changes of E87K/G or S83R/Y/I [107]; *parC* using EfmE1679_0369 to determine amino acid changes of E86K or S82R/I [107]; and *pbp5* (GenBank accession no. ZP_00603984) to search for the low-affinity *pbp5* consensus sequence [57,108].

### Database submission

The genome sequences, plasmid sequences, and the gene annotation of *E. faecium* TX16, pDO1, pDO2, and pDO3, were submitted to GenBank with the accession numbers of CP003583, CP003584, CP003585, and CP003586 respectively. The draft sequence of TX1330 was submitted to GenBank with the accession number ACHL01000000.

## Authors’ contributions

XQ carried out the annotations, genome characterization, genome analyses, closure of the genome and drafting of the manuscript. JGP carried out annotations, phylogenetic, antibiotic resistance, and CRISPR analyses, and writing /submission of the manuscript. JS carried out the annotations, genome, MSCRAMM, virulence genes, and polysaccharide biosynthesis analyses, and drafting of the manuscript. JHR carried out metabolic pathway, genomic island, and mobile element analyses and drafting of the manuscript. The rest of the authors contributed though annotating or sequencing of the genome. GMW and BEM contributed their study design, overseeing the study, and editing of the manuscript. All authors read and approved the final manuscript.

## Supplementary Material

Additional file 1 **Figure S1.Gene order synteny of*E. faecium*TX16 compared to*E. faecalis*V583.** A figure ploting the synteny blocks between TX16 and V583 with the coordinates of each genome. Click here for file

Additional file 2 **Figure S2.Genome alignment of TX16 and Aus0004.** A figure comparing the two closed *E. faecium* genomes sequences available using Mauve genome alignment analysis. Click here for file

Additional file 3 **Table S1.Hospital-associated clade unique genes.** A table listing the genes and their corresponding ORF in TX16 that are unique to the hospital clade and how many of the HA clade strains the gene is present in. Click here for file

Additional file 4 **Table S2.Prophage loci and genes on*E. faecium*TX16 genome.** A table listing the two prophage loci, the predicted gene products within these two loci, and the corresponding ORFs in TX16. Click here for file

Additional file 5 **Table S3.Mobile elements in the*E. faecium*TX16 genome.** A table listing all of the predicted mobile elements and their corresponding locus tags in TX16. Click here for file

Additional file 6 **Table S4.*E. faecium*TX16 genomic islands and genes.** A table listing the nine genomic islands, the genes and predicted products within those islands, and the corresponding ORFs and coordinates within TX16. Click here for file

Additional file 7 **Figure S3.ORF composition of the downstream extension of the*epa*gene cluster in the 22*E. faecium*genomes (HMPREF0351_10908 - HMPREF0351_10923 in TX16).** A figure depicting the predicted polysaccharide-encoding gene clusters found in the *E. faecium* genomes. Click here for file

Additional file 8 **Table S5.Presence of genes encoding MSCRAMMs and pilins among 21*E. faecium*genomes.** A table listing the different MSCRAMM and pilin variants present in each of the 22 genomes. Click here for file

Additional file 9 **Table S6.Summary of CRISPRs found in*E. faecium*sequenced strains.** A table listing in what strains CRISPRs were found, the locus tag, and the functional assignment. Click here for file

Additional file 10 **Table S7.Specific enzymes present in TX16 but not in*E. faecalis*V583.** A table listing enzymes, KEGG information, and locus tags specific to TX16. Click here for file

Additional file 11 **Table S8.Specific enzymes present in*E. faecalis* V583 but not in TX16.** A table listing the enzymes and locus tags specific to V583. Click here for file
