# Supplementary material for: Complete genome sequence of Enterococcus faecium strain TX16 and comparative genomic analysis of Enterococcus faecium genomes
Source: BMC Microbiol. 2012 Jul 7;12:135. doi: 10.1186/1471-2180-12-135 (PMC3433357; doi:10.1186/1471-2180-12-135)
Supplement: Additional file 1 — Figure S1.Gene order synteny ofE. faeciumTX16 compared toE. faecalisV583. A figure ploting the synteny blocks between TX16 and V583 with the coordinates of each genome. [file 1471-2180-12-135-S1.ppt]

## Slide 1
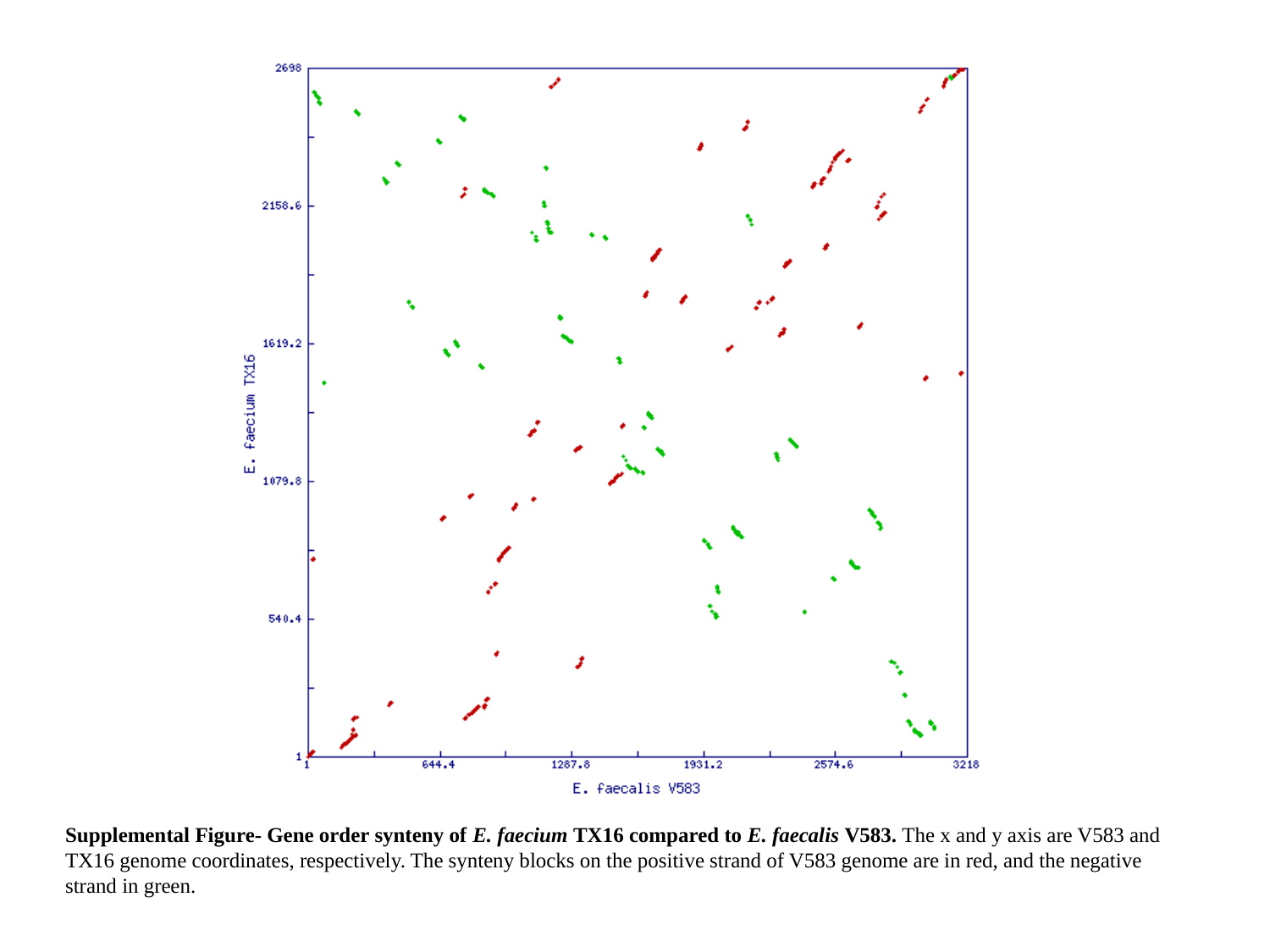

Supplemental Figure- Gene order synteny of E. faecium TX16 compared to E. faecalis V583. The x and y axis are V583 and TX16 genome coordinates, respectively. The synteny blocks on the positive strand of V583 genome are in red, and the negative strand in green.
