# Supplementary material for: Complete genome sequence of Enterococcus faecium strain TX16 and comparative genomic analysis of Enterococcus faecium genomes
Source: BMC Microbiol. 2012 Jul 7;12:135. doi: 10.1186/1471-2180-12-135 (PMC3433357; doi:10.1186/1471-2180-12-135)
Supplement: Additional file 2 — Figure S2.Genome alignment of TX16 and Aus0004. A figure comparing the two closed E. faecium genomes sequences available using Mauve genome alignment analysis. [file 1471-2180-12-135-S2.pptx]

## Slide 1
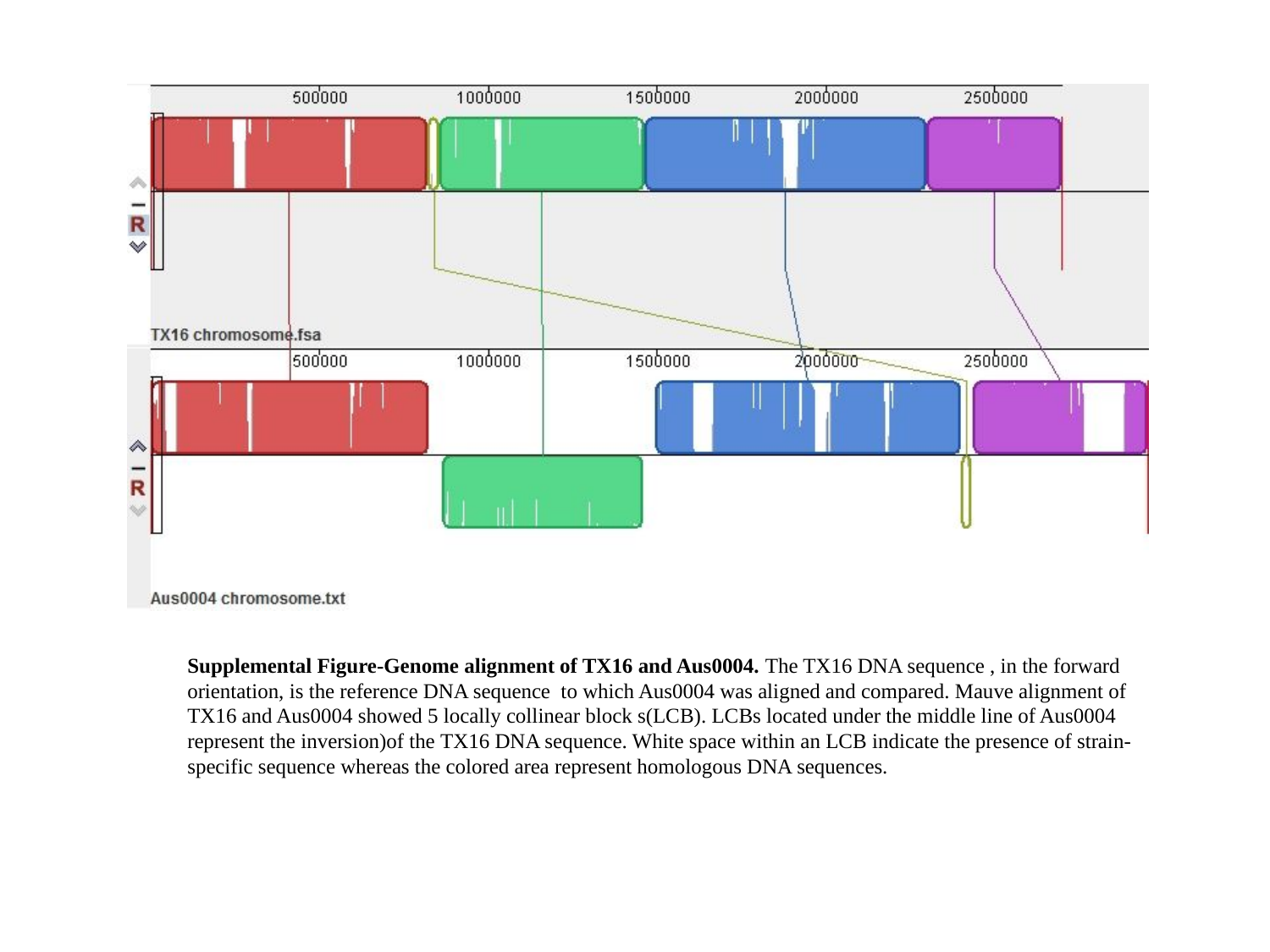

Supplemental Figure-Genome alignment of TX16 and Aus0004. The TX16 DNA sequence , in the forward orientation, is the reference DNA sequence to which Aus0004 was aligned and compared. Mauve alignment of TX16 and Aus0004 showed 5 locally collinear block s(LCB). LCBs located under the middle line of Aus0004 represent the inversion)of the TX16 DNA sequence. White space within an LCB indicate the presence of strain-specific sequence whereas the colored area represent homologous DNA sequences.
