# Supplementary material for: Complete genome sequence of Enterococcus faecium strain TX16 and comparative genomic analysis of Enterococcus faecium genomes
Source: BMC Microbiol. 2012 Jul 7;12:135. doi: 10.1186/1471-2180-12-135 (PMC3433357; doi:10.1186/1471-2180-12-135)
Supplement: Additional file 3 — Table S1.Hospital-associated clade unique genes. A table listing the genes and their corresponding ORF in TX16 that are unique to the hospital clade and how many of the HA clade strains the gene is present in. [file 1471-2180-12-135-S3.doc]

**Supplemental Table-Hospital-associated** clade unique genes

| **TX16 gene ID** | **Present in numbers of isolates in the HA clade** | **Start** | **End** | **Strand** | **Predicted**  **Gene Product** | **Gene product name** |
| --- | --- | --- | --- | --- | --- | --- |
| HMPREF0351_10025 | 10/16 | 30165 | 30503 | + | . | conserved hypothetical protein |
| HMPREF0351_10026 | 10/16 | 30597 | 30827 | + | . | conserved hypothetical protein |
| HMPREF0351_10027 | 13/16 | 31227 | 31598 | + | . | conserved hypothetical protein |
| HMPREF0351_10028 | 13/16 | 31872 | 32564 | - | . | haloacid dehalogenase (HAD) superfamily hydrolase |
| HMPREF0351_10029 | 13/16 | 32567 | 33352 | - | *lacR* | lactose PTS family porter repressor |
| HMPREF0351_10030 | 13/16 | 33589 | 34989 | + | *lacG* | 6-phospho-beta-galactosidase |
| HMPREF0351_10031 | 13/16 | 34999 | 35331 | + | *lacF* | PTS family lactose-N,N'-diacetylchitobiose-beta-glucoside (lac) porter component IIA |
| HMPREF0351_10032 | 13/16 | 35344 | 37017 | + | *lacE* | PTS family lactose-N,N'-diacetylchitobiose-beta-glucoside (lac) porter component IIBC |
| HMPREF0351_10033 | 13/16 | 37031 | 38662 | + | *galA* | arabinogalactan endo-1,4-beta-galactosidase |
| HMPREF0351_10148 | 16/16 | 145071 | 145202 | + | . | hypothetical protein |
| HMPREF0351_10170 | 12/16 | 168638 | 168976 | - | . | DNA integrase |
| HMPREF0351_10171 | 12/16 | 169044 | 169445 | - | . | conserved hypothetical protein |
| HMPREF0351_10172 | 13/16 | 169442 | 169978 | - | . | IS3/IS911 family transposase |
| HMPREF0351_10187 | 16/16 | 183487 | 183618 | - | . | hypothetical protein |
| HMPREF0351_10254 | 2/16 | 247996 | 248706 | + | . | cell wall protein |
| HMPREF0351_10255 | 2/16 | 249134 | 249526 | + | . | conserved hypothetical protein |
| HMPREF0351_10263 | 2/16 | 256739 | 257332 | + | . | ParB family nuclease |
| HMPREF0351_10267 | 8/16 | 258878 | 259018 | + | . | conserved hypothetical protein |
| HMPREF0351_10268 | 8/16 | 259045 | 259344 | + | . | 4-phosphopantetheinyl transferase |
| HMPREF0351_10269 | 7/16 | 259372 | 259506 | + | . | hypothetical protein |
| HMPREF0351_10270 | 7/16 | 259518 | 260063 | + | . | conserved hypothetical protein |
| HMPREF0351_10273 | 5/16 | 261480 | 262487 | + | . | conserved hypothetical protein |
| HMPREF0351_10279 | 3/16 | 265704 | 267077 | + | *ccrA* | cassette chromosome recombinase A |
| HMPREF0351_10280 | 3/16 | 267081 | 268718 | + | *ccrB* | cassette chromosome recombinase B |
| HMPREF0351_10281 | 3/16 | 268961 | 270049 | + | *.* | conserved hypothetical protein |
| HMPREF0351_10286 | 2/16 | 272199 | 274856 | + | *.* | NtrC family transcriptional regulator |
| HMPREF0351_10287 | 2/16 | 274928 | 275404 | + | *.* | PTS family mannose/fructose/sorbose porter component IIB |
| HMPREF0351_10288 | 2/16 | 275418 | 276206 | + | *.* | PTS family mannose/fructose/sorbose porter component IIC |
| HMPREF0351_10289 | 2/16 | 276199 | 277005 | + | *.* | PTS family mannose/fructose/sorbose porter component IID |
| HMPREF0351_10290 | 2/16 | 277024 | 277419 | + | *.* | PTS family mannose/fructose/sorbose porter component IIA |
| HMPREF0351_10291 | 2/16 | 277421 | 278542 | + | *.* | conserved hypothetical protein |
| HMPREF0351_10292 | 2/16 | 278562 | 279431 | + | *cscK* | fructokinase |
| HMPREF0351_10293 | 14/16 | 279632 | 280141 | + | . | conserved hypothetical protein |
| HMPREF0351_10304 | 6/16 | 288056 | 288214 | + | . | hypothetical protein |
| HMPREF0351_10305 | 8/16 | 288236 | 288502 | + | . | conserved hypothetical protein |
| HMPREF0351_10306 | 8/16 | 288529 | 288828 | + | . | conserved hypothetical protein |
| HMPREF0351_10307 | 7/16 | 288856 | 288990 | + | . | hypothetical protein |
| HMPREF0351_10308 | 7/16 | 289002 | 289553 | + | . | conserved hypothetical protein |
| HMPREF0351_10309 | 7/16 | 289699 | 289992 | + | . | conserved hypothetical protein |
| HMPREF0351_10310 | 7/16 | 289985 | 290281 | + | . | peptidase |
| HMPREF0351_10311 | 7/16 | 290326 | 291354 | + | . | conserved hypothetical protein |
| HMPREF0351_10314 | 10/16 | 293413 | 294708 | + | . | IS204/IS1001/IS1096/IS1165 family transposase |
| HMPREF0351_10315 | 12/16 | 294755 | 297466 | - | . | sulphatase |
| HMPREF0351_10316 | 13/16 | 297453 | 297779 | - | . | alkaline phosphatase |
| HMPREF0351_10321 | 12/16 | 302715 | 303236 | + | . | conserved hypothetical protein |
| HMPREF0351_10334 | 15/16 | 316053 | 316517 | + | . | acetyltransferase |
| HMPREF0351_10337 | 11/16 | 318408 | 318737 | - | . | conserved hypothetical protein |
| HMPREF0351_10363 | 12/16 | 345792 | 346598 | - | . | DNA integrase |
| HMPREF0351_10364 | 13/16 | 346595 | 347131 | - | . | transposase IS3/IS911 |
| HMPREF0351_10377 | 14/16 | 357832 | 359328 | + | *bglH* | beta-glucosidase |
| HMPREF0351_10378 | 14/16 | 359378 | 361153 | + | *.* | BglG family transcriptional antiterminator |
| HMPREF0351_10379 | 14/16 | 361211 | 363106 | + | *bglP* | PTS family glucose/glucoside (glc) porter component IIABC |
| HMPREF0351_10380 | 14/16 | 363103 | 364560 | + | *bglA* | 6-phospho-beta-glucosidase |
| HMPREF0351_10405 | 16/16 | 389028 | 389693 | + | *.* | conserved hypothetical protein |
| HMPREF0351_10475 | 16/16 | 472585 | 472833 | + | *.* | cysteinyl-tRNA synthetase |
| HMPREF0351_10521 | 10/16 | 516156 | 516341 | + | *.* | hypothetical protein |
| HMPREF0351_10522 | 10/16 | 516538 | 516705 | + | *.* | hypothetical protein |
| HMPREF0351_10523 | 10/16 | 516828 | 517040 | + | *.* | hypothetical protein |
| HMPREF0351_10524 | 10/16 | 517367 | 517480 | + | *.* | IS200 family transposase |
| HMPREF0351_10534 | 16/16 | 529145 | 530842 | + | *fms13* | LPXTG family cell surface protein Fms13 |
| HMPREF0351_10581 | 4/16 | 578672 | 579469 | - | . | transcriptional regulator |
| HMPREF0351_10583 | 6/16 | 580235 | 581110 | + | . | conserved hypothetical protein |
| HMPREF0351_10591 | 5/16 | 587283 | 588134 | + | . | conserved hypothetical protein |
| HMPREF0351_10592 | 5/16 | 588167 | 589603 | + | . | conserved hypothetical protein |
| HMPREF0351_10593 | 5/16 | 589605 | 590090 | + | . | conserved hypothetical protein |
| HMPREF0351_10594 | 13/16 | 590718 | 591899 | - | . | phage integrase |
| HMPREF0351_10595 | 13/16 | 591899 | 592633 | - | . | transcriptional regulator |
| HMPREF0351_10596 | 13/16 | 592865 | 593140 | + | . | excisionase/Xis |
| HMPREF0351_10597 | 13/16 | 593238 | 595235 | + | . | conserved hypothetical protein |
| HMPREF0351_10598 | 13/16 | 595311 | 595646 | - | . | transcriptional regulator |
| HMPREF0351_10599 | 13/16 | 595775 | 595990 | + | . | transcriptional regulator |
| HMPREF0351_10600 | 13/16 | 596006 | 596392 | + | . | conserved hypothetical protein |
| HMPREF0351_10601 | 13/16 | 596408 | 596755 | + | . | conserved hypothetical protein |
| HMPREF0351_10602 | 9/16 | 596909 | 597634 | + | . | botulinum neurotoxin type C1 |
| HMPREF0351_10604 | 8/16 | 599729 | 600973 | + | *hsdS* | type I site-specific deoxyribonuclease specificity subunit |
| HMPREF0351_10606 | 10/16 | 604854 | 606317 | - | . | transposase |
| HMPREF0351_10701 | 14/16 | 702098 | 702262 | + | . | hypothetical protein |
| HMPREF0351_10702 | 14/16 | 702507 | 705203 | + | . | transcriptional antiterminator |
| HMPREF0351_10703 | 14/16 | 705280 | 705768 | + | . | PTS family fructose/mannitol (fru) porter component IIA |
| HMPREF0351_10704 | 14/16 | 705780 | 706274 | + | . | PTS family fructose/mannitol (fru) porter component IIB |
| HMPREF0351_10705 | 14/16 | 706295 | 707104 | + | . | PTS family fructose/mannitol (fru) porter component IIC |
| HMPREF0351_10706 | 14/16 | 707101 | 707922 | + | . | PTS family fructose/mannitol (fru) porter component IID |
| HMPREF0351_10707 | 13/16 | 707973 | 710072 | + | . | glycoside hydrolase |
| HMPREF0351_10708 | 14/16 | 710069 | 711187 | + | . | conserved hypothetical protein |
| HMPREF0351_10709 | 14/16 | 711187 | 712104 | + | *cscK2* | fructokinase |
| HMPREF0351_10710 | 13/16 | 712905 | 713096 | + | *.* | hypothetical protein |
| HMPREF0351_10711 | 14/16 | 713470 | 714282 | - | *.* | transcriptional regulator |
| HMPREF0351_10712 | 14/16 | 714601 | 715677 | + | *.* | replication initiation factor superfamily protein |
| HMPREF0351_10713 | 13/16 | 715819 | 716022 | + | *.* | conserved hypothetical protein |
| HMPREF0351_10714 | 14/16 | 716082 | 717284 | + | *.* | bacteriophage integrase |
| HMPREF0351_10725 | 16/16 | 729699 | 730148 | + | *.* | PTS family fructose/mannitol (fru) porter component IIA |
| HMPREF0351_10726 | 16/16 | 730205 | 731623 | + | *fruA2* | PTS family fructose/mannitol (fru) porter component IIBC |
| HMPREF0351_10727 | 16/16 | 731623 | 732483 | + | *fba3* | fructose-bisphosphate aldolase |
| HMPREF0351_10728 | 16/16 | 732657 | 733430 | + | . | DeoR family transcriptional regulator |
| HMPREF0351_10820 | 4/16 | 822515 | 823099 | - | . | prophage superinfection immunity protein |
| HMPREF0351_10823 | 4/16 | 824289 | 824429 | + | . | conserved hypothetical protein |
| HMPREF0351_10824 | 4/16 | 824560 | 824730 | + | . | hypothetical protein |
| HMPREF0351_10825 | 4/16 | 824727 | 824984 | + | . | phosphomannomutase/phosphoglucomutase-like protein |
| HMPREF0351_10826 | 4/16 | 824955 | 825263 | - | . | conserved hypothetical protein |
| HMPREF0351_10831 | 7/16 | 826868 | 827338 | - | . | conserved hypothetical protein |
| HMPREF0351_10835 | 8/16 | 828635 | 829306 | + | . | conserved hypothetical protein |
| HMPREF0351_10844 | 9/16 | 832298 | 832516 | + | . | cobalamin biosynthesis protein CobD |
| HMPREF0351_10845 | 7/16 | 832513 | 832932 | + | . | YopX protein |
| HMPREF0351_10846 | 6/16 | 832929 | 833486 | + | . | conserved hypothetical protein |
| HMPREF0351_10851 | 5/16 | 835456 | 835662 | + | . | hypothetical protein |
| HMPREF0351_10852 | 10/16 | 835858 | 836025 | + | . | thymidylate synthase |
| HMPREF0351_10853 | 10/16 | 836051 | 836395 | + | *mcrA* | HNH endonuclease |
| HMPREF0351_10854 | 10/16 | 836376 | 836681 | + | *.* | bacteriophage protein |
| HMPREF0351_10855 | 10/16 | 836784 | 837098 | + | *terS* | bacteriophage terminase small subunit |
| HMPREF0351_10856 | 10/16 | 837076 | 838770 | + | *terL* | bacreriophage terminase large subunit |
| HMPREF0351_10857 | 10/16 | 838772 | 839968 | + | *.* | bacteriophage portal protein |
| HMPREF0351_10858 | 10/16 | 839931 | 840617 | + | *clpP* | S14 family endopeptidase ClpP |
| HMPREF0351_10859 | 10/16 | 840617 | 841777 | + | *cps* | bacteriophage major capsid protein B |
| HMPREF0351_10860 | 10/16 | 841787 | 842662 | + | *.* | bacteriophage protein |
| HMPREF0351_10861 | 10/16 | 842659 | 842970 | + | *.* | bacteriophage protein |
| HMPREF0351_10862 | 10/16 | 842960 | 843313 | + | *.* | bacteriophage protein |
| HMPREF0351_10863 | 10/16 | 843303 | 843704 | + | *.* | bacteriophage protein |
| HMPREF0351_10864 | 10/16 | 843697 | 844101 | + | *.* | bacteriophage protein |
| HMPREF0351_10865 | 10/16 | 844113 | 844721 | + | *tsh* | bacteriophage major tail protein |
| HMPREF0351_10866 | 10/16 | 844740 | 845102 | + | *.* | bactriophage protein |
| HMPREF0351_10867 | 10/16 | 845147 | 845287 | + | *.* | hypothetical protein |
| HMPREF0351_10868 | 9/16 | 845304 | 848735 | + | *.* | conserved bacteriopahge tail protein |
| HMPREF0351_10869 | 10/16 | 848786 | 849523 | + | *.* | conserved bacteriophage protein |
| HMPREF0351_10870 | 10/16 | 849533 | 851824 | + | *.* | bactriophage minor structural protein |
| HMPREF0351_10871 | 5/16 | 851848 | 853974 | + | *.* | bactriophage minor structural protein |
| HMPREF0351_10876 | 10/16 | 855050 | 855274 | + | *hol* | bacteriophage holin |
| HMPREF0351_10878 | 12/16 | 857236 | 857526 | + | . | IS3/IS911 transposase |
| HMPREF0351_10879 | 11/16 | 857562 | 858398 | + | . | integrase |
| HMPREF0351_10880 | 13/16 | 858483 | 858977 | - | . | transposase |
| HMPREF0351_10881 | 7/16 | 859321 | 859728 | + | . | conserved hypothetical protein |
| HMPREF0351_10882 | 7/16 | 859742 | 860143 | + | . | conserved hypothetical protein |
| HMPREF0351_10883 | 7/16 | 860145 | 860516 | + | . | conserved hypothetical protein |
| HMPREF0351_10885 | 16/16 | 861103 | 861303 | - | . | bacteriophage integrase |
| HMPREF0351_10924 | 11/16 | 901564 | 902010 | - | . | conserved hypothetical protein |
| HMPREF0351_10925 | 12/16 | 902007 | 902753 | - | . | transposase |
| HMPREF0351_10926 | 13/16 | 902899 | 903093 | + | . | conserved hypothetical protein |
| HMPREF0351_10927 | 13/16 | 903083 | 903448 | + | . | transposon protein |
| HMPREF0351_10928 | 13/16 | 903537 | 905084 | + | . | transposase IS66 |
| HMPREF0351_10995 | 10/16 | 971959 | 973254 | - | . | transposase |
| HMPREF0351_11009 | 16/16 | 997521 | 998396 | + | . | transcriptional antiterminator |
| HMPREF0351_11010 | 15/16 | 998500 | 999264 | + | . | YdjC family protein |
| HMPREF0351_11011 | 16/16 | 999251 | 1000693 | + | *nagE* | PTS family porter, N-acetylglucosamine-specific component IIABC |
| HMPREF0351_11012 | 16/16 | 1000704 | 1002035 | + | *malH* | maltose-6'-phosphate glucosidase |
| HMPREF0351_11013 | 16/16 | 1002048 | 1002506 | + | *ptsG2* | PTS family porter glucose-specific component IIABC |
| HMPREF0351_11016 | 16/16 | 1004514 | 1004663 | + | *.* | hypothetical protein |
| HMPREF0351_11017 | 16/16 | 1004686 | 1004853 | + | *bsr* | cytidine deaminase family enzyme blasticidin S deaminase |
| HMPREF0351_11018 | 16/16 | 1004901 | 1005089 | + | . | hypothetical protein |
| HMPREF0351_11019 | 16/16 | 1005385 | 1006620 | - | . | glycosyltransferase |
| HMPREF0351_11035 | 13/16 | 1020193 | 1021443 | - | . | IS116/IS110/IS902 family transposase |
| HMPREF0351_11074 | 15/16 | 1061281 | 1061448 | + | . | hypothetical protein |
| HMPREF0351_11079 | 10/16 | 1063888 | 1065183 | + | . | transposase |
| HMPREF0351_11098 | 16/16 | 1086585 | 1086716 | - | . | hypothetical protein |
| HMPREF0351_11099 | 16/16 | 1086772 | 1086930 | + | . | hypothetical protein |
| HMPREF0351_11161 | 11/16 | 1146938 | 1147132 | - | . | hypothetical protein |
| HMPREF0351_11172 | 15/16 | 1156956 | 1157198 | - | *gst* | glutathione S-transferase |
| HMPREF0351_11173 | 11/16 | 1157398 | 1157724 | + | . | conserved hypothetical protein |
| HMPREF0351_11175 | 13/16 | 1159116 | 1160114 | + | . | conserved hypothetical protein |
| HMPREF0351_11243 | 16/16 | 1232381 | 1232626 | + | . | esterase |
| HMPREF0351_11244 | 16/16 | 1232623 | 1232841 | + | . | lipase/acylhydrolase domain protein |
| HMPREF0351_11322 | 16/16 | 1306733 | 1307266 | - | . | conserved hypothetical protein |
| HMPREF0351_11362 | 16/16 | 1348924 | 1349055 | - | . | hypothetical protein |
| HMPREF0351_11378 | 13/16 | 1364133 | 1364306 | - | . | ComG operon protein 5 |
| HMPREF0351_11386 | 14/16 | 1372582 | 1372713 | + | . | hypothetical protein |
| HMPREF0351_11389 | 14/16 | 1374176 | 1374343 | + | . | hypothetical protein |
| HMPREF0351_11441 | 13/16 | 1429345 | 1429473 | - | . | hypothetical protein |
| HMPREF0351_11461 | 11/16 | 1450832 | 1450975 | - | . | hypothetical protein |
| HMPREF0351_11473 | 12/16 | 1461733 | 1461960 | - | . | hypothetical protein |
| HMPREF0351_11497 | 12/16 | 1476754 | 1477317 | - | . | prevent-host-death family antitoxin |
| HMPREF0351_11498 | 11/16 | 1477295 | 1477462 | + | . | hypothetical protein |
| HMPREF0351_11501 | 12/16 | 1478681 | 1478803 | - | . | hypothetical protein |
| HMPREF0351_11502 | 12/16 | 1478837 | 1479550 | - | . | HAD superfamily hydrolase |
| HMPREF0351_11503 | 10/16 | 1479543 | 1480661 | - | *uxuA2* | mannonate dehydratase |
| HMPREF0351_11504 | 10/16 | 1480645 | 1481088 | - | *.* | PTS family mannose/fructose/sorbose porter component IIA |
| HMPREF0351_11505 | 10/16 | 1481122 | 1481544 | - | *.* | conserved hypothetical protein |
| HMPREF0351_11506 | 10/16 | 1481587 | 1481988 | - | *.* | conserved hypothetical protein |
| HMPREF0351_11507 | 10/16 | 1482025 | 1482534 | - | *.* | PTS family mannose/fructose/sorbose porter component IIB |
| HMPREF0351_11508 | 10/16 | 1482556 | 1483413 | - | *manZ* | PTS family mannose/fructose/sorbose porter component IID |
| HMPREF0351_11509 | 10/16 | 1483431 | 1484264 | - | *manY* | PTS family mannose/fructose/sorbose porter component IIC |
| HMPREF0351_11510 | 10/16 | 1484278 | 1485075 | - | *.* | alcohol dehydrogenase |
| HMPREF0351_11511 | 10/16 | 1485108 | 1485392 | - | *.* | alcohol dehydrogenase |
| HMPREF0351_11512 | 11/16 | 1485389 | 1486399 | - | *serA2* | phosphoglycerate dehydrogenase |
| HMPREF0351_11513 | 12/16 | 1486392 | 1487459 | - | *gnd2* | phosphogluconate dehydrogenase (decarboxylating) |
| HMPREF0351_11514 | 12/16 | 1487458 | 1488306 | + | *gntR* | RpiR family transcriptional regulator |
| HMPREF0351_11520 | 8/16 | 1493579 | 1494736 | - | *.* | conserved hypothetical protein |
| HMPREF0351_11521 | 8/16 | 1494739 | 1495095 | - | *.* | conserved hypothetical protein |
| HMPREF0351_11524 | 10/16 | 1496563 | 1497402 | - | *abi* | abortive infection protein |
| HMPREF0351_11528 | 13/16 | 1500554 | 1501804 | + | *.* | IS116/IS110/IS902 family transposase |
| HMPREF0351_11539 | 3/16 | 1509469 | 1509615 | + | *.* | hypothetical protein |
| HMPREF0351_11547 | 11/16 | 1517612 | 1517737 | + | *.* | hypothetical protein |
| HMPREF0351_11601 | 13/16 | 1572603 | 1572791 | - | *.* | vitamin-B12 independent methionine synthase |
| HMPREF0351_11630 | 14/16 | 1608725 | 1609282 | - | *.* | conserved hypothetical protein |
| HMPREF0351_11631 | 12/16 | 1609461 | 1610072 | - | . | conserved hypothetical protein |
| HMPREF0351_11632 | 11/16 | 1610185 | 1610442 | - | . | conserved hypothetical protein |
| HMPREF0351_11633 | 12/16 | 1611039 | 1611317 | - | . | conserved hypothetical protein |
| HMPREF0351_11634 | 12/16 | 1611351 | 1611602 | - | *mviN* | integral membrane protein MviN |
| HMPREF0351_11636 | 11/16 | 1613125 | 1613727 | - | *.* | conserved hypothetical protein |
| HMPREF0351_11637 | 11/16 | 1613893 | 1614543 | - | *.* | conserved hypothetical protein |
| HMPREF0351_11638 | 15/16 | 1614534 | 1614734 | - | *.* | RNA 2-phosphotransferase |
| HMPREF0351_11670 | 16/16 | 1646972 | 1647118 | - | *.* | hypothetical protein |
| HMPREF0351_11671 | 16/16 | 1647099 | 1647212 | + | *.* | maltose ABC superfamily ATP binding cassette transporter, permease protein |
| HMPREF0351_11719 | 10/16 | 1696144 | 1697607 | - | *.* | transposase |
| HMPREF0351_11732 | 16/16 | 1708392 | 1708535 | - | *.* | hypothetical protein |
| HMPREF0351_11753 | 13/16 | 1729217 | 1729399 | + | *.* | hypothetical protein |
| HMPREF0351_11755 | 16/16 | 1731559 | 1731798 | + | *.* | hypothetical protein |
| HMPREF0351_11756 | 16/16 | 1731855 | 1731983 | + | *.* | hypothetical protein |
| HMPREF0351_11781 | 15/16 | 1751231 | 1751416 | + | *.* | hypothetical protein |
| HMPREF0351_11782 | 9/16 | 1751597 | 1752775 | - | *.* | bacteriophage integrase |
| HMPREF0351_11783 | 9/16 | 1752879 | 1753193 | - | *.* | conserved hypothetical protein |
| HMPREF0351_11784 | 9/16 | 1753300 | 1753515 | + | *.* | conserved hypothetical protein |
| HMPREF0351_11785 | 9/16 | 1753815 | 1754057 | - | *.* | conserved hypothetical protein |
| HMPREF0351_11786 | 9/16 | 1754044 | 1754502 | - | *rpoE* | DNA-directed RNA polymerase sigma subunit RpoE |
| HMPREF0351_11787 | 13/16 | 1754624 | 1756171 | - | . | transposase IS66 |
| HMPREF0351_11788 | 12/16 | 1756260 | 1756625 | - | . | transposon protein |
| HMPREF0351_11789 | 13/16 | 1756615 | 1756857 | - | . | conserved hypothetical protein |
| HMPREF0351_11790 | 9/16 | 1757298 | 1757672 | - | *gloA2* | lactoylglutathione lyase |
| HMPREF0351_11791 | 9/16 | 1757695 | 1759095 | - | *.* | beta-fructofuranosidase |
| HMPREF0351_11792 | 9/16 | 1759099 | 1759509 | - | *.* | PTS family mannose/fructose/sorbose porter component IIA |
| HMPREF0351_11793 | 9/16 | 1759527 | 1760027 | - | *manX2* | PTS family mannose/fructose/sorbose porter component IIB |
| HMPREF0351_11794 | 9/16 | 1760028 | 1760819 | - | *manZ2* | PTS family mannose/fructose/sorbose porter component IID |
| HMPREF0351_11795 | 9/16 | 1760812 | 1761585 | - | *manY2* | PTS family mannose/fructose/sorbose porter component IIC |
| HMPREF0351_11796 | 8/16 | 1761741 | 1764317 | - | *.* | PTS system transcriptional activator |
| HMPREF0351_11797 | 9/16 | 1764355 | 1765227 | - | *frk* | fructokinase |
| HMPREF0351_11798 | 9/16 | 1765263 | 1765730 | - | *crr* | PTS family glucose/glucoside (glc) porter component IIA |
| HMPREF0351_11799 | 9/16 | 1765740 | 1767209 | - | *scrA* | PTS family sucrose porter component IIBC |
| HMPREF0351_11800 | 9/16 | 1767280 | 1768698 | - | *.* | beta-fructofuranosidase |
| HMPREF0351_11801 | 9/16 | 1768820 | 1769800 | - | *scrR* | LacI family transcriptional regulator |
| HMPREF0351_11802 | 11/16 | 1770080 | 1770916 | - | . | integrase |
| HMPREF0351_11803 | 12/16 | 1770952 | 1771242 | - | . | IS3/IS911 family transposase |
| HMPREF0351_11804 | 8/16 | 1771335 | 1771844 | - | . | MarR family transcriptional regulator |
| HMPREF0351_11806 | 9/16 | 1773745 | 1774866 | - | . | transposase |
| HMPREF0351_11807 | 9/16 | 1775185 | 1776603 | + | . | transposase |
| HMPREF0351_11808 | 9/16 | 1776600 | 1777328 | + | . | AAA-superfamily ATP binding protein |
| HMPREF0351_11809 | 9/16 | 1777456 | 1778493 | - | . | conjugative transposon protein |
| HMPREF0351_11810 | 13/16 | 1778384 | 1779391 | - | . | lipoprotein |
| HMPREF0351_11811 | 13/16 | 1779388 | 1781565 | - | . | membrane protein |
| HMPREF0351_11812 | 13/16 | 1781818 | 1783005 | - | . | IS256 family transposase |
| HMPREF0351_11813 | 12/16 | 1783098 | 1785443 | - | . | ATPase |
| HMPREF0351_11814 | 13/16 | 1785430 | 1785819 | - | . | conjugative transposon protein |
| HMPREF0351_11815 | 9/16 | 1785869 | 1786765 | - | . | PHP domain protein |
| HMPREF0351_11816 | 8/16 | 1786768 | 1788615 | - | . | metal-dependent phosphoesterase |
| HMPREF0351_11817 | 13/16 | 1788712 | 1789212 | - | *ardA* | antirestriction protein ArdA |
| HMPREF0351_11818 | 14/16 | 1789225 | 1789377 | - | *.* | conserved hypothetical protein |
| HMPREF0351_11819 | 7/16 | 1789553 | 1791463 | - | *.* | RNA-directed DNA polymerase |
| HMPREF0351_11820 | 12/16 | 1792209 | 1792346 | - | *.* | conserved hypothetical protein |
| HMPREF0351_11821 | 13/16 | 1792343 | 1793527 | - | *.* | replication initiation family protein |
| HMPREF0351_11822 | 11/16 | 1793783 | 1794037 | - | *.* | conserved hypothetical protein |
| HMPREF0351_11823 | 12/16 | 1794062 | 1795204 | - | *.* | DNA (cytosine-5-)-methyltransferase |
| HMPREF0351_11824 | 13/16 | 1795264 | 1796616 | - | *.* | FtsK/SpoIIIE family cell division protein |
| HMPREF0351_11825 | 12/16 | 1796687 | 1797040 | - | *dinJ* | DNA-damage-inducible protein J |
| HMPREF0351_11826 | 13/16 | 1797041 | 1797415 | - | *.* | conjugative transposon protein |
| HMPREF0351_11827 | 13/16 | 1797428 | 1797742 | - | *.* | conjugative transposon protein |
| HMPREF0351_11828 | 13/16 | 1797856 | 1801083 | - | *fms18* | LPXTG family cell surface protein Fms18 |
| HMPREF0351_11829 | 9/16 | 1801162 | 1801824 | - | *.* | conserved hypothetical protein |
| HMPREF0351_11830 | 9/16 | 1802085 | 1802207 | + | *.* | hypothetical protein |
| HMPREF0351_11846 | 7/16 | 1815331 | 1816500 | + | *.* | transposase |
| HMPREF0351_11847 | 13/16 | 1816467 | 1816598 | + | *.* | ATP-dependent protease |
| HMPREF0351_11848 | 11/16 | 1816704 | 1817516 | + | *.* | transposase |
| HMPREF0351_11849 | 11/16 | 1817479 | 1818000 | + | *.* | conserved hypothetical protein |
| HMPREF0351_11850 | 4/16 | 1817989 | 1818147 | - | . | hypothetical protein |
| HMPREF0351_11854 | 15/16 | 1820636 | 1820842 | - | . | hypothetical protein |
| HMPREF0351_11855 | 13/16 | 1821214 | 1822401 | - | . | transposase |
| HMPREF0351_11858 | 13/16 | 1826059 | 1826295 | + | . | IS116/IS110/IS902 family transposase |
| HMPREF0351_11859 | 13/16 | 1826261 | 1826491 | + | . | IS116/IS110/IS902 family transposase |
| HMPREF0351_11860 | 13/16 | 1826473 | 1826772 | + | . | IS116/IS110/IS902 family transposase |
| HMPREF0351_11861 | 13/16 | 1826769 | 1827311 | + | . | IS116/IS110/IS902 family transposase |
| HMPREF0351_11864 | 12/16 | 1828779 | 1829039 | - | . | conserved hypothetical protein |
| HMPREF0351_11865 | 12/16 | 1829072 | 1829416 | - | . | DNA integrase |
| HMPREF0351_11866 | 13/16 | 1829580 | 1830116 | - | . | transposase IS3/IS911 |
| HMPREF0351_11868 | 13/16 | 1831575 | 1832111 | + | . | transposase IS3/IS911 |
| HMPREF0351_11869 | 12/16 | 1832108 | 1832620 | + | . | DNA integrase |
| HMPREF0351_11870 | 12/16 | 1832653 | 1832913 | + | . | conserved hypothetical protein |
| HMPREF0351_11914 | 5/16 | 1872987 | 1873493 | + | . | IS4 family transposase |
| HMPREF0351_11915 | 5/16 | 1873490 | 1874071 | + | . | IS4 family transposase |
| HMPREF0351_11916 | 2/16 | 1874185 | 1874346 | + | . | hypothetical protein |
| HMPREF0351_11920 | 5/16 | 1877094 | 1877315 | - | . | conserved hypothetical protein |
| HMPREF0351_11925 | 2/16 | 1880046 | 1880231 | + | . | conserved hypothetical protein |
| HMPREF0351_11929 | 2/16 | 1882737 | 1883003 | - | . | bacteriophage portal protein |
| HMPREF0351_11930 | 2/16 | 1883098 | 1883250 | - | . | hypothetical protein |
| HMPREF0351_11933 | 13/16 | 1885081 | 1886628 | - | . | transposase IS66 |
| HMPREF0351_11934 | 13/16 | 1886730 | 1887083 | - | . | transposase |
| HMPREF0351_11935 | 13/16 | 1887073 | 1887315 | - | . | conserved hypothetical protein |
| HMPREF0351_11937 | 2/16 | 1888133 | 1888300 | - | . | endo-1,4-beta-xylanase |
| HMPREF0351_11938 | 4/16 | 1888328 | 1889194 | - | . | AraC family response regulator |
| HMPREF0351_11939 | 6/16 | 1889217 | 1890869 | - | *pgm* | bifunctional phosphoglucomutase/phosphomannomutase |
| HMPREF0351_11941 | 4/16 | 1891388 | 1892086 | - | *pmi* | mannose-6-phosphate isomerase |
| HMPREF0351_11943 | 4/16 | 1892365 | 1893678 | - | *.* | mannose-1-phosphate guanylyltransferase (GDP) |
| HMPREF0351_11944 | 4/16 | 1893747 | 1894691 | - | *fcl* | GDP-L-fucose synthase |
| HMPREF0351_11945 | 4/16 | 1894713 | 1895768 | - | *gmd* | GDP-mannose 4,6-dehydratase |
| HMPREF0351_11951 | 4/16 | 1898021 | 1898602 | - | *rfbC2* | dTDP-4-dehydrorhamnose 3,5-epimerase |
| HMPREF0351_11962 | 3/16 | 1911689 | 1912105 | - | *cpsE* | glycosyl transferase |
| HMPREF0351_11963 | 6/16 | 1912664 | 1912882 | - | *ugd2* | UDP-glucose 6-dehydrogenase |
| HMPREF0351_11965 | 5/16 | 1914444 | 1914608 | - | *rgpAc* | alpha-D-GlcNAc alpha-1,2-L-rhamnosyltransferase |
| HMPREF0351_11966 | 9/16 | 1914961 | 1915650 | - | *rfbP2* | undecaprenyl-phosphate galactose phosphotransferase |
| HMPREF0351_11993 | 6/16 | 1942860 | 1944740 | - | *bglP2* | PTS family glucose/glucoside (glc) porter component IIABC |
| HMPREF0351_12004 | 13/16 | 1960073 | 1961620 | - | *.* | IS66 transposase |
| HMPREF0351_12005 | 13/16 | 1961722 | 1962075 | - | . | transposase |
| HMPREF0351_12006 | 13/16 | 1962065 | 1962307 | - | . | conserved hypothetical protein |
| HMPREF0351_12072 | 15/16 | 2019123 | 2019509 | - | . | polysaccharide deacetylase |
| HMPREF0351_12108 | 14/16 | 2053950 | 2054096 | - | . | hypothetical protein |
| HMPREF0351_12124 | 6/16 | 2072497 | 2073123 | - | . | conserved hypothetical protein |
| HMPREF0351_12125 | 7/16 | 2073418 | 2073753 | - | . | phage head-tail adaptor |
| HMPREF0351_12126 | 7/16 | 2073740 | 2074024 | - | . | bacteriophage DNA packaging protein |
| HMPREF0351_12127 | 7/16 | 2074080 | 2075603 | - | . | bifunctional family U35 bacteriophage prohead peptidase/major capsid protein |
| HMPREF0351_12128 | 7/16 | 2075596 | 2076771 | - | . | bacteriophage portal protein |
| HMPREF0351_12129 | 7/16 | 2076775 | 2076960 | - | . | hypothetical protein |
| HMPREF0351_12130 | 7/16 | 2076926 | 2078620 | - | . | bacteriophage terminase large subunit |
| HMPREF0351_12131 | 7/16 | 2078617 | 2079090 | - | . | bacteriophage terminase small subunit |
| HMPREF0351_12132 | 7/16 | 2079159 | 2079314 | - | . | hypothetical protein |
| HMPREF0351_12133 | 7/16 | 2079448 | 2079828 | - | . | bacteriophage endonuclease |
| HMPREF0351_12134 | 7/16 | 2079832 | 2080047 | - | . | group 1 glycosyl transferase |
| HMPREF0351_12135 | 7/16 | 2080050 | 2080457 | - | . | conserved hypothetical protein |
| HMPREF0351_12136 | 7/16 | 2080723 | 2082198 | - | *virE* | virulence-associated E family protein |
| HMPREF0351_12137 | 7/16 | 2082188 | 2083036 | - | . | bifunctional DNA primase/polymerase |
| HMPREF0351_12138 | 7/16 | 2083073 | 2083435 | - | . | conserved hypothetical protein |
| HMPREF0351_12139 | 6/16 | 2083436 | 2083594 | - | . | hypothetical protein |
| HMPREF0351_12140 | 6/16 | 2083735 | 2084139 | - | . | bacteriophage antirepressor protein |
| HMPREF0351_12141 | 6/16 | 2084120 | 2084359 | - | . | bacteriophage antirepressor protein |
| HMPREF0351_12142 | 7/16 | 2084356 | 2084667 | - | . | conserved hypothetical protein |
| HMPREF0351_12143 | 6/16 | 2084711 | 2085004 | - | . | conserved hypothetical protein |
| HMPREF0351_12144 | 6/16 | 2085198 | 2085845 | + | . | transcriptional regulator |
| HMPREF0351_12145 | 7/16 | 2085906 | 2087051 | + | . | bacteriophage integrase |
| HMPREF0351_12152 | 14/16 | 2091181 | 2092761 | - | . | ABC superfamily ATP binding cassette transporter, membrane protein |
| HMPREF0351_12153 | 13/16 | 2092754 | 2093497 | - | . | ABC superfamily ATP binding cassette transporter, ABC protein |
| HMPREF0351_12160 | 10/16 | 2099994 | 2101217 | - | . | family 88 glycosyl hydrolase |
| HMPREF0351_12161 | 10/16 | 2101211 | 2103109 | - | . | conserved hypothetical protein |
| HMPREF0351_12162 | 10/16 | 2103103 | 2104221 | - | . | family 88 glycosyl hydrolase |
| HMPREF0351_12163 | 10/16 | 2104304 | 2105137 | - | . | sugar ABC superfamily ATP binding cassette transporter, membrane protein |
| HMPREF0351_12164 | 10/16 | 2105130 | 2106047 | - | . | sugar ABC superfamily ATP binding cassette transporter, membrane protein |
| HMPREF0351_12165 | 10/16 | 2106070 | 2106249 | - | . | conserved hypothetical protein |
| HMPREF0351_12166 | 10/16 | 2106274 | 2107398 | - | . | sugar ABC superfamily ATP binding cassette transporter, binding protein |
| HMPREF0351_12167 | 10/16 | 2107581 | 2108489 | + | . | AraC family transcriptional regulator |
| HMPREF0351_12185 | 16/16 | 2126276 | 2126410 | - | . | hypothetical protein |
| HMPREF0351_12199 | 16/16 | 2143150 | 2143779 | + | . | acyl-CoA thioester hydrolase |
| HMPREF0351_12235 | 16/16 | 2181556 | 2181681 | - | . | hypothetical protein |
| HMPREF0351_12283 | 15/16 | 2231427 | 2232059 | + | . | conserved hypothetical protein |
| HMPREF0351_12284 | 15/16 | 2232182 | 2232820 | + | . | conserved hypothetical protein |
| HMPREF0351_12296 | 16/16 | 2240613 | 2240861 | - | . | GNAT family acetyltransferase |
| HMPREF0351_12297 | 16/16 | 2240852 | 2241139 | - | . | acetyltransferase |
| HMPREF0351_12347 | 10/16 | 2293649 | 2293912 | + | . | hypothetical protein |
| HMPREF0351_12352 | 13/16 | 2296940 | 2298127 | - | . | transposase |
| HMPREF0351_12366 | 12/16 | 2312434 | 2312580 | - | *entF* | enterocin induction factor |
| HMPREF0351_12367 | 12/16 | 2312684 | 2313022 | - | *entI* | enterocin A immunity protein |
| HMPREF0351_12368 | 12/16 | 2312997 | 2313194 | - | *entA* | enterocin A |
| HMPREF0351_12403 | 16/16 | 2343117 | 2344289 | - | . | ABC superfamily ATP binding cassette transporter, membrane protein |
| HMPREF0351_12404 | 16/16 | 2344276 | 2345421 | - | . | ABC superfamily ATP binding cassette transporter, membrane protein |
| HMPREF0351_12405 | 16/16 | 2345435 | 2346370 | - | . | ABC superfamily ATP binding cassette transporter, ABC protein |
| HMPREF0351_12406 | 16/16 | 2346469 | 2347563 | + | . | sensor histidine kinase |
| HMPREF0351_12407 | 16/16 | 2347560 | 2348189 | + | . | response regulator |
| HMPREF0351_12408 | 16/16 | 2348299 | 2348526 | - | . | conserved hypothetical protein |
| HMPREF0351_12420 | 10/16 | 2359695 | 2360990 | - | . | transposase |
| HMPREF0351_12447 | 16/16 | 2394545 | 2394688 | + | . | K+ uptake permease family protein |
| HMPREF0351_12448 | 16/16 | 2395033 | 2395161 | + | . | hypothetical protein |
| HMPREF0351_12449 | 16/16 | 2395266 | 2396849 | + | . | ABC superfamily ATP binding cassette transporter, ABC protein |
| HMPREF0351_12450 | 16/16 | 2397098 | 2397232 | - | . | hypothetical protein |
| HMPREF0351_12471 | 16/16 | 2416086 | 2416319 | + | . | hypothetical protein |
| HMPREF0351_12519 | 16/16 | 2480227 | 2480451 | + | . | conserved hypothetical protein |
| HMPREF0351_12521 | 16/16 | 2480634 | 2480846 | + | . | hypothetical protein |
| HMPREF0351_12522 | 12/16 | 2481428 | 2481988 | - | . | surface protein |
| HMPREF0351_12523 | 14/16 | 2482036 | 2485077 | - | . | Cna B-type |
| HMPREF0351_12524 | 15/16 | 2485779 | 2485928 | - | . | hypothetical protein |
| HMPREF0351_12525 | 15/16 | 2486185 | 2486304 | - | . | hypothetical protein |
| HMPREF0351_12529 | 15/16 | 2491776 | 2493329 | - | . | glycosyl hydrolase |
| HMPREF0351_12622 | 16/16 | 2601510 | 2602247 | - | . | short-chain dehydrogenase |
| HMPREF0351_12623 | 16/16 | 2602314 | 2602883 | - | . | acetyltransferase |
| HMPREF0351_12624 | 16/16 | 2603011 | 2603883 | + | . | LysR family transcriptional regulator |
| HMPREF0351_12641 | 10/16 | 2618665 | 2618808 | - | . | hypothetical protein |
| HMPREF0351_12647 | 16/16 | 2623886 | 2624170 | - | . | conserved hypothetical protein |
| HMPREF0351_12654 | 16/16 | 2629702 | 2630379 | - | *pgmB2* | beta-phosphoglucomutase |
| HMPREF0351_12655 | 15/16 | 2630376 | 2631506 | - | *glxK* | glycerate kinase |
| HMPREF0351_12656 | 16/16 | 2631506 | 2633689 | - | . | maltose/trehalose phosphorylase |
| HMPREF0351_12657 | 16/16 | 2633686 | 2634711 | - | . | dehydrogenase |
| HMPREF0351_12658 | 16/16 | 2634756 | 2635583 | - | . | sugar isomerase |
| HMPREF0351_12659 | 16/16 | 2635580 | 2636647 | - | . | alcohol dehydrogenase |
| HMPREF0351_12660 | 16/16 | 2636673 | 2637509 | - | . | sugar ABC superfamily ATP binding cassette transporter, membrane protein |
| HMPREF0351_12661 | 16/16 | 2637506 | 2638375 | - | . | sugar ABC superfamily ATP binding cassette transporter, membrane protein |
| HMPREF0351_12662 | 16/16 | 2638445 | 2639728 | - | . | sugar ABC superfamily ATP binding cassette transporter, binding protein |
| HMPREF0351_12663 | 16/16 | 2639742 | 2641427 | - | . | sucrose phosphorylase |
| HMPREF0351_12664 | 16/16 | 2641690 | 2642727 | + | . | LacI family transcriptional regulator |
