# Supplementary material for: Complete genome sequence of Enterococcus faecium strain TX16 and comparative genomic analysis of Enterococcus faecium genomes
Source: BMC Microbiol. 2012 Jul 7;12:135. doi: 10.1186/1471-2180-12-135 (PMC3433357; doi:10.1186/1471-2180-12-135)
Supplement: Additional file 4 — Table S2.Prophage loci and genes onE. faeciumTX16 genome. A table listing the two prophage loci, the predicted gene products within these two loci, and the corresponding ORFs in TX16. [file 1471-2180-12-135-S4.doc]

**Supplemental Table-Prophage loci and genes on *E. faecium* TX16 genome**

| **TX16 gene ID** | **Start** | **Stop** | **Strand** | **Gene Name** | **Gene Product Name** |
| --- | --- | --- | --- | --- | --- |
| Prophage 1 | 821259 | 857526 |  |  |  |
| HMPREF0351_10819 | 821259 | 822398 | - | *int* | bacteriophage integrase |
| HMPREF0351_10820 | 822515 | 823099 | - | - | prophage superinfection immunity protein |
| HMPREF0351_10821 | 823226 | 823666 | - | - | protein of hypothetical function DUF955 |
| HMPREF0351_10822 | 823675 | 823995 | - | - | bacteriophage transcriptional regulator |
| HMPREF0351_10823 | 824289 | 824429 | + | - | conserved hypothetical protein |
| HMPREF0351_10824 | 824560 | 824730 | + | - | hypothetical protein |
| HMPREF0351_10825 | 824727 | 824984 | + | - | phosphomannomutase/phosphoglucomutase-like protein |
| HMPREF0351_10826 | 824955 | 825263 | - | - | conserved hypothetical protein |
| HMPREF0351_10827 | 825341 | 826087 | + | - | BRO family transcriptional regulator |
| HMPREF0351_10828 | 826102 | 826305 | + | - | XRE family transcriptional regulator |
| HMPREF0351_10829 | 826321 | 826659 | + | - | protein of hypothetical function DUF771 |
| HMPREF0351_10830 | 826646 | 826825 | + | *sdhC* | succinate dehydrogenase cytochrome b558 subunit |
| HMPREF0351_10831 | 826868 | 827338 | - | *-* | conserved hypothetical protein |
| HMPREF0351_10832 | 827425 | 828123 | + | *rha* | bacteriophage regulatory protein Rha |
| HMPREF0351_10833 | 828116 | 828244 | + | - | hypothetical protein |
| HMPREF0351_10834 | 828301 | 828642 | + | - | conserved hypothetical protein |
| HMPREF0351_10835 | 828635 | 829306 | + | - | conserved hypothetical protein |
| HMPREF0351_10836 | 829312 | 829998 | + | - | conserved hypothetical protein |
| HMPREF0351_10837 | 830001 | 830750 | + | - | conserved hypothetical protein |
| HMPREF0351_10838 | 830762 | 831031 | + | - | conserved hypothetical protein |
| HMPREF0351_10839 | 831036 | 831200 | + | - | hypothetical protein |
| HMPREF0351_10840 | 831193 | 831495 | + | - | conserved hypothetical protein |
| HMPREF0351_10841 | 831492 | 831653 | + | - | staphylococcal accessory regulator R |
| HMPREF0351_10842 | 831650 | 831955 | + | - | conserved hypothetical protein |
| HMPREF0351_10843 | 831955 | 832311 | + | - | prophage pi3 protein 33 |
| HMPREF0351_10844 | 832298 | 832516 | + | - | cobalamin biosynthesis protein CobD |
| HMPREF0351_10845 | 832513 | 832932 | + | - | YopX protein |
| HMPREF0351_10846 | 832929 | 833486 | + | - | conserved hypothetical protein |
| HMPREF0351_10847 | 833483 | 833779 | + | - | conserved hypothetical protein |
| HMPREF0351_10848 | 833856 | 834269 | + | *arpU* | ArpU family phage transcriptional regulator |
| HMPREF0351_10849 | 834578 | 834730 | + | *-* | hypothetical protein |
| HMPREF0351_10850 | 834727 | 835002 | - | *-* | conserved hypothetical protein |
| HMPREF0351_10851 | 835456 | 835662 | + | *-* | hypothetical protein |
| HMPREF0351_10852 | 835858 | 836025 | + | *-* | thymidylate synthase |
| HMPREF0351_10853 | 836051 | 836395 | + | *mcrA* | HNH endonuclease |
| HMPREF0351_10854 | 836376 | 836681 | + | *-* | bacteriophage protein |
| HMPREF0351_10855 | 836784 | 837098 | + | *terS* | bacteriophage terminase small subunit |
| HMPREF0351_10856 | 837076 | 838770 | + | *terL* | bacreriophage terminase large subunit |
| HMPREF0351_10857 | 838772 | 839968 | + | *-* | bacteriophage portal protein |
| HMPREF0351_10858 | 839931 | 840617 | + | *clpP* | S14 family endopeptidase ClpP |
| HMPREF0351_10859 | 840617 | 841777 | + | *cps* | bacteriophage major capsid protein B |
| HMPREF0351_10860 | 841787 | 842662 | + | *-* | bacteriophage protein |
| HMPREF0351_10861 | 842659 | 842970 | + | - | bacteriophage protein |
| HMPREF0351_10862 | 842960 | 843313 | + | - | bacteriophage protein |
| HMPREF0351_10863 | 843303 | 843704 | + | - | bacteriophage protein |
| HMPREF0351_10864 | 843697 | 844101 | + | - | bacteriophage protein |
| HMPREF0351_10865 | 844113 | 844721 | + | *tsh* | bacteriophage major tail protein |
| HMPREF0351_10866 | 844740 | 845102 | + | *-* | bactriophage protein |
| HMPREF0351_10867 | 845147 | 845287 | + | *-* | hypothetical protein |
| HMPREF0351_10868 | 845304 | 848735 | + | *-* | conserved bacteriopahge tail protein |
| HMPREF0351_10869 | 848786 | 849523 | + | *-* | conserved bacteriophage protein |
| HMPREF0351_10870 | 849533 | 851824 | + | *-* | bactriophage minor structural protein |
| HMPREF0351_10871 | 851848 | 853974 | + | *-* | bactriophage minor structural protein |
| HMPREF0351_10872 | 853991 | 854140 | + | *-* | cytochrome c-type biogenesis protein CcmE |
| HMPREF0351_10873 | 854137 | 854583 | + | *-* | conserved hypothetical protein |
| HMPREF0351_10874 | 854585 | 854722 | + | *-* | conseved bactriophage protein |
| HMPREF0351_10875 | 854760 | 855053 | + | *-* | conserved bacteriophage protein |
| HMPREF0351_10876 | 855050 | 855274 | + | *hol* | bacteriophage holin |
| HMPREF0351_10877 | 855271 | 856296 | + | *amiD* | N-acetylmuramoyl-L-alanine amidase |
| HMPREF0351_t10003 | 856486 | 856559 | + | *-* | tRNA-Met |
| HMPREF0351_10878 | 857236 | 857526 | + | - | IS3/IS911 transposase |
|  |  |  |  |  |  |
| Prophage 2 | 2073418 | 2087051 |  |  |  |
| HMPREF0351_12125 | 2073418 | 2073753 | - | - | phage head-tail adaptor |
| HMPREF0351_12126 | 2073740 | 2074024 | - | - | bacteriophage DNA packaging protein |
| HMPREF0351_12127 | 2074080 | 2075603 | - | - | bifunctional family U35 bacteriophage prohead peptidase/major capsid protein |
| HMPREF0351_12128 | 2075596 | 2076771 | - | - | bacteriophage portal protein |
| HMPREF0351_12129 | 2076775 | 2076960 | - | - | hypothetical protein |
| HMPREF0351_12130 | 2076926 | 2078620 | - | - | bacteriophage terminase large subunit |
| HMPREF0351_12131 | 2078617 | 2079090 | - | - | bacteriophage terminase small subunit |
| HMPREF0351_12132 | 2079159 | 2079314 | - | - | hypothetical protein |
| HMPREF0351_12133 | 2079448 | 2079828 | - | - | bacteriophage endonuclease |
| HMPREF0351_12134 | 2079832 | 2080047 | - | - | group 1 glycosyl transferase |
| HMPREF0351_12135 | 2080050 | 2080457 | - | - | conserved hypothetical protein |
| HMPREF0351_12136 | 2080723 | 2082198 | - | *virE* | virulence-associated E family protein |
| HMPREF0351_12137 | 2082188 | 2083036 | - | - | bifunctional DNA primase/polymerase |
| HMPREF0351_12138 | 2083073 | 2083435 | - | - | conserved hypothetical protein |
| HMPREF0351_12139 | 2083436 | 2083594 | - | - | hypothetical protein |
| HMPREF0351_12140 | 2083735 | 2084139 | - | - | bacteriophage antirepressor protein |
| HMPREF0351_12141 | 2084120 | 2084359 | - | - | bacteriophage antirepressor protein |
| HMPREF0351_12142 | 2084356 | 2084667 | - | - | conserved hypothetical protein |
| HMPREF0351_12143 | 2084711 | 2085004 | - | - | conserved hypothetical protein |
| HMPREF0351_12144 | 2085198 | 2085845 | + | - | transcriptional regulator |
| HMPREF0351_12145 | 2085906 | 2087051 | + | - | bacteriophage integrase |
