# Supplementary material for: Complete genome sequence of Enterococcus faecium strain TX16 and comparative genomic analysis of Enterococcus faecium genomes
Source: BMC Microbiol. 2012 Jul 7;12:135. doi: 10.1186/1471-2180-12-135 (PMC3433357; doi:10.1186/1471-2180-12-135)
Supplement: Additional file 5 — Table S3.Mobile elements in theE. faeciumTX16 genome. A table listing all of the predicted mobile elements and their corresponding locus tags in TX16. [file 1471-2180-12-135-S5.doc]

| Locus Tag | Start | End | Predicted Gene/Family |
| --- | --- | --- | --- |
| **Chromosome** |  |  |  |
| HMPREF0351_10118 | 116500 | 117678 | ISEf1transposase |
| HMPREF0351_10120 | 119766 | 118813 | transposase |
| HMPREF0351_10147 | 143804 | 144982 | ISEf1 transposase |
| HMPREF0351_10172 | 169978 | 169442 | ISEnfa3 transposase |
| HMPREF0351_10253 | 246545 | 247714 | ISEf1 transposase |
| HMPREF0351_10256 | 249908 | 249699 | ISEf1 transposase |
| HMPREF0351_10259 | 251364 | 250807 | transposase |
| HMPREF0351_10264 | 257579 | 257316 | ISSag4 transposase |
| HMPREF0351_10265 | 257818 | 257672 | ISSdy1 transposase |
| HMPREF0351_10266 | 258152 | 257859 | transposase |
| HMPREF0351_10282 | 270298 | 271251 | transposase |
| HMPREF0351_10300 | 285708 | 286616 | ISEfm1 transposase |
| HMPREF0351_10312 | 292399 | 291446 | transposase |
| HMPREF0351_10314 | 293413 | 294708 | IS204/IS1001/IS1096/IS1165 family transposase |
| HMPREF0351_10322 | 303580 | 303275 | ISEf1 transposase |
| HMPREF0351_10323 | 304454 | 303726 | transposase |
| HMPREF0351_10364 | 347131 | 346595 | ISEnfa3 transposase |
| HMPREF0351_10524 | 517367 | 517480 | IS200 transposase |
| HMPREF0351_10606 | 606317 | 604854 | ISEfa7 transposase |
| HMPREF0351_10716 | 720130 | 719177 | transposase |
| HMPREF0351_10878 | 857236 | 857526 | IS3/IS911 transposase |
| HMPREF0351_10880 | 858977 | 858483 | transposase |
| HMPREF0351_10925 | 902753 | 902007 | transposase |
| HMPREF0351_10927 | 903083 | 903448 | transposon protein |
| HMPREF0351_10928 | 903537 | 905084 | transposase IS66 |
| HMPREF0351_10930 | 905697 | 906650 | transposase |
| HMPREF0351_10931 | 907300 | 906647 | IS204/IS1001/IS1096/IS1165 family transposase |
| HMPREF0351_10995 | 973254 | 971959 | transposase |
| HMPREF0351_11032 | 1016678 | 1017631 | transposase |
| HMPREF0351_11035 | 1021443 | 1020193 | IS116/IS110/IS902 family transposase |
| HMPREF0351_11079 | 1063888 | 1065183 | transposase |
| HMPREF0351_11174 | 1157808 | 1158986 | ISEf1 transposase |
| HMPREF0351_11364 | 1351019 | 1351972 | transposase |
| HMPREF0351_11457 | 1447110 | 1445932 | transposase |
| HMPREF0351_11460 | 1449882 | 1450835 | transposase |
| HMPREF0351_11468 | 1456563 | 1457522 | transposase |
| HMPREF0351_11528 | 1500554 | 1501804 | IS116/IS110/IS902 family transposase |
| HMPREF0351_11540 | 1509655 | 1510833 | transposase |
| HMPREF0351_11541 | 1511942 | 1510989 | transposase |
| HMPREF0351_11635 | 1612175 | 1613128 | transposase |
| HMPREF0351_11719 | 1697607 | 1696144 | transposase |
| HMPREF0351_11721 | 1699371 | 1698217 | ISEf1 transposase |
| HMPREF0351_11728 | 1704236 | 1705189 | transposase |
| HMPREF0351_11734 | 1710213 | 1709254 | IS30 family integrase |
| HMPREF0351_11735 | 1710426 | 1711130 | ISEfm1 transposase |
| HMPREF0351_11752 | 1728505 | 1727327 | ISEf1 transposase |
| HMPREF0351_11754 | 1730044 | 1731222 | ISEf1 transposase |
| HMPREF0351_11762 | 1737395 | 1736355 | ISEf1 transposase |
| HMPREF0351_11787 | 1756171 | 1754624 | transposase IS66 |
| HMPREF0351_11788 | 1756625 | 1756260 | transposon protein |
| HMPREF0351_11803 | 1771242 | 1770952 | IS3/IS911 family transposase |
| HMPREF0351_11805 | 1772183 | 1773136 | transposase |
| HMPREF0351_11806 | 1774866 | 1773745 | transposase |
| HMPREF0351_11807 | 1775185 | 1776603 | transposase |
| HMPREF0351_11809 | 1778493 | 1777456 | conjugative transposon protein |
| HMPREF0351_11812 | 1783005 | 1781818 | IS16 transposase |
| HMPREF0351_11814 | 1785819 | 1785430 | conjugative transposon protein |
| HMPREF0351_11826 | 1797415 | 1797041 | conjugative transposon protein |
| HMPREF0351_11827 | 1797742 | 1797428 | conjugative transposon protein |
| HMPREF0351_11846 | 1815331 | 1816500 | IS1476 transposase |
| HMPREF0351_11848 | 1816704 | 1817516 | transposase |
| HMPREF0351_11855 | 1822401 | 1821214 | IS16 transposase |
| HMPREF0351_11856 | 1823224 | 1824519 | ISEfa5 transposase |
| HMPREF0351_11858 | 1826059 | 1826295 | IS116/IS110/IS902 family transposase |
| HMPREF0351_11859 | 1826261 | 1826491 | IS116/IS110/IS902 family transposase |
| HMPREF0351_11860 | 1826473 | 1826772 | IS116/IS110/IS902 family transposase |
| HMPREF0351_11861 | 1826769 | 1827311 | IS116/IS110/IS902 family transposase |
| HMPREF0351_11866 | 1830116 | 1829580 | ISEnfa3 transposase |
| HMPREF0351_11867 | 1831405 | 1830230 | ISEf1 transposase |
| HMPREF0351_11868 | 1831575 | 1832111 | ISEnfa3 transposase |
| HMPREF0351_11885 | 1846475 | 1847770 | ISEfa5 transposase |
| HMPREF0351_11914 | 1872987 | 1873493 | IS4 transposase |
| HMPREF0351_11915 | 1873490 | 1874071 | IS4 transposase |
| HMPREF0351_11917 | 1874415 | 1875323 | ISEfm1 transposase |
| HMPREF0351_11921 | 1878180 | 1878509 | IS3/IS911 family transposase |
| HMPREF0351_11926 | 1881337 | 1880384 | transposase |
| HMPREF0351_11933 | 1886628 | 1885081 | transposase IS66 |
| HMPREF0351_11934 | 1887083 | 1886730 | transposase |
| HMPREF0351_11981 | 1931754 | 1933049 | ISEfa5 transposase |
| HMPREF0351_12004 | 1961620 | 1960073 | IS66 transposase |
| HMPREF0351_12005 | 1962075 | 1961722 | transposase |
| HMPREF0351_12169 | 2110894 | 2111847 | transposase |
| HMPREF0351_12201 | 2144437 | 2144766 | IS3/IS911 transposase |
| HMPREF0351_12203 | 2144993 | 2145637 | transposase |
| HMPREF0351_12261 | 2209945 | 2208935 | IS891/IS1136/IS1341 family transposase |
| HMPREF0351_12352 | 2298127 | 2296940 | IS16 transposase |
| HMPREF0351_12385 | 2330240 | 2329482 | ISEfm1 transposase |
| HMPREF0351_12420 | 2360990 | 2359695 | transposase |
| HMPREF0351_12520 | 2480461 | 2480640 | ISSmu2 transposase |
| HMPREF0351_10118 | 116500 | 117678 | transposase |
| **Plasmid 1** |  |  |  |
| HMPREF0351_12707 | 3750 | 4430 | IS1216 transposase |
| HMPREF0351_12712 | 7230 | 6967 | IS1485 transposase |
| HMPREF0351_12713 | 7610 | 7323 | IS1485 transposase |
| HMPREF0351_12715 | 8164 | 7835 | IS1485 transposase |
| HMPREF0351_12716 | 8480 | 8166 | transposase |
| HMPREF0351_12717 | 8785 | 8531 | IS3/IS911 family transposase |
| HMPREF0351_12722 | 13697 | 12738 | transposase |
| HMPREF0351_12726 | 15377 | 16075 | IS1216 transposase |
| HMPREF0351_12734 | 21718 | 22032 | conjugative transposon protein |
| HMPREF0351_12735 | 22048 | 22434 | conjugative transposon protein |
| HMPREF0351_12742 | 28842 | 29348 | conjugative transposon membrane protein |
| HMPREF0351_12743 | 29332 | 31779 | conjugative transposon protein |
| HMPREF0351_12744 | 31782 | 33959 | conjugative transposon membrane protein |
| HMPREF0351_12746 | 34954 | 35886 | conjugative transposon protein |
| **Plasmid 2** |  |  |  |
| HMPREF0351_12749 | 1201 | 515 | IS1216 transposase |
| HMPREF0351_12763 | 9679 | 10323 | IS1216 transposase |
| HMPREF0351_12764 | 10714 | 10385 | IS1485 transposase |
| HMPREF0351_12768 | 14177 | 15427 | IS116/IS110/IS902 family transposase |
| HMPREF0351_12770 | 17759 | 16581 | ISEf1 transposase |
| HMPREF0351_12775 | 22319 | 20892 | ISEfa12 transposase |
| HMPREF0351_12793 | 38753 | 38067 | IS256 transposase |
| HMPREF0351_12794 | 39537 | 38851 | IS1216 transposase |
| HMPREF0351_12803 | 45840 | 45166 | IS1182 transposase |
| HMPREF0351_12807 | 48065 | 48745 | IS1216 transposase |
| HMPREF0351_12809 | 49312 | 50499 | IS16 transposase |
| HMPREF0351_12813 | 52571 | 53251 | IS1216 transposase |
| **Plasmid 3** |  |  |  |
| HMPREF0351_12845 | 13936 | 13757 | transposase |
| HMPREF0351_12867 | 36428 | 35250 | ISEf1 transposase |
| HMPREF0351_12877 | 42335 | 41592 | ISPsy4 transposase |
| HMPREF0351_12878 | 43759 | 42335 | transposase |
| HMPREF0351_12887 | 54654 | 53107 | transposase IS66 |
| HMPREF0351_12888 | 55109 | 54756 | transposase |
| HMPREF0351_12891 | 57132 | 56443 | IS1297 transposase |
| HMPREF0351_12892 | 58385 | 57180 | ISEf1 transposase |
| HMPREF0351_12894 | 59354 | 58563 | IS1476 transposase |
| HMPREF0351_12900 | 65781 | 65200 | IS16 transposase |
| HMPREF0351_12901 | 66388 | 65753 | IS16 transposase |
| HMPREF0351_12904 | 68577 | 68732 | IS1297 transposase |
| HMPREF0351_12905 | 68799 | 69257 | IS1297 transposase |
| HMPREF0351_12910 | 72461 | 72003 | IS1297 transposase |
| HMPREF0351_12911 | 72683 | 72528 | transposase |
| HMPREF0351_12920 | 79785 | 79099 | IS1297 transposase |
| HMPREF0351_12924 | 85929 | 84631 | transposase |
| HMPREF0351_12931 | 91784 | 90948 | IS1485 transposase |
| HMPREF0351_12932 | 92149 | 91820 | IS1485 transposase |
| HMPREF0351_12934 | 92922 | 92674 | IS3 family transposon protein |
| HMPREF0351_12936 | 95380 | 94211 | IS256 transposase |
| HMPREF0351_12941 | 98787 | 99077 | IS3/IS911 family transposase |
| HMPREF0351_12942 | 99113 | 99724 | ISSdy1 transposon protein |
| HMPREF0351_12945 | 103782 | 103138 | transposase |
| HMPREF0351_12947 | 104078 | 104461 | transposase |
| HMPREF0351_12948 | 104533 | 106080 | IS66 transposase |
| HMPREF0351_12949 | 107503 | 106208 | ISEfa5 transposase |
| HMPREF0351_12950 | 107698 | 108990 | IS256 transposase |
| HMPREF0351_12956 | 114841 | 115794 | transposase |
| HMPREF0351_12957 | 117300 | 116005 | ISEfa5 transposase |
| HMPREF0351_12959 | 118758 | 117829 | transposase |
| HMPREF0351_12961 | 120690 | 121598 | ISEfm1 transposase |
| HMPREF0351_12974 | 133034 | 132690 | ISEfa5 transposase |
| HMPREF0351_12975 | 133357 | 134652 | ISEfa5 transposase |
| HMPREF0351_12976 | 135805 | 134780 | transposase IS66 |
| HMPREF0351_12979 | 136783 | 136430 | transposase |
| HMPREF0351_12982 | 137835 | 138830 | transposase |
| HMPREF0351_12985 | 141362 | 142636 | IS1476 transposase |
| HMPREF0351_12998 | 160481 | 159207 | IS1476 transposase |
| HMPREF0351_12999 | 160694 | 161896 | transposase |
| HMPREF0351_13000 | 162041 | 163336 | ISEfa5 transposase |
| HMPREF0351_13011 | 171846 | 171220 | ISEfa7 transposase |
| HMPREF0351_13012 | 172289 | 171885 | ISEfa7 transposase |
| HMPREF0351_13013 | 173460 | 172261 | mutator family transposase |
| HMPREF0351_13017 | 177159 | 177449 | ISEfa8 transposase |
| HMPREF0351_13021 | 179762 | 179893 | ISEfa8 transposase |
| HMPREF0351_13022 | 179894 | 180052 | ISEfa8 transposase |
| HMPREF0351_13023 | 180088 | 180924 | ISEfa8 transposase |
| HMPREF0351_13024 | 180925 | 181245 | IS256/ISEf1 family transposase |
| HMPREF0351_13026 | 181883 | 182836 | transposase |
| HMPREF0351_13028 | 183638 | 184546 | ISEfm1 transposase |
| HMPREF0351_13031 | 187276 | 185792 | ISEfa7 transposase |
| HMPREF0351_13033 | 188045 | 188374 | IS1485 transposase |
| HMPREF0351_13034 | 188410 | 189246 | IS1485 transposase |
| HMPREF0351_13035 | 191048 | 189753 | IS204/IS1001/IS1096/IS1165 family transposase |
| HMPREF0351_13041 | 195929 | 197224 | ISEfa5 transposase |
| HMPREF0351_13043 | 199463 | 198504 | transposase |
| HMPREF0351_13059 | 216847 | 217134 | transposase |
| HMPREF0351_13083 | 231885 | 231424 | IS1297 transposase |
| HMPREF0351_13084 | 232107 | 231952 | IS1297 transposase |
| HMPREF0351_13088 | 235431 | 234181 | IS116/IS110/IS902 family transposase |
| HMPREF0351_13090 | 237540 | 238730 | transposase |
| HMPREF0351_13110 | 248069 | 248473 | transposase |
| HMPREF0351_13111 | 248490 | 249638 | ISEfa4 transposase |
