# Supplementary material for: Complete genome sequence of Enterococcus faecium strain TX16 and comparative genomic analysis of Enterococcus faecium genomes
Source: BMC Microbiol. 2012 Jul 7;12:135. doi: 10.1186/1471-2180-12-135 (PMC3433357; doi:10.1186/1471-2180-12-135)
Supplement: Additional file 6 — Table S4.E. faeciumTX16 genomic islands and genes. A table listing the nine genomic islands, the genes and predicted products within those islands, and the corresponding ORFs and coordinates within TX16. [file 1471-2180-12-135-S6.doc]

**Supplemental Table-*E. faecium*** TX16 genomic islands and genes.

| **TX16 gene ID** | **Start** | **Stop** | **Strand** | **Gene Name** | **Gene Product Name** |
| --- | --- | --- | --- | --- | --- |
| GI1 | 245109 | 257859 |  |  |  |
| HMPREF0351_10251 | 245109 | 245720 | + | - | SPOUT methyltransferase superfamily protein |
| HMPREF0351_10252 | 245905 | 246474 | + | - | conserved hypothetical protein |
| HMPREF0351_10253 | 246545 | 247714 | + | - | ISEf1 transposase |
| HMPREF0351_10254 | 247996 | 248706 | + | - | cell wall protein |
| HMPREF0351_10255 | 249134 | 249526 | + | - | conserved hypothetical protein |
| HMPREF0351_10256 | 249699 | 249908 | - | - | ISEf1 transposase |
| HMPREF0351_10257 | 249868 | 250065 | + | - | hypothetical protein |
| HMPREF0351_10258 | 250108 | 250683 | *-* | *tdk* | thymidine kinase |
| HMPREF0351_10259 | 250807 | 251364 | *-* | *-* | transposase |
| HMPREF0351_10260 | 251821 | 253332 | *+* | *gadC* | APC family amino acid-polyamine-organocation transporter |
| HMPREF0351_10261 | 253345 | 254745 | *+* | *gadB* | glutamate decarboxylase |
| HMPREF0351_10262 | 254910 | 256373 | - | - | transcriptional regulator |
| HMPREF0351_10263 | 256739 | 257332 | + | - | ParB family nuclease |
| HMPREF0351_10264 | 257316 | 257579 | - | - | ISSag4 transposase |
| HMPREF0351_10265 | 257672 | 257818 | - | - | ISSdy1 transposase |
| GI2 | 513343 | 517480 |  |  |  |
| HMPREF0351_10516 | 513343 | 513690 | + | - | conserved hypothetical protein |
| HMPREF0351_10517 | 513666 | 513905 | + | - | conserved hypothetical protein |
| HMPREF0351_10518 | 514110 | 514448 | - | - | conserved hypothetical protein |
| HMPREF0351_10519 | 514729 | 514965 | + | - | AbrB family transcriptional regulator |
| HMPREF0351_10520 | 514965 | 515306 | + | *chpA* | ChpA/MazF transcriptional modulator |
| HMPREF0351_10521 | 516156 | 516341 | + | *-* | hypothetical protein |
| HMPREF0351_10522 | 516538 | 516705 | + | *-* | hypothetical protein |
| HMPREF0351_10523 | 516828 | 517040 | + | *-* | hypothetical protein |
| HMPREF0351_10524 | 517367 | 517480 | + | *-* | IS200 family transposase |
| GI3 | 1151876 | 1160671 |  |  |  |
| HMPREF0351_11165 | 1151181 | 1151924 | + | *-* | ABC superfamily ATP binding cassette transporter, membrane protein |
| HMPREF0351_11166 | 1151876 | 1152931 | + | *-* | ABC superfamily ATP binding cassette transporter, membrane protein |
| HMPREF0351_11167 | 1152943 | 1153680 | + | *-* | ABC superfamily ATP binding cassette transporter, ABC protein |
| HMPREF0351_11168 | 1153670 | 1154392 | + | *-* | TatD family magnesium (Mg2+)-dependent deoxyribonuclease |
| HMPREF0351_11169 | 1154476 | 1155099 | - | *-* | LysM family protein |
| HMPREF0351_11170 | 1155488 | 1156186 | + | *-* | cell wall protein |
| HMPREF0351_11171 | 1156394 | 1156684 | + | *-* | hypothetical protein |
| HMPREF0351_11172 | 1156956 | 1157198 | - | *gst* | glutathione S-transferase |
| HMPREF0351_11173 | 1157398 | 1157724 | + | *-* | conserved hypothetical protein |
| HMPREF0351_11174 | 1157808 | 1158986 | + | *-* | mutator family transposase |
| HMPREF0351_11175 | 1159116 | 1160114 | + | *-* | conserved hypothetical protein |
| HMPREF0351_t10020 | 1160351 | 1160437 | - | *-* | tRNA-Leu |
| HMPREF0351_11176 | 1160492 | 1160671 | + | *-* | hypothetical protein |
| GI4 | 1609282 | 1614534 |  |  |  |
| HMPREF0351_11631 | 1609461 | 1610072 | - | - | conserved hypothetical protein |
| HMPREF0351_11632 | 1610185 | 1610442 | - | - | conserved hypothetical protein |
| HMPREF0351_11633 | 1611039 | 1611317 | - | - | conserved hypothetical protein |
| HMPREF0351_11634 | 1611351 | 1611602 | - | *mviN* | integral membrane protein MviN |
| HMPREF0351_11635 | 1612175 | 1613128 | + | *-* | transposase |
| HMPREF0351_11636 | 1613125 | 1613727 | - | *-* | conserved hypothetical protein |
| HMPREF0351_11637 | 1613893 | 1614543 | - | *-* | conserved hypothetical protein |
| GI5 | 1827597 | 1832913 |  |  |  |
| HMPREF0351_11862 | 1827597 | 1827779 | + | *-* | conserved hypothetical protein |
| HMPREF0351_11863 | 1828087 | 1828518 | - | *-* | conserved hypothetical protein |
| HMPREF0351_11864 | 1828779 | 1829039 | - | *-* | conserved hypothetical protein |
| HMPREF0351_11865 | 1829072 | 1829416 | - | *-* | DNA integrase |
| HMPREF0351_11866 | 1829580 | 1830116 | - | *-* | transposase IS3/IS911 |
| HMPREF0351_11867 | 1830230 | 1831405 | - | *-* | transposase |
| HMPREF0351_11868 | 1831575 | 1832111 | + | *-* | transposase IS3/IS911 |
| HMPREF0351_11869 | 1832108 | 1832620 | + | *-* | DNA integrase |
| HMPREF0351_11870 | 1832653 | 1832913 | + | *-* | conserved hypothetical protein |
| GI6 | 1878180 | 1884283 |  |  |  |
| HMPREF0351_11921 | 1878180 | 1878509 | + | *-* | IS3/IS911 family transposase |
| HMPREF0351_11922 | 1878545 | 1879381 | + | *-* | integrase |
| HMPREF0351_11923 | 1879570 | 1879692 | - | *-* | hypothetical protein |
| HMPREF0351_11924 | 1879787 | 1880047 | - | *-* | conserved hypothetical protein |
| HMPREF0351_11925 | 1880046 | 1880231 | + | *-* | conserved hypothetical protein |
| HMPREF0351_11926 | 1880384 | 1881337 | - | *-* | transposase |
| HMPREF0351_11927 | 1881420 | 1882094 | - | *wzx* | MOP superfamily multidrug/oligosaccharidyl-lipid/polysaccharide flippase transporter |
| HMPREF0351_11928 | 1882309 | 1882476 | - | - | hypothetical protein |
| HMPREF0351_11929 | 1882737 | 1883003 | - | - | bacteriophage portal protein |
| HMPREF0351_11930 | 1883098 | 1883250 | - | - | hypothetical protein |
| HMPREF0351_11931 | 1883900 | 1884316 | - | - | hypothetical protein |
| GI7 | 1887073 | 1898602 |  |  |  |
| HMPREF0351_11934 | 1886730 | 1887083 | - | - | transposase |
| HMPREF0351_11935 | 1887073 | 1887315 | - | - | conserved hypothetical protein |
| HMPREF0351_11936 | 1887590 | 1888039 | - | - | DNA binding protein |
| HMPREF0351_11937 | 1888133 | 1888300 | - | - | endo-1,4-beta-xylanase |
| HMPREF0351_11938 | 1888328 | 1889194 | - | - | AraC family response regulator |
| HMPREF0351_11939 | 1889217 | 1890869 | - | *pgm* | bifunctional phosphoglucomutase/phosphomannomutase |
| HMPREF0351_11940 | 1890871 | 1891386 | - | *nudF2* | ADP-ribose diphosphatase |
| HMPREF0351_11941 | 1891388 | 1892086 | - | *pmi* | mannose-6-phosphate isomerase |
| HMPREF0351_11942 | 1892180 | 1892344 | - | *-* | conserved hypothetical protein |
| HMPREF0351_11943 | 1892365 | 1893678 | - | *-* | mannose-1-phosphate guanylyltransferase (GDP) |
| HMPREF0351_11944 | 1893747 | 1894691 | - | *fcl* | GDP-L-fucose synthase |
| HMPREF0351_11945 | 1894713 | 1895768 | - | *gmd* | GDP-mannose 4,6-dehydratase |
| HMPREF0351_11946 | 1896084 | 1896251 | - | *-* | WecB/TagA/CpsF family glycosyl transferase |
| HMPREF0351_11947 | 1896269 | 1896400 | - | *-* | hypothetical protein |
| HMPREF0351_11948 | 1896393 | 1897184 | - | *-* | nucleoside-diphosphate sugar epimerase |
| HMPREF0351_11949 | 1897618 | 1897797 | - | *smtA* | metallothionein SmtA |
| HMPREF0351_11950 | 1897824 | 1897961 | - | *-* | hypothetical protein |
| HMPREF0351_11951 | 1898021 | 1898602 | - | *rfbC2* | dTDP-4-dehydrorhamnose 3,5-epimerase |
| GI8 | 1908202 | 1912882 |  |  |  |
| HMPREF0351_11959 | 1908202 | 1909452 | - | *ugd* | UDP-glucose/GDP-mannose dehydrogenase family protein |
| HMPREF0351_11960 | 1909472 | 1910530 | - | *-* | UDP-glucuronate 5'-epimerase |
| HMPREF0351_11961 | 1910564 | 1911358 | - | *wecB* | glycosyltransferase |
| HMPREF0351_11962 | 1911689 | 1912105 | - | *cpsE* | glycosyl transferase |
| HMPREF0351_11963 | 1912664 | 1912882 | - | *ugd2* | UDP-glucose 6-dehydrogenase |
| GI9 | 2309278 | 2312997 |  |  |  |
| HMPREF0351_12363 | 2309438 | 2310298 | - | *-* | DegV family protein |
| HMPREF0351_12364 | 2310285 | 2310875 | - | *-* | conserved hypothetical protein |
| HMPREF0351_t10034 | 2311235 | 2311307 | + | *-* | tRNA-Lys |
| HMPREF0351_12365 | 2311484 | 2312437 | + | *-* | integrase |
| HMPREF0351_12366 | 2312434 | 2312580 | - | *entF* | enterocin induction factor |
| HMPREF0351_12367 | 2312684 | 2313022 | - | *entI* | enterocin A immunity protein |
