# Supplementary material for: Complete genome sequence of Enterococcus faecium strain TX16 and comparative genomic analysis of Enterococcus faecium genomes
Source: BMC Microbiol. 2012 Jul 7;12:135. doi: 10.1186/1471-2180-12-135 (PMC3433357; doi:10.1186/1471-2180-12-135)
Supplement: Additional file 7 — Figure S3.ORF composition of the downstream extension of theepagene cluster in the 22E. faeciumgenomes (HMPREF0351_10908 - HMPREF0351_10923 in TX16). A figure depicting the predicted polysaccharide-encoding gene clusters found in the E. faecium genomes. [file 1471-2180-12-135-S7.ppt]

## Slide 1
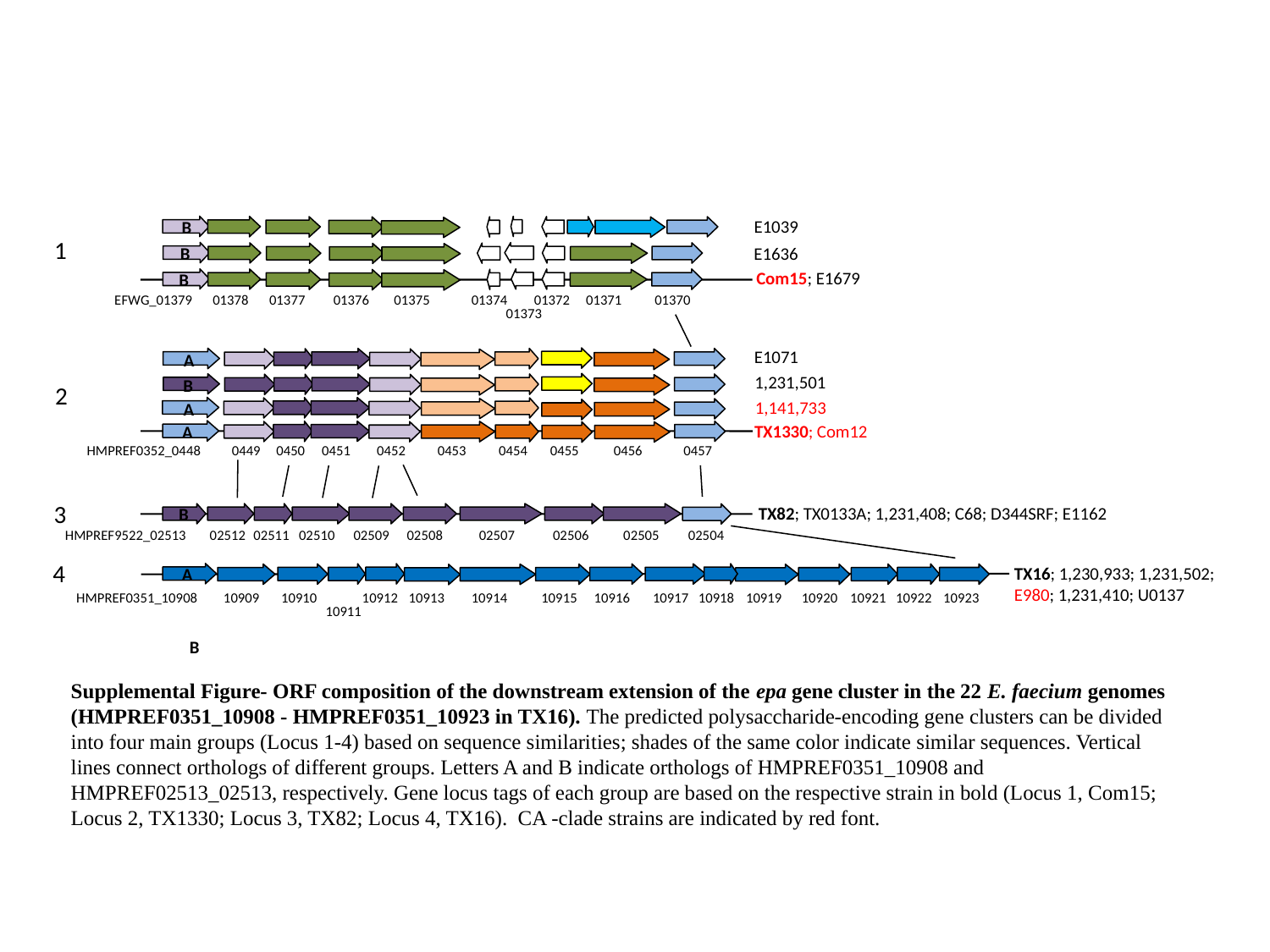

E1039
E1636
Com15; E1679
EFWG_01379
01378
01377
01376
01375
01374
01372
01371
01370
01373
E1071
1,231,501
1,141,733
TX1330; Com12
HMPREF0352_0448
0449
0450
0451
0452
0453
0454
0455
0456
0457
TX82; TX0133A; 1,231,408; C68; D344SRF; E1162
HMPREF9522_02513
02512
02511
02510
02509
02508
02507
02506
02505
02504
TX16; 1,230,933; 1,231,502;
E980; 1,231,410; U0137
HMPREF0351_10908
10909
10910
10912
10913
10914
10915
10916
10917
10918
10919
10920
10921
10922
10923
10911
B
1
B
B
A
B
A
A
2
3
B
4
A
B
Supplemental Figure- ORF composition of the downstream extension of the epa gene cluster in the 22 E. faecium genomes (HMPREF0351_10908 - HMPREF0351_10923 in TX16). The predicted polysaccharide-encoding gene clusters can be divided into four main groups (Locus 1-4) based on sequence similarities; shades of the same color indicate similar sequences. Vertical lines connect orthologs of different groups. Letters A and B indicate orthologs of HMPREF0351_10908 and HMPREF02513_02513, respectively. Gene locus tags of each group are based on the respective strain in bold (Locus 1, Com15; Locus 2, TX1330; Locus 3, TX82; Locus 4, TX16). CA -clade strains are indicated by red font.
