# Supplementary material for: Complete genome sequence of Enterococcus faecium strain TX16 and comparative genomic analysis of Enterococcus faecium genomes
Source: BMC Microbiol. 2012 Jul 7;12:135. doi: 10.1186/1471-2180-12-135 (PMC3433357; doi:10.1186/1471-2180-12-135)
Supplement: Additional file 8 — Table S5.Presence of genes encoding MSCRAMMs and pilins among 21E. faeciumgenomes. A table listing the different MSCRAMM and pilin variants present in each of the 22 genomes. [file 1471-2180-12-135-S8.doc]

Supplemental Table-Presence of genes encoding MSCRAMMs and pilins among 21 *E. faecium* genomes.

|  | ***ebp* operon** | | | ***fms11-fms19-fms16* cluster** | | | ***fms21-fms20* cluster** | |
| --- | --- | --- | --- | --- | --- | --- | --- | --- |
| **Strain** | ***ebpA*** | ***ebpB*** | ***ebpC*** | ***fms11*** | ***fms19*** | ***fms16*** | ***fms21*** | ***fms20*** |
| **HA Clade** |  |  |  |  |  |  |  |  |
| **TX16** | *ebp*A var. 1*^a^* | *ebpB* var. 1 | *ebpC* var. 1 | *fms11* var. 1 | *fms19*  var. 1(p)*^b^* | *fms16* var. 1(p) | *fms21* | *fms20* var. 1 |
| **1,231,502** | *ebp*A var. 1 | *ebpB* var. 1 | *ebpC* var. 1 | *fms11* var. 1 | *fms19*  var. 1(p) | *fms16* var. 1(p) | *fms21* | *fms20* var. 1 |
| **1,231,410** | *ebpA* var. 1 (p) | *ebpB* var. 1 | *ebpC* var. 1 | *fms11* var. 1 | *fms19*  var. 1(p) | *fms16* var. 1(p) | *fms21* (p) | "*fms20'* var. 2*^c^* |
| **1,231,501** | *ebp*A var. 1 | *ebpB* var. 1 | *ebpC* var. 1 | *fms11* var. 1 | *fms19*  var. 1 | *fms16* var. 2*^d^* | *fms21* | *fms20* var. 1 |
| **1,230,933** | *ebp*A var. 1 | *ebpB* var. 1 | *ebpC* var. 1 | *fms11* var. 1 | *fms19*  var. 1(p) | *fms16* var. 1(p) | *fms21* (p) | *fms20* var. 1 |
| **C68** | *ebpA* var. 1 (p) | *ebpB* var. 1 | *ebpC* var. 1 | *fms11* var. 1 | *fms19*  var. 1(p) | *fms16* var. 1(p) | *fms21* | *fms20* var. 1 |
| **D344SRF** | - | - | - | *fms11* var. 1 | *fms19*  var. 1(p) | *fms16* var. 1(p) | *fms21* (p) *fms21* (p) | *fms20* var. 1 "*fms20”* var. 2 |
| **U0137** | *ebp*A var. 1 | *ebpB* var. 1 | *ebpC* var. 1 | *fms11* var. 1(p) | *fms19*  var. 1 | *fms16* var. 1(p) | *fms21* | *fms20* var. 1 |
| **E1162** | *ebp*A var. 1 | *ebpB* var. 1 | *ebpC* var. 1 | *fms11* var. 1 | *fms19*  var. 1(p) | *fms16* var. 1(p) | *fms21* | *"fms20"* var. 2 |
| **E1636** | - | - | - | - | - | - | *fms21* (p) *fms21* (p) | *fms20* var. 1 (p) |
| **E1679** | - | - | - | *fms11* var. 1 | *fms19*  var. 1(p) | *fms16* var. 1(p) | - | - |
| **TX82** | *ebp*A var. 1 | *ebpB* var. 1 | *ebpC* var. 1 | *fms11* var. 1 | *fms19*  var. 1(p) | *fms16* var. 1(p) | - | - |
| **TX0133A** | *ebp*A var. 1 | *ebpB* var. 1 (p) | *ebpC* var. 1 | *fms11* var. 1 | *fms19*  var. 1(p) | *fms16* var. 1(p) | *fms21* | "*fms20"* var. 2 |
| **E1071** | *ebp*A var. 1 (p)*^e^* | - | *ebpC* var. 1 (p)*^e^* | - | - | - | *fms21* | *fms20* var. 1 |
| **E1039** | - | - | - | *fms11* var. 1(p) | - | - | *fms21* | *fms20* var. 1 |
| **1231408*^f^*** | *ebp*A var. 1 | *ebpB* var. 1 | *ebpC* var. 1 | *fms11* var. 1 | *fms19*  var. 1(p) | *fms16* var. 1(p) | *fms21* | *fms20* var. 1 |
| **CA**  **Clade** |  |  |  |  |  |  |  |  |
| **1,141,733** | *ebp*A var. 2*^g^* | *ebpB* var. 2*^h^* | *ebpC* var. 2*^i^* | *fms11* var. 2*^j^* | *fms19*  var. 2*^k^* | *fms16* var. 2 | *fms21* | *fms20* var. 1 |
| **TX1330** | *ebpA* var. 1 (p) *ebpA* var. 2 (p) | *ebpB* var. 1 *ebpB* var. 2 (p) | *ebpC* var. 1 *ebpC* var. 2 | - | - | - | *fms21* | *fms20* var. 1 |
| **Com12** | *ebpA* var. 1 (p) *ebpA* var. 2 (p) | *ebpB* var. 1 *ebpB* var. 2 (p) | *ebpC* var. 1 *ebpC* var. 2 | - | - | - | *fms21* | *fms20* var. 1 |
| **Com15** | *ebpA* var. 1 (p) *ebpA* var. 2 (p) | *ebpB* var. 1 *ebpB* var. 2 | *ebpC* var. 1 (p) *ebpC* var. 2 (p) | *fms11* var. 2 (p) | *fms19*  var. 2(p) | *fms16* var. 2(p) | *fms21* | *fms20* var. 1 |
| **E980** | *ebp*A var. 2 (p) | *ebpB* var. 2 | *ebpC* var. 2 (p) | *fms11* var. 2 (p) | *fms19*  var. 2(p) | *fms16* var. 2 | *fms21* | *fms20* var. 1 |

| |  | |  |  | |  | |  | |  | | | | | --- | --- | --- | --- | --- | --- | --- | --- | --- | --- | --- | --- | --- | |  | ***fms14-fms17*-*fms13* cluster** | | | | | | |  | |  |  |  | | **Strain** | ***fms14*** | | | ***fms17*** | | ***fms13*** | | ***scm*** | | ***fms18*** | ***fms15*** | ***acm*** | | **HA**  **Clade** |  | | |  | |  | |  | |  |  |  | | **TX16** | *fms14* var. 1 | | | *fms17* var. 1 | | *fms13* var. 1 | | *scm* var. 1 | | *fms18* var. 1 | *fms15* (p) | *acm* | | **1,231,502** | *fms14* var. 1 | | | *fms17* var. 1 | | *fms13* var. 1 | | *scm* var. 1 (p) | | *fms18* var. 1 *fms18* var. 2*^l^* | *fms15* (p) | *acm* | | **1,231,410** | *fms14* var. 1 | | | *fms17* var. 1 | | *fms13* var. 1 | | *scm* var. 1 (p) | | *fms18* var. 1 *fms18* var. 2 | *fms15* (p) | *acm* | | **1,231,501** | *fms14* var. 1 | | | *fms17* var. 1 | | *fms13* var. 1 | | *scm* var. 1 | | - | *fms15* (p) | *acm* | | **1,230,933** | *fms14* var. 1 | | | *fms17* var. 1 | | *fms13* var. 1 | | *scm* var. 1 (p) | | *fms18* var. 2 | *fms15* (p) | *acm* | | **C68** | *fms14* var. 1 | | | *fms17* var. 1 | | *fms13* var. 1 | | *scm* var. 1 (p) | | *fms18* var. 1 *fms18* var. 2 | *fms15* (p) | *acm* | | **D344SRF** | *fms14* var. 1 (p) | | | *fms17* var. 1 (p) | | *fms13* var. 1 (p) | | *scm* var. 1 (p) | | *fms18* var. 1 | *fms15* | *acm* (p) | | **U0137** | *fms14* var. 1 | | | *fms17* var. 1 | | *fms13* var. 1 | | *scm* var. 1 (p) | | *fms18* var. 2 | *fms15* (p) | *acm* | | **E1162** | *fms14* var. 1 | | | *fms17* var. 1 | | *fms13* var. 1 | | *scm* var. 1 (p) | | *fms18* var. 1 *fms18* var. 2 | *fms15* | *acm* | | **E1636** | *fms14* var. 1 | | | *fms17* var. 1 (p) | | *fms13* var. 1 (p) | | *scm* var. 1 (p) | | *fms18* var. 2 | *fms15* | *acm* (p) | | **E1679** | *fms14* var. 1 | | | *fms17* var. 1 (p) | | *fms13* var. 1 (p) | | *scm* var. 1 | | *fms18* var. 1 *fms18* var. 2 | *fms15* (p) | *acm* (p) | | **TX82** | *fms14* var. 1 | | | *fms17* var. 1 | | *fms13* var. 1 | | *scm* var. 1 (p) | | - | *fms15* (p) | *acm* | | **TX0133A** | *fms14* var. 1 | | | *fms17* var. 1 (p) | | *fms13* var. 1 (p) | | *scm* var. 1 (p) | | *fms18* var. 1 *fms18* var. 2 | *fms15* (p) | *acm* | | **E1071** | *fms14* var. 1 | | | *fms17* var. 1 | | *fms13* var. 1 | | *scm* var. 1 (p) | | *fms18* var. 2 | *fms15* (p) | *acm* (p) | | **E1039** | *fms14* var. 1 (p) | | | *fms17* var. 1 | | *fms13* var. 1 | | *scm* var. 1 (p) | | - | *fms15* (p) | *acm* (p) | | **1231408*^f^*** | *fms14* var. 2*^m^* | | | *fms17* var. 2*^n^* | | *fms13* var. 2*^o^* | | *scm* var. 1 (p) | | - | *fms15* (p) | *acm* | | **CA**  **Clade** |  | | |  | |  | |  | |  |  |  | | **1,141,733** | *fms14* var. 2 | | | *fms17* var. 2 | | *fms13* var. 2 | | *scm* var. 2 (p)*^p^* | | - | - | *acm* | | **TX1330** | *fms14* var. 2 | | | *fms17* var. 2 | | *fms13* var. 2 | | *scm* var. 2 | | - | - | *acm* (p) | | **Com12** | *fms14* var. 2 | | | *fms17* var. 2 | | *fms13* var. 2 | | *scm* var. 2 (p) | | - | - | *acm* (p) | | **Com15** | *fms14* var. 2 (p) | | | *fms17* var. 2 | | *fms13* var. 2 | | *scm* var. 2 (p) | | - | - | *acm* (p) | | **E980** | *fms14* var. 2 | | | *fms17* var. 2 | | *fms13* var. 2 | | - | | - | - | *acm* (p) |  | *^a^* var. 1, variant 1; var. 2; variant 2.  Cut-off for var.2 was set at <95% aa identity versus TX16 using PBLAST. | | |  | | |  | | | |  | | | |  | | | |  | | | | |  | | | | --- | --- | --- | --- | --- | --- | --- | --- | --- | --- | --- | --- | --- | --- | --- | --- | --- | --- | --- | --- | --- | --- | --- | --- | --- | --- | | *^b^*(p), partial, incomplete gene sequence or pseudogene | | | | |  | | | |  | | | |  | | | |  | | | | |  | | | | | *^c^* aa identities/similarities of proteins encoded by "*fms20*" var. 2 genes range between 39-43%/55-57% versus that of *fms20* var. 1 of TX16 | | | | | | | | | | | | | | | | | | | | | | | | |  | | *^d^* aa identities/similarities of proteins encoded by *fms16* var. 2 genes range between 93-94%/96-98% versus that of fms16 var. 1 of TX16  *^e^ebpA* and *ebpC* are fused into a single ORF in E1071 (5' region of *ebpA* (nt 1-2757) joined to 3' region of *ebpC* (nt 1083-1875)) | | | | | | | | | | | | | | | | | | | | | | | | |  | | *^f^* Strain 1,231,408 is a hybrid of HA and CA clades; see text for details | | | | | | | |  | | | |  | | | |  | | | | |  | | | |  | | *^g^* aa identities/similarities of proteins encoded by *ebpA* var. 2 genes range between 85-90%/80-82% versus that of *ebpA* var. 1 of TX16 |  |  | |  | | |  | | | |  | | | |  | | | |  | | | | |  | | | *^h^* aa identities/similarities of proteins encoded by *ebpB* var. 2 genes range between 87-92%/92-95% versus that of *ebpB* var. 1 of TX16 | | | | | | | | | | | | | | | | | | | | | | | | |  | | *^i^* aa identities/similarities of proteins encoded by *ebpC* var. 2 genes range between 90-94%/97-98% versus that of *ebpC* var. 1 of TX16 | | | | | | | | | | | | | | | | | | | | | | | | |  | | *^j^* aa identities/similarities of proteins encoded by *fms11* var. 2 genes range between 79-92%/85-95% versus that of *fms11* var. 1 of TX16  *^k^* aa identities/similarities of proteins encoded by *fms19* var. 2 genes range between 89-90%/94-95% versus that of *fms19* var. 1 of TX16 | | | | | | | | | | | | | | | | | | | | | | | | |  | | *^l^* aa identities/similarities of proteins encoded by *fms18* var. 2 genes range between 90-93%/94-96% versus that of *fms18* var. 1 of TX16 | | | | | | | | | | | | | | | | | | | | | | | | |  | | *^m^* aa identities/similarities of proteins encoded by *fms14* var. 2 genes range between 85-86%/92-94% versus that of *fms14* var. 1 of TX16 | | | | | | | | | | | | | | | | | | | | | | | | |  | | *^n^* aa identities/similarities of proteins encoded by *fms17* var. 2 genes are 91%/96% versus that of *fms17* var. 1 of TX16 | | | | | | | | | | | | | | | | | | | |  | | | | |  | | ^o^ aa identities/similarities of proteins encoded by *fms13* var. 2 genes range between 66-68%/80-82% versus that of *fms13* var. 1 of TX16 | | | | | | | | | | | | | | | | | | | | | | | | |  | | *^p^* aa identities/similarities of proteins encoded by *scm* var. 2 genes range between 94-95%/96-97% versus that of *scm* var. 1 of TX16  *^q^* TC6 was left out of this analysis as it is a transconjugant, and therefore not a unique genome | | | | | | | | | | | | | | | | | | | | | | | | |  | |  |  |  |  |  |  |  |  |  |
| --- | --- | --- | --- | --- | --- | --- | --- | --- | --- | --- | --- | --- | --- | --- | --- | --- | --- | --- | --- | --- | --- | --- | --- | --- | --- | --- | --- | --- | --- | --- | --- | --- | --- | --- | --- | --- | --- | --- | --- | --- | --- | --- | --- | --- | --- | --- | --- | --- | --- | --- | --- | --- | --- | --- | --- | --- | --- | --- | --- | --- | --- | --- | --- | --- | --- | --- | --- | --- | --- | --- | --- | --- | --- | --- | --- | --- | --- | --- | --- | --- | --- | --- | --- | --- | --- | --- | --- | --- | --- | --- | --- | --- | --- | --- | --- | --- | --- | --- | --- | --- | --- | --- | --- | --- | --- | --- | --- | --- | --- | --- | --- | --- | --- | --- | --- | --- | --- | --- | --- | --- | --- | --- | --- | --- | --- | --- | --- | --- | --- | --- | --- | --- | --- | --- | --- | --- | --- | --- | --- | --- | --- | --- | --- | --- | --- | --- | --- | --- | --- | --- | --- | --- | --- | --- | --- | --- | --- | --- | --- | --- | --- | --- | --- | --- | --- | --- | --- | --- | --- | --- | --- | --- | --- | --- | --- | --- | --- | --- | --- | --- | --- | --- | --- | --- | --- | --- | --- | --- | --- | --- | --- | --- | --- | --- | --- | --- | --- | --- | --- | --- | --- | --- | --- | --- | --- | --- | --- | --- | --- | --- | --- | --- | --- | --- | --- | --- | --- | --- | --- | --- | --- | --- | --- | --- | --- | --- | --- | --- | --- | --- | --- | --- | --- | --- | --- | --- | --- | --- | --- | --- | --- | --- | --- | --- | --- | --- | --- | --- | --- | --- | --- | --- | --- | --- | --- | --- | --- | --- | --- | --- | --- | --- | --- | --- | --- | --- | --- | --- | --- | --- | --- | --- | --- | --- | --- | --- | --- | --- | --- | --- | --- | --- | --- | --- | --- | --- | --- | --- | --- | --- | --- | --- | --- | --- | --- | --- | --- | --- | --- | --- | --- | --- | --- | --- | --- | --- | --- | --- | --- | --- | --- | --- | --- | --- | --- | --- | --- | --- | --- | --- | --- | --- | --- | --- | --- | --- | --- | --- | --- | --- | --- | --- | --- | --- | --- | --- | --- | --- | --- | --- | --- | --- | --- | --- | --- | --- | --- | --- | --- | --- | --- | --- | --- | --- | --- | --- | --- | --- | --- | --- | --- | --- | --- | --- | --- | --- | --- | --- | --- | --- | --- | --- | --- | --- | --- | --- | --- | --- | --- | --- | --- | --- | --- | --- | --- | --- | --- | --- | --- | --- | --- | --- | --- | --- | --- | --- | --- | --- | --- | --- | --- | --- | --- | --- | --- | --- | --- | --- | --- | --- | --- | --- | --- | --- | --- | --- | --- | --- | --- | --- | --- | --- | --- | --- | --- | --- | --- | --- | --- | --- | --- | --- | --- | --- | --- | --- | --- | --- | --- | --- | --- | --- | --- | --- | --- | --- | --- | --- | --- | --- | --- | --- | --- | --- | --- | --- | --- | --- | --- | --- | --- | --- | --- | --- | --- | --- | --- | --- | --- | --- | --- | --- | --- | --- | --- | --- | --- | --- | --- | --- | --- | --- | --- | --- | --- | --- | --- | --- | --- | --- | --- | --- | --- | --- | --- | --- | --- | --- | --- | --- | --- | --- | --- | --- | --- | --- | --- | --- | --- | --- | --- | --- | --- | --- | --- | --- | --- | --- | --- | --- | --- | --- | --- | --- | --- | --- | --- | --- | --- | --- | --- | --- | --- | --- | --- | --- | --- | --- | --- | --- | --- | --- | --- | --- | --- | --- | --- | --- | --- | --- | --- | --- | --- | --- | --- | --- | --- | --- | --- | --- | --- | --- | --- | --- | --- | --- | --- | --- | --- | --- | --- | --- | --- | --- | --- | --- | --- | --- | --- | --- | --- | --- | --- | --- | --- | --- | --- | --- | --- | --- | --- | --- | --- | --- | --- | --- | --- | --- | --- | --- | --- | --- | --- | --- | --- | --- | --- | --- | --- | --- | --- | --- | --- | --- | --- | --- | --- | --- | --- | --- | --- | --- | --- | --- | --- | --- | --- | --- | --- | --- | --- | --- | --- | --- | --- | --- | --- | --- | --- | --- | --- | --- | --- | --- | --- | --- | --- | --- | --- | --- | --- | --- | --- | --- | --- | --- | --- | --- | --- | --- | --- | --- | --- | --- | --- | --- | --- | --- | --- | --- | --- | --- | --- | --- | --- | --- | --- | --- | --- | --- | --- | --- | --- | --- | --- | --- | --- | --- | --- | --- | --- | --- | --- | --- | --- | --- | --- | --- | --- | --- | --- | --- | --- | --- | --- | --- | --- | --- | --- | --- | --- |
