# Supplementary material for: Complete genome sequence of Enterococcus faecium strain TX16 and comparative genomic analysis of Enterococcus faecium genomes
Source: BMC Microbiol. 2012 Jul 7;12:135. doi: 10.1186/1471-2180-12-135 (PMC3433357; doi:10.1186/1471-2180-12-135)
Supplement: Additional file 9 — Table S6.Summary of CRISPRs found inE. faeciumsequenced strains. A table listing in what strains CRISPRs were found, the locus tag, and the functional assignment. [file 1471-2180-12-135-S9.doc]

Supplemental Table-Summary of CRISPRs found in *E. faecium* sequenced strains.

| *E. faecium* strain | Locus Tag | Conserved domain/  functional assignment |
| --- | --- | --- |
|  |  |  |
| TX1330 | HMPREF0352_0914 | TIGR01865/*csn1* |
|  | HMPREF0352_0915 | TIGR03639/*cas1*_NMENI |
|  | HMPREF0352_0916 | COG3512/*cas2* |
|  | HMPREF0352_0917 | pfam09711/*csn2* |
| Com12^a^ | EFVG_01552 | TIGR01865/*csn1* |
|  | EFVG_01553 | TIGR03639/*cas1*_NMENI |
|  | EFVG_01554 | COG3512/*cas2* |
|  | EFVG_01555 | pfam09711/*csn2* |
| 1,141,733^a^ | EFSG_02406 | TIGR01865/*csn1* |
|  | EFSG_02407 | TIGR03639/*cas1*_NMENI |
|  | EFSG_02408 | COG3512/*cas2* |
|  | EFSG_02409 | pfam09711/*csn2* |
| 1,231,408^a^ | EFUG_01572 | TIGR01865/*csn1* |
|  | EFUG_01573 | TIGR03639/*cas1*_NMENI |
|  | EFUG_01574 | COG3512/*cas2* |
|  | EFUG_01575 | pfam09711/*csn2* |
|  |  |  |

^a^ Previously published (Palmer et al., 2010)
