# Supplementary material for: Complete genome sequence of Enterococcus faecium strain TX16 and comparative genomic analysis of Enterococcus faecium genomes
Source: BMC Microbiol. 2012 Jul 7;12:135. doi: 10.1186/1471-2180-12-135 (PMC3433357; doi:10.1186/1471-2180-12-135)
Supplement: Additional file 10 — Table S7.Specific enzymes present in TX16 but not inE. faecalisV583. A table listing enzymes, KEGG information, and locus tags specific to TX16. [file 1471-2180-12-135-S10.doc]

**Supplementary Table-Specific enzymes present in TX16 but not in *E. faecalis* V583**

**TX16 Locus tag KEGG information Enzymes**

HMPREF0351_10017 K00158 pyruvate oxidase [EC:1.2.3.3]

HMPREF0351_10030 K01220 6-phospho-beta-galactosidase [EC:3.2.1.85]

HMPREF0351_10031 K02786 PTS system, lactose-specific IIA component [EC:2.7.1.69]

HMPREF0351_10032 K02787 PTS system, lactose-specific IIB component [EC:2.7.1.69]

HMPREF0351_10055 K02358 elongation factor Tu

HMPREF0351_10101 K03685 ribonuclease III [EC:3.1.26.3]

HMPREF0351_10168 K01560 2-haloacid dehalogenase [EC:3.8.1.2]

HMPREF0351_10177 K03688 ubiquinone biosynthesis protein

HMPREF0351_10193 K01227 mannosyl-glycoprotein endo-beta-N-acetylglucosaminidase [EC:3.2.1.96]

HMPREF0351_10216 K00048 lactaldehyde reductase [EC:1.1.1.77]

HMPREF0351_10234 K01473 N-methylhydantoinase A [EC:3.5.2.14]

HMPREF0351_10261 K01580 glutamate decarboxylase [EC:4.1.1.15]

HMPREF0351_10286 K02584 Nif-specific regulatory protein

HMPREF0351_10336 K00121 S-(hydroxymethyl)glutathione dehydrogenase / alcohol dehydrogenase

[EC:1.1.1.284 1.1.1.1]

HMPREF0351_10352 K13935 malonate decarboxylase epsilon subunit [EC:2.3.1.39]

HMPREF0351_10367 K08302 tagatose 1,6-diphosphate aldolase [EC:4.1.2.40]

HMPREF0351_10379 K02757 PTS system, beta-glucosides-specific IIC component

HMPREF0351_10388 K00111 glycerol-3-phosphate dehydrogenase [EC:1.1.5.3]

HMPREF0351_10412 K01592 tyrosine decarboxylase [EC:4.1.1.25]

HMPREF0351_10432 K03186 3-octaprenyl-4-hydroxybenzoate carboxy-lyase UbiX [EC:4.1.1.-]

HMPREF0351_10433 K03182 3-octaprenyl-4-hydroxybenzoate carboxy-lyase UbiD [EC:4.1.1.-]

HMPREF0351_10457 K01226 trehalose-6-phosphate hydrolase [EC:3.2.1.93]

HMPREF0351_10464 K15519 deoxyadenosine/deoxycytidine kinase [EC:2.7.1.76 2.7.1.74]

HMPREF0351_10469 K00432 glutathione peroxidase [EC:1.11.1.9]

HMPREF0351_10483 K01451 hippurate hydrolase [EC:3.5.1.32]

HMPREF0351_10504 K01610 phosphoenolpyruvate carboxykinase (ATP) [EC:4.1.1.49]

HMPREF0351_10566 K11041 exfoliative toxin A/B

HMPREF0351_10693 K13038 phosphopantothenoylcysteine decarboxylase / phosphopantothenate--cysteine ligase

[EC:4.1.1.36 6.3.2.5]

HMPREF0351_10695 K00001 alcohol dehydrogenase [EC:1.1.1.1]

HMPREF0351_10715 K00549 5-methyltetrahydropteroyltriglutamate--homocysteine methyltransferase [EC:2.1.1.14]

HMPREF0351_10726 K02770 PTS system, fructose-specific IIC component

HMPREF0351_10752 K03739 membrane protein involved in D-alanine export

HMPREF0351_10754 K03740 D-alanine transfer protein

HMPREF0351_10755 K01179 endoglucanase [EC:3.2.1.4]

HMPREF0351_10791 K08724 penicillin-binding protein 2B

HMPREF0351_10795 K03589 cell division protein FtsQ

HMPREF0351_10796 K03590 cell division protein FtsA

HMPREF0351_10797 K03531 cell division protein FtsZ

HMPREF0351_10903 K09691 lipopolysaccharide transport system ATP-binding protein

HMPREF0351_10916 K01654 N-acetylneuraminate synthase [EC:2.5.1.56]

HMPREF0351_10922 K00983 N-acylneuraminate cytidylyltransferase [EC:2.7.7.43]

HMPREF0351_10941 K00058 D-3-phosphoglycerate dehydrogenase [EC:1.1.1.95]

HMPREF0351_10945 K04564 superoxide dismutase, Fe-Mn family [EC:1.15.1.1]

HMPREF0351_11011 K02803 PTS system, N-acetylglucosamine-specific IIB component [EC:2.7.1.69]

HMPREF0351_11012 K01232 maltose-6'-phosphate glucosidase [EC:3.2.1.122]

HMPREF0351_11072 K00626 acetyl-CoA C-acetyltransferase [EC:2.3.1.9]

HMPREF0351_11081 K02358 elongation factor Tu

HMPREF0351_11082 K14205 phosphatidylglycerol lysyltransferase [EC:2.3.2.3]

HMPREF0351_11116 K00974 tRNA nucleotidyltransferase (CCA-adding enzyme) [EC:2.7.7.72 3.1.3.- 3.1.4.-]

HMPREF0351_11165 K15599 putative hydroxymethylpyrimidine transport system permease protein

HMPREF0351_11166 K15598 putative hydroxymethylpyrimidine transport system substrate-binding protein

HMPREF0351_11167 K15600 putative hydroxymethylpyrimidine transport system ATP-binding protein

HMPREF0351_11182 K11692 two-component system, CitB family, response regulator DctR

HMPREF0351_11183 K07701 two-component system, CitB family, sensor histidine kinase DcuS [EC:2.7.13.3]

HMPREF0351_11186 K07192 flotillin

HMPREF0351_11220 K10546 putative multiple sugar transport system substrate-binding protein

HMPREF0351_11221 K10548 putative multiple sugar transport system ATP-binding protein

HMPREF0351_11222 K10547 putative multiple sugar transport system permease protein

HMPREF0351_11234 K03588 cell division protein FtsW

HMPREF0351_11285 K10041 putative glutamine transport system ATP-binding protein [EC:3.6.3.-]

HMPREF0351_11303 K00784 ribonuclease Z [EC:3.1.26.11]

HMPREF0351_11311 K00615 transketolase [EC:2.2.1.1]

HMPREF0351_11323 K05366 penicillin-binding protein 1A [EC:2.4.1.- 3.4.-.-]

HMPREF0351_11330 K00639 glycine C-acetyltransferase [EC:2.3.1.29]

HMPREF0351_11409 K02448 nitric oxide reductase NorD protein

HMPREF0351_11449 K00040 fructuronate reductase [EC:1.1.1.57]

HMPREF0351_11459 K03588 cell division protein FtsW

HMPREF0351_11466 K01662 1-deoxy-D-xylulose-5-phosphate synthase [EC:2.2.1.7]

HMPREF0351_11470 K01425 glutaminase [EC:3.5.1.2]

HMPREF0351_11512 K00058 D-3-phosphoglycerate dehydrogenase [EC:1.1.1.95]

HMPREF0351_11518 K05993 isochorismatase [EC:3.3.2.1]

HMPREF0351_11536 K13993 HSP20 family protein

HMPREF0351_11543 K03596 GTP-binding protein LepA

HMPREF0351_11581 K10190 lactose/L-arabinose transport system permease protein

HMPREF0351_11582 K10189 lactose/L-arabinose transport system permease protein

HMPREF0351_11583 K10188 lactose/L-arabinose transport system substrate-binding protein

HMPREF0351_11597 K07407 alpha-galactosidase [EC:3.2.1.22]

HMPREF0351_11611 K02770 PTS system, fructose-specific IIC component

HMPREF0351_11618 K03544 ATP-dependent Clp protease ATP-binding subunit ClpX

HMPREF0351_11625 K03431 phosphoglucosamine mutase [EC:5.4.2.10]

HMPREF0351_11642 K01779 aspartate racemase [EC:5.1.1.13]

HMPREF0351_11669 K01621 phosphoketolase [EC:4.1.2.9]

HMPREF0351_11680 K11749 regulator of sigma E protease [EC:3.4.24.-]

HMPREF0351_11688 K03430 2-aminoethylphosphonate-pyruvate transaminase [EC:2.6.1.37]

HMPREF0351_11690 K05306 phosphonoacetaldehyde hydrolase [EC:3.11.1.1]

HMPREF0351_11718 K01953 asparagine synthase (glutamine-hydrolysing) [EC:6.3.5.4]

HMPREF0351_11727 K01607 4-carboxymuconolactone decarboxylase [EC:4.1.1.44]

HMPREF0351_11799 K02810 PTS system, sucrose-specific IIC component

HMPREF0351_11895 K00812 aspartate aminotransferase [EC:2.6.1.1]

HMPREF0351_11943 K00971 mannose-1-phosphate guanylyltransferase [EC:2.7.7.22]

HMPREF0351_11944 K02377 GDP-L-fucose synthase [EC:1.1.1.271]

HMPREF0351_11945 K01711 GDPmannose 4,6-dehydratase [EC:4.2.1.47]

HMPREF0351_11954 K00718 galactoside 2-L-fucosyltransferase 1/2 [EC:2.4.1.69]

HMPREF0351_11959 K00012 UDPglucose 6-dehydrogenase [EC:1.1.1.22]

HMPREF0351_11993 K02757 PTS system, beta-glucosides-specific IIC component

HMPREF0351_12164 K10118 multiple sugar transport system permease protein

HMPREF0351_12171 K00791 tRNA dimethylallyltransferase [EC:2.5.1.75]

HMPREF0351_12174 K01358 ATP-dependent Clp protease, protease subunit [EC:3.4.21.92]

HMPREF0351_12211 K01101 4-nitrophenyl phosphatase [EC:3.1.3.41]

HMPREF0351_12244 K12266 anaerobic nitric oxide reductase transcription regulator

HMPREF0351_12309 K02800 PTS system, mannitol-specific IIC component

HMPREF0351_12331 K00058 D-3-phosphoglycerate dehydrogenase [EC:1.1.1.95]

HMPREF0351_12332 K01804 L-arabinose isomerase [EC:5.3.1.4]

HMPREF0351_12343 K01209 alpha-N-arabinofuranosidase [EC:3.2.1.55]

HMPREF0351_12406 K07778 two-component system, NarL family, sensor histidine kinase DesK [EC:2.7.13.3]

HMPREF0351_12437 K00517 [EC:1.14.-.-]

HMPREF0351_12476 K01198 xylan 1,4-beta-xylosidase [EC:3.2.1.37]

HMPREF0351_12488 K00041 tagaturonate reductase [EC:1.1.1.58]

HMPREF0351_12489 K01685 altronate hydrolase [EC:4.2.1.7]

HMPREF0351_12490 K01812 glucuronate isomerase [EC:5.3.1.12]

HMPREF0351_12493 K05658 ATP-binding cassette, subfamily B (MDR/TAP), member 1

HMPREF0351_12626 K02804 PTS system, N-acetylglucosamine-specific IIC component

HMPREF0351_12649 K02871 large subunit ribosomal protein L13

HMPREF0351_12663 K00690 sucrose phosphorylase [EC:2.4.1.7]

HMPREF0351_12678 K03885 NADH dehydrogenase [EC:1.6.99.3]

HMPREF0351_12683 K08303 putative protease [EC:3.4.-.-]

HMPREF0351_12882 K10985 PTS system, galactosamine-specific IIC component

HMPREF0351_12919 K10986 PTS system, galactosamine-specific IID component

HMPREF0351_12921 K02757 PTS system, beta-glucosides-specific IIC component

HMPREF0351_12988 K01197 hyaluronoglucosaminidase [EC:3.2.1.35]

HMPREF0351_13004 K10230 sorbitol/mannitol transport system ATP-binding protein

HMPREF0351_13006 K05814 sn-glycerol 3-phosphate transport system permease protein

HMPREF0351_13037 K02779 PTS system, glucose-specific IIC component

HMPREF0351_13046 K11440 choline dehydrogenase [EC:1.1.1.1]

HMPREF0351_13047 K03336 3D-(3,5/4)-trihydroxycyclohexane-1,2-dione hydrolase [EC:3.7.1.-]

HMPREF0351_13048 K03338 5-dehydro-2-deoxygluconokinase [EC:2.7.1.92]

HMPREF0351_13049 K03337 5-deoxy-glucuronate isomerase [EC:5.3.1.-]

HMPREF0351_13061 K10189 lactose/L-arabinose transport system permease protein
