# Supplementary material for: Complete genome sequence of Enterococcus faecium strain TX16 and comparative genomic analysis of Enterococcus faecium genomes
Source: BMC Microbiol. 2012 Jul 7;12:135. doi: 10.1186/1471-2180-12-135 (PMC3433357; doi:10.1186/1471-2180-12-135)
Supplement: Additional file 11 — Table S8.Specific enzymes present inE. faecalis V583 but not in TX16. A table listing the enzymes and locus tags specific to V583. [file 1471-2180-12-135-S11.doc]

**Supplementary Table-Specific enzymes present in *E. faecalis* V583 but not in TX16**

**V583 ID KEGG information Enzymes**

EF0028 K02790 bifunctional PTS system maltose and glucose-specific transporter subunits IICB

EF0042 K01770 ispF; 2C-methyl-D-erythritol 2,4-cyclodiphosphate synthase

EF0171 K01488 adenosine deaminase [EC:3.5.4.4]

EF0194 K00100 NADH-dependent butanol dehydrogenase

EF0230 K02919 large subunit ribosomal protein L36

EF0388 K00073 allD; ureidoglycolate dehydrogenase

EF0390 K01485 N-acyl-D-amino-acid deacylase family protein

EF0406 K02799 PTS system, IIBC component

EF0411 K02799 PTS system mannitol-specfic transporter subunit IIBC

EF0445 K01661 menB; naphthoate synthase (EC:4.1.3.36)

EF0446 K01911 O-succinylbenzoic acid--CoA ligase (EC:6.2.1.26)

EF0447 K02552 menaquinone-specific isochorismate synthase

EF0448 K02551 menD; 2-succinyl-6-hydroxy-2,4-cyclohexadiene-1-carboxylic acid synthase/2-oxoglutarate

decarboxylase

EF0449 K08680 alpha/beta fold family hydrolase

EF0450 K02549 mandelate racemase/muconate lactonizing enzyme family protein

EF0556 K01805 xylA; xylose isomerase (EC:5.3.1.5)

EF0557 K00854 xylB; D-xylulose kinase

EF0567 K01546 kdpA; potassium-transporting ATPase subunit A (EC:3.6.3.12)

EF0568 K01547 kdpB; potassium-transporting ATPase subunit B (EC:3.6.3.12)

EF0569 K01548 kdpC; potassium-transporting ATPase subunit C

EF0570 K07646 kdpD; sensor histidine kinase KdpD

EF0571 K07667 DNA-binding response regulator

EF0694 K02769 PTS system fructose-specific transporter subunit IIBC

EF0734 K10536 agmatine deiminase

EF0991 K12556 pbpC; penicillin-binding protein C

EF1024 K01006 ppdK; pyruvate phosphate dikinase (EC:2.7.9.1)

EF1037 K09758 aspartate aminotransferase (EC:2.6.1.1)

EF1086 K00657 spermine/spermidine acetyltransferase

EF1100 K06148 ABC transporter ATP-binding protein/permease

EF1134 K05823 N-acetyldiaminopimelate deacetylase [EC:3.5.1.47]

EF1148 K05366 penicillin-binding protein 1A [EC:2.4.1.- 3.4.-.-]

EF1175 K00980 gct; glycerol-3-phosphate cytidylyltransferase

EF1206 K00028 malate dehydrogenase, decarboxylating

EF1236 K01060 acetyl xylan esterase

EF1238 K05349 glycosyl hydrolase family protein

EF1364 K00054 acetyl-CoA acetyltransferase/hydroxymethylglutaryl-CoA reductase, degradative

EF1388 K00334 NAD-dependent formate dehydrogenase subunit gamma

EF1389 K00335 NAD-dependent formate dehydrogenase subunit beta

EF1390 K00123 fdhA; NAD-dependent formate dehydrogenase subunit alpha

EF1392 K03637 moaC; molybdenum cofactor biosynthesis protein MoaC

EF1393 K03639 molybdopterin cofactor biosynthesis protein A

EF1495 K02121 V-type H+transporting ATPase subunit E [EC:3.6.3.14]

EF1516 K02802 PTS system, IIABC components

EF1586 K00356 nox; NADH oxidase

EF1597 K03781 katA; catalase/peroxidase

EF1601 K02808 PTS system, IIABC components

EF1627 K03736 eutC; ethanolamine ammonia-lyase small subunit (EC:4.3.1.7)

EF1629 K03735 eutB; ethanolamine ammonia-lyase large subunit

EF1658 K09699 bkdC; branched-chain alpha-keto acid, E2 component, dihydrolipoamide acetyltransferase

EF1659 K00167 bkdB; branched-chain alpha-keto acid dehydrogenase, E1 component subunit beta

EF1660 K00166 bkdA; branched-chain alpha-keto acid dehydrogenase, E1 component subunit alpha

EF1662 K00929 buk; butyrate kinase (EC:2.7.2.7)

EF1663 K00634 ptb; branched-chain phosphotransacylase

EF1821 K07813 agrBfs; agrBfs protein

EF1826 K13953 adhA; alcohol dehydrogenase

EF1834 K01819 lacB; galactose-6-phosphate isomerase subunit LacB (EC:5.3.1.26)

EF1835 K01819 lacA; galactose-6-phosphate isomerase subunit LacA (EC:5.3.1.26)

EF1928 K00105 alpha-glycerophosphate oxidase [EC:1.1.3.21]

EF1958 K01129 deoxyguanosinetriphosphate triphosphohydrolase-like protein

EF1989 K01772 hemH; ferrochelatase

EF2058 K06148 transport ATP-binding protein CydD

EF2059 K06148 transport ATP-binding protein CydC

EF2060 K00426 cydB; cytochrome d ubiquinol oxidase subunit II

EF2061 K00425 cydA; cytochrome d ubiquinol oxidase subunit I

EF2150 K12554 FemAB family protein

EF2172 K00991 ispD; 2-C-methyl-D-erythritol 4-phosphate cytidylyltransferase

EF2267 K02744 PTS system, IIA component

EF2269 K02747 PTS system, IID component

EF2270 K02746 PTS system, IIC component

EF2271 K02745 PTS system, IIB component

EF2294 K15739 D-alanine---(R)-lactate ligase [EC:6.1.2.1]

EF2420 K00872 thrB; homoserine kinase (EC:2.7.1.39)

EF2421 K01733 thrC; threonine synthase (EC:4.2.3.1)

EF2422 K00003 hom; homoserine dehydrogenase (EC:1.1.1.3)

EF2425 K01835 phosphoglucomutase [EC:5.4.2.2]

EF2431 K01487 chlorohydrolase family protein

EF2481 K01091 HAD superfamily hydrolase

EF2486 K09693 ABC transporter ATP-binding protein

EF2560 K00266 glutamate synthase (NADPH/NADH) small chain [EC:1.4.1.13 1.4.1.14]

EF2567 K01008 selD; selenide, water dikinase

EF2580 K01464 phenylhydantoinase (EC:3.5.2.2)

EF2581 K12527 selenate reductase subunit YgfK

EF2656 K01598 phosphopantothenoylcysteine decarboxylase (EC:4.1.1.36)

EF2658 K05363 FemAB family protein

EF2730 K03073 secE; preprotein translocase subunit SecE

EF2767 K03707 transcriptional regulator

EF2776 K00788 thiE; thiamine-phosphate pyrophosphorylase

EF2777 K00878 hydroxyethylthiazole kinase

EF2883 K02371 fabK; enoyl-ACP reductase

EF2916 K01091 HAD superfamily hydrolase

EF2996 K14977 hypothetical protein

EF2999 K01466 allantoinase (EC:3.5.2.5)

EF3042 K02747 PTS system, IID component

EF3043 K02746 PTS system, IIC component

EF3045 K02745 PTS system, IIB component

EF3046 K02744 PTS system, IIA component

EF3193 K05339 antiholin-like protein LrgB

EF3194 K05338 murein hydrolase regulator LrgA

EF3196 K07705 response regulator

EF3197 K07704 sensor histidine kinase

EF3265 K00796 folP; dihydropteroate synthase

EF3267 K01495 folE; GTP cyclohydrolase I (EC:3.5.4.16)

EF3268 K00950 folK; 2-amino-4-hydroxy-6-hydroxymethyldihydropteridine pyrophosphokinase

EF3269 K01633 folB; dihydroneopterin aldolase

EF3317 K01571 oxaloacetate decarboxylase (EC:4.1.1.3)

EF3318 K05964 citX; 2'-(5''-triphosphoribosyl)-3'-dephospho-CoA:apo-citrate lyase (EC:2.7.7.61)

EF3319 K01643 citF; citrate lyase subunit alpha

EF3320 K01644 citE; citrate lyase subunit beta

EF3321 K01646 citD; citrate lyase subunit gamma

EF3322 K01910 citC; citrate lyase ligase

EFA0067 K02808 PTS system, IIABC components
